# Benzothiazolium salts as reagents for the deoxygenative perfluoroalkylthiolation of alcohols

**DOI:** 10.3762/bjoc.17.8

**Published:** 2021-01-08

**Authors:** Armin Ariamajd, Nils J Gerwien, Benjamin Schwabe, Stefan Dix, Matthew N Hopkinson

**Affiliations:** 1 Institute of Chemistry and Biochemistry, Freie Universität Berlin, Fabeckstrasse 34–36, 14195 Berlin, Germany

**Keywords:** alcohols, benzothiazolium salts, deoxygenative reactions, fluorine, perfluoroalkylthiolation, thioethers

## Abstract

A series of 2-(perfluoroalkylthio)benzothiazolium (BT-SR_F_) salts have been synthesized that serve as convenient sources of hitherto underexplored perfluoroalkylthiolate anions. An investigation of their reactivity in a deoxygenative nucleophilic substitution reaction led to the development of an unprecedented process that provides pentafluoroethyl and heptafluoropropyl thioethers directly from readily available alcohols.

## Introduction

The incorporation of fluorine-containing groups into organic molecules to modulate their biological or physical properties is nowadays a common strategy employed in the development of pharmaceuticals [1–4], agrochemicals [5] and materials [6]. Recent years have seen a surge in interest in emerging fluorinated motifs, which can offer improved performance over single fluorine atoms or perfluoroalkyl substituents. The trifluoromethylthio group (SCF_3_), for example, is attracting considerable attention due to its strong lipophilicity-enhancing influence (Hansch constant π = 1.44) and electron-withdrawing properties (Hammett constants σ_p_ = 0.50 and σ_m_ = 0.40) [7–12]. On the other hand, longer-chain perfluoroalkylthio groups (SR_F_, R_F_ = C*_n_*F_2_*_n_*_+1_) have received comparatively little attention despite promising applications in liquid crystal displays [13–14], as pharmaceuticals and agrochemicals [15]. For example, analogues of the drug losartan featuring SC_2_F_5_, SC_3_F_7_ and SC_4_F_9_ groups have shown promise as treatments for hypertensive disorders [16]. Furthermore, in a number of drug candidates, exchanging a CF_3_ group with C_2_F_5_ has been shown to result in superior pharmacological properties; however, analogous studies comparing SCF_3_ with SC_2_F_5_ are scarce [17–19].

**Scheme 1:**
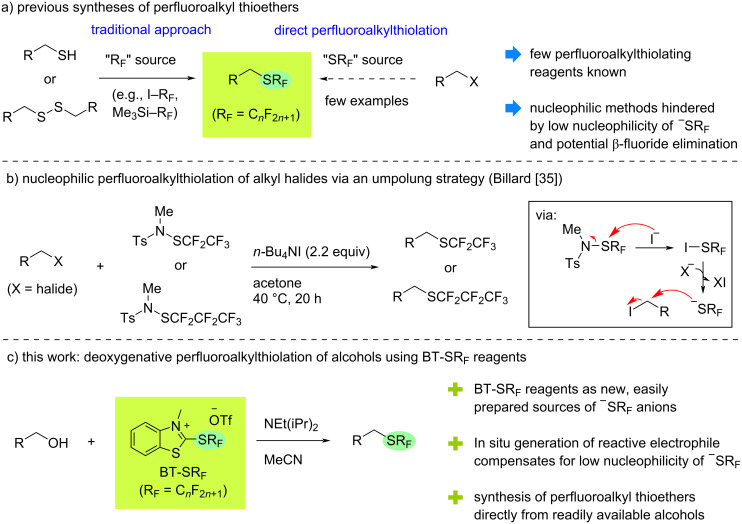
Synthetic routes towards perfluoroalkyl thioethers.

Much of the recent interest in the SCF_3_ group can be attributed to the development of several bench-stable and easily handled trifluoromethylthiolating reagents, which do not require specialist equipment or expertise [7–12]. Synthetic approaches towards longer-chain perfluoroalkylthio-substituted molecules, on the other hand, remain limited. In the vast majority of cases, the SR_F_ group is not installed as an intact functional group but rather indirectly through perfluoroalkylation of a thiol or sulfide moiety already present on the substrate (Scheme 1a) [15,20–26]. By contrast, direct perfluoroalkylthiolation, which avoids pre-functionalization of the substrate, has been hindered by the lack of suitable reagents. In recent years, however, a selection of mostly electrophilic and radical sources has been introduced [27]. Nucleophilic perfluoroalkylthiolation is especially challenging due to the low nucleophilicity of heavily fluorinated thiolate anions and the potential for deleterious side-reactions resulting from β-fluoride elimination [28–34]. Only a handful of perfluoroalkylthiolate salts are known and, to the best of our knowledge, only one general direct nucleophilic perfluoroalkylthiolation of an alkyl electrophile has been reported to date [35]. This process used an umpolung strategy with activation of typically electrophilic perfluoroalkylsulfenamide reagents by iodide, releasing ^−^SC_2_F_5_ or ^−^SC_3_F_7_ anions in situ, which could then react with a selection of alkyl halides (Scheme 1b).

In 2019, we reported a new reagent for nucleophilic trifluoromethylthiolation based on the benzothiazolium motif: BT-SCF_3_ [36–38]. This easily handled solid, which is stable at least over several months under ambient conditions, could be engaged in efficient deoxygenative trifluoromethylthiolation reactions of readily available aliphatic alcohols. Some features of this process led us to consider whether related benzothiazolium salts featuring longer-chain SR_F_ substituents could serve as practical reagents for investigating nucleophilic perfluoroalkylthiolation reactions. Firstly, the simple two-step synthesis of BT-SCF_3_ from inexpensive 2-mercaptobenzothiazole (MBT) can be readily adapted to provide a wide range of different BT-SR_F_ reagents. Moreover, in situ activation of the benzothiazolium salt by the alcohol provides a highly reactive alkyl electrophile, which can compensate for the inherently low nucleophilicity of ^−^SR_F_ anions [39]. Herein, we report the successful synthesis of several BT-SR_F_ reagents and provide insights into the reactivity of different perfluoroalkylthiolate anions in nucleophilic substitution reactions. This study led to the development of unprecedented deoxygenative pentafluoroethyl- and heptafluoropropylthiolation reactions directly from alcohols (Scheme 1c).

## Results and Discussion

**Scheme 2:**
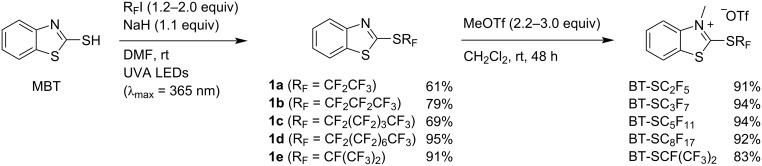
Two-step synthesis of BT-SR_F_ reagents from MBT.

Five BT-SR_F_ reagents were selected to provide a good overview on the reactivity of different perfluoroalkylthio groups; four linear BT-SR_F_ derivatives (BT-SC_2_F_5_, BT-SC_3_F_7_, BT-SC_5_F_11_ and BT-SC_8_F_17_) and the perfluoroisopropyl species BT-SCF(CF_3_)_2_. Each reagent was synthesized according to the two-step procedure shown in Scheme 2. Firstly, MBT was reacted with a perfluoroalkyl iodide and NaH in DMF under irradiation with UVA LEDs (λ_max_ = 365 nm). In all cases, efficient *S*-perfluoroalkylation was observed within 16 h at rt, and the heteroaromatic compounds **1a–e** could be isolated in good yields. Subsequent *N*-methylation with methyl trifluoromethanesulfonate in CH_2_Cl_2_ also proceeded smoothly with the BT-SR_F_ reagents each being obtained after 48 h at rt in high yields upon precipitation with Et_2_O. As with BT-SCF_3_, further purification of the reagents was not required and each was found to be stable at least over several months when stored in a fridge.

**Scheme 3:**
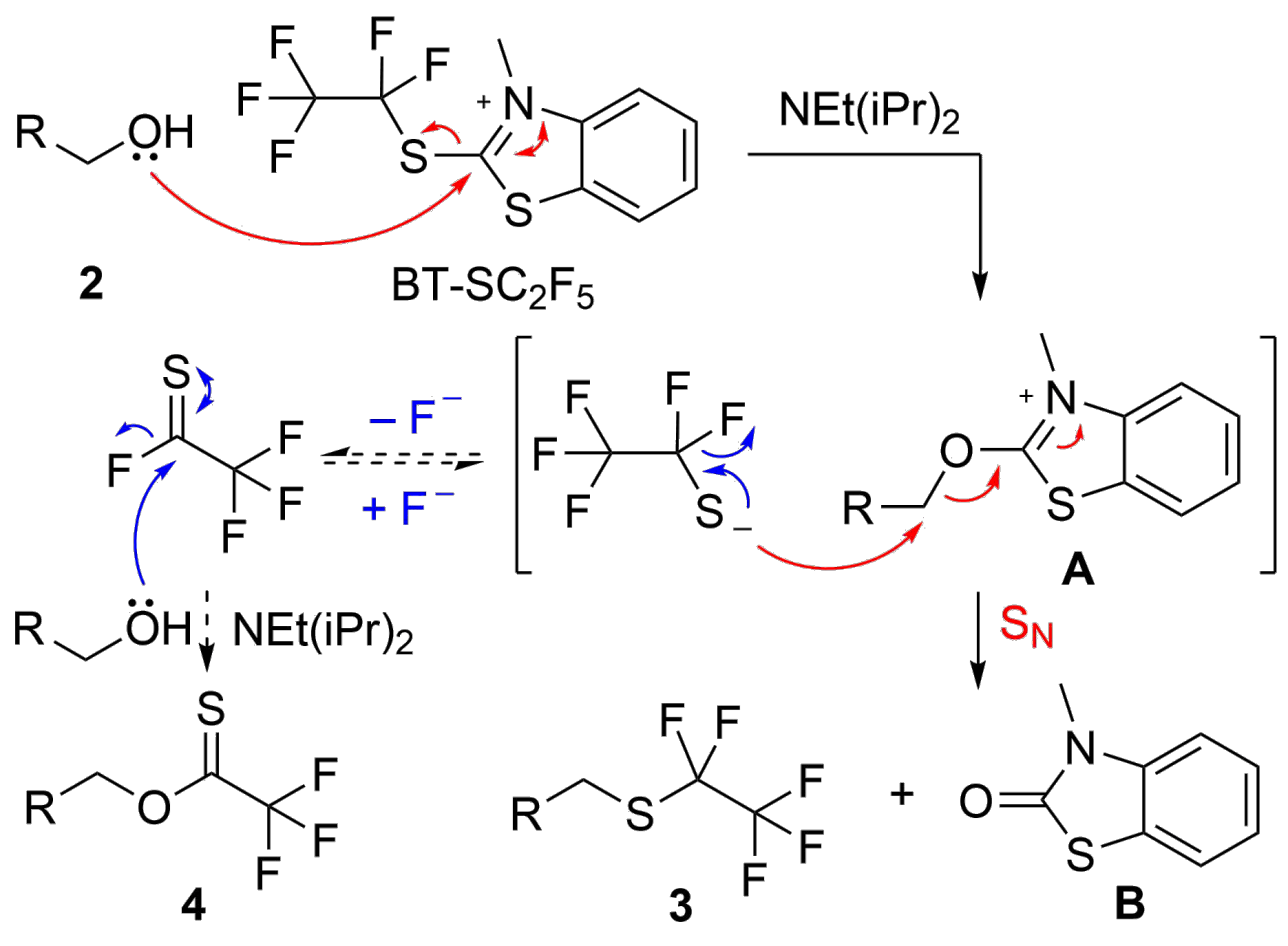
Plausible mechanism for the formation of thioether **3** and thionoester **4**.

With a selection of BT-SR_F_ reagents in hand, we next sought to evaluate their relative reactivity in a benchmark nucleophilic perfluoroalkylthiolation. Thus, 4-nitrobenzyl alcohol (**2a**) was reacted with 1.25 equivalents of BT-SC_2_F_5_ and 2 equivalents of NEt(iPr)_2_ in MeCN for 2 h at rt. The analysis of the crude reaction mixture by NMR revealed the formation of two products: the desired (pentafluoroethyl) thioether **3a** in 78% NMR yield and a second species with ^19^F and ^1^H spectra consistent with the trifluoromethyl thionoester **4a** (7% NMR yield, Table 1, entry 1). The formation of both species can be explained by the mechanism shown in Scheme 3 [36]. Nucleophilic attack of the alcohol in the presence of NEt(iPr)_2_ at the C2-position of the BT reagent affords the key electrophilic 2-alkoxybenzothiazolium species **A** and the perfluoroalkylthiolate anion. Nucleophilic substitution then affords product **3** and thiocarbamate byproduct **B**. As a side-reaction, β-fluoride elimination from the perfluoroalkylthiolate anion can occur, leading to a thiocarbonyl fluoride species, which can subsequently react with the alcohol, delivering thionoester **4**. β-Fluoride elimination is a known decomposition pathway of ^−^SCF_3_ and has even been exploited in synthetic trifluoromethylthiolation and fluorination processes [32–34].

**Table 1:** Deoxygenative perfluoroalkylthiolation of alcohol **2a** with BT-SR_F_ reagents.^a^

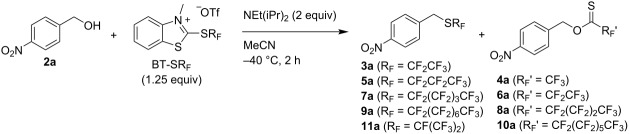			

Entry	R_F_	^1^H NMR yield of thioether	^1^H NMR yield of thionoester

1^b^	CF_2_CF_3_	**3a** (78%)	**4a** (7%)
2	CF_2_CF_3_	**3a** (99%)	**4a** (trace)
3	CF_2_CF_2_CF_3_	**5a** (82%)	**6a** (15%)
4	CF_2_(CF_2_)_3_CF_3_	**7a** (74%)	**8a** (26%)
5	CF_2_(CF_2_)_6_CF_3_	**9a** (46%)	**10a** (47%)
6	CF(CF_3_)_2_	**11a** (trace)	–

^a^Reaction conditions: BT-SR_F_ (1.25 equiv), NEt(iPr)_2_ (2 equiv), MeCN (0.17 M), −40 °C, 2 h. ^b^Reaction conducted at rt.

The formation of the side-product **4a** could be effectively suppressed by conducting the reaction at −40 °C. Under these conditions, the desired (pentafluoroethyl) thioether **3a** was obtained in 99% NMR yield (91% isolated yield, Table 1, entry 2). At this stage, we were ready to evaluate the relative efficiency of different perfluoroalkylthiolate nucleophiles. Each of the other BT-SR_F_ reagents (1.25 equiv) was reacted with alcohol **2a** and NEt(iPr)_2_ (2 equiv) in MeCN at −40 °C and, after 2 h, the crude mixture was analysed by ^1^H and ^19^F NMR. As shown in Table 1, a significant decrease in the nucleophilic perfluoroalkylthiolation efficiency was observed upon increasing the R_F_ chain length (Table 1, entries 2–5). For example, thionoester **8a** was generated as a significant side product (26% NMR yield) when using BT-SC_5_F_11_, while thioether **9a** and thionoester **10a** were provided in almost equal amounts when BT-SC_8_F_17_ was reacted with **2a**. The increased prevalence of the β-fluoride elimination pathway with longer perfluoroalkylthio chains likely reflects the lower nucleophilicity of the increasingly fluorinated thiolate anions. Moreover, (perfluoroisopropyl) thioether **11a** was produced in only trace amounts in the reaction with BT-SCF(CF_3_)_2_, which is consistent with the expected lower nucleophilicity of the sterically more encumbered anion (Table 1, entry 6).

The efficient perfluoroalkylthiolation of **2a** observed with BT-SC_2_F_5_ led us to further investigate this approach as a route towards (pentafluoroethyl) thioethers. Deoxygenative substitution reactions of this type are synthetically appealing as they avoid pre-generation of an active electrophile such as an alkyl halide.

**Scheme 4:**
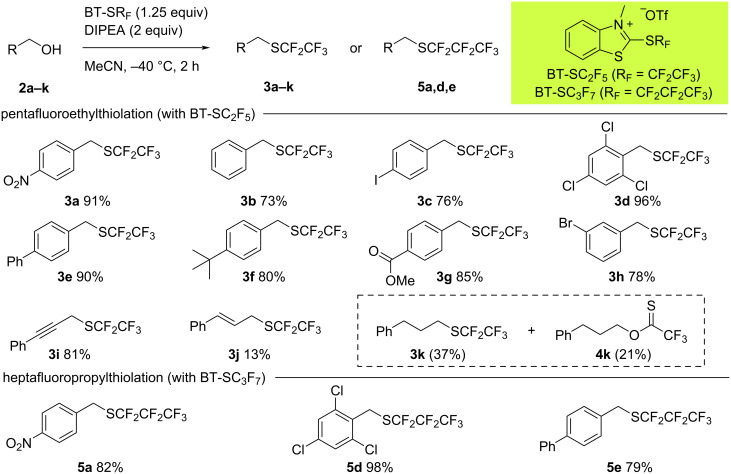
Scope of the deoxygenative perfluoroalkylthiolation reaction using BT-SC_2_F_5_ and BT-SC_3_F_7_.

A selection of benzylic alcohols **2** was reacted under the standard conditions with BT-SC_2_F_5_ (1.25 equiv) and NEt(iPr)_2_ (2 equiv) in MeCN at −40 °C, providing the corresponding (pentafluoroethyl) thioethers **3** in generally high yields (Scheme 4). A wide range of functional groups were tolerated, including the halogens Cl, Br, and I, thus opening the door for further functionalization of the products via cross-coupling. The propargylic alcohol substrate **2i** could also be converted into thioether **3i** in 81% yield, although the allylic alcohol **2j** was less efficient (13% yield of **3j**). The primary aliphatic (pentafluoroethyl) thioether **3k** could also be obtained in the reaction between alcohol **2k** and BT-SC_2_F_5_, however, with this less activated substrate, the thionoester **4k** was generated as a significant side product. Finally, the suitability of this methodology for the preparation of (heptafluoropropyl) thioethers was investigated by reacting a selection of benzylic alcohols **2** with BT-SC_3_F_7_. As for the pentafluoroethylthiolation reactions, efficient conversion was observed with products **5** being obtained in high yields of up to 98%.

## Conclusion

In conclusion, a series of bench-stable and easy to handle 2-(perfluoroalkylthio)benzothiazolium (BT-SR_F_) salts have been synthesized and investigated as reagents for the direct nucleophilic perfluoroalkylthiolation of aliphatic alcohols. While β-fluoride elimination was a significant side reaction with longer-chain SR_F_ groups, efficient deoxygenative functionalization was observed with BT-SC_2_F_5_ and BT-SC_3_F_7_. This synthetically attractive reaction provides pentafluoroethyl and heptafluoropropyl thioethers directly from readily available alcohols, thus avoiding the pre-formation of an alkyl halide. This straight-forward route should inspire further investigations of perfluoroalkylthio groups in pharmaceutical, agrochemical and materials science.

## Supporting Information

The Supporting Information contains experimental procedures, characterization data of all BT-SR_F_ reagents and isolated products as well as copies of NMR spectra.

File 1 Experimental section.
