# Supplementary material for: Benzothiazolium salts as reagents for the deoxygenative perfluoroalkylthiolation of alcohols
Source: Beilstein J Org Chem. 2021 Jan 8;17:83–8. doi: 10.3762/bjoc.17.8 (PMC7801780; doi:10.3762/bjoc.17.8)
Supplement: File 1 — Experimental section. [file Beilstein_J_Org_Chem-17-83-s001.pdf]

BEILSTEIN JOURNAL OF ORGANIC CHEMISTRY

Supporting Information

for

Benzothiazolium salts as reagents for the deoxygenative perfluoroalkylthiolation of alcohols

Armin Ariamajd, Nils J. Gerwien, Benjamin Schwabe, Stefan Dix and Matthew N. Hopkinson

Beilstein J. Org. Chem. **2021,** *17,* 83–88. doi:10.3762/bjoc.17.8

Experimental section

License and Terms: This is a supporting information file under the terms of the Creative Commons Attribution License (https://creativecommons.org/licenses/by/4.0). Please note that the reuse, redistribution and reproduction in particular requires that the author(s) and source are credited and that individual graphics may be subject to special legal provisions.
The license is subject to the *Beilstein Journal of Organic Chemistry* terms and conditions: (https://www.beilstein-journals.org/bjoc/terms)

Contents

1 General Information S2

2 Synthesis of BT-SR_F Reagents S3

2.1 Perfluoroalkylation of MBT with Perfluoroalkyl Iodides S3

2.2 *N*-Methylation of 2-((Perfluoroalkyl)thio)benzo[*d*]thiazoles 1 S6

3 Scope and Limitations of the Deoxyperfluoroalkylthiolation of Alcohols S9

3.1 Deoxyperfluoroalkylation of 4-Nitrobenzyl alcohol (2a) with Different BT-SR_F Reagents S9

3.2 Deoxypentafluoroethylation of Alcohols 2 with BT-SC_2F_5 S16

3.3 Deoxyheptafluoropropylation of Alcohols 2 with BT-SC_3F_7 S21

4 NMR Spectra of Novel Compounds S22

1 General Information

Solvents were purified either with the solvent purification system MB-SPS-800 (Braun) or by manual distillation over standard drying agents. The dried solvents were then stored over molecular sieves or transferred under argon. UVA light irradiation for the synthesis of the reagents was provided by a LED lamp (λ_{max} = 365 nm, IP65). All compounds employed were purchased from commercial suppliers and used as received. The deoxygenative perfluoroalkylthiolation reactions were performed in round-bottom flasks under an atmosphere of air. Flash chromatography was performed using silica gel.

NMR spectra were acquired on a JEOL ECX 400 (400 MHz), JEOL ECP 500/ Bruker Avance 500 (500 MHz), Varian INOVA 600 (600 MHz) or a Bruker Avance 700 (700 MHZ) in $CDCl_3$ or CD_3CN as a solvent. Chemical shifts (δ) are quoted in ppm downfield of tetramethylsilane. The residual solvent signals were used as references for ^{1}H and ^{13}C NMR spectra. ^{19}F NMR spectra are not calibrated by an internal reference and coupling constants ($J_{F\text{-}H}$) where reported were determined from proton coupled ^{19}F NMR studies. Coupling constants (J) are quoted in Hz. ^{1}H NMR yields where reported were measured using CH_2Br_2 as an internal standard.

Mass spectra were obtained on a ESI-FTICR-MS: Ionspec QFT-7 (Agilent/Varian) or on a HR-EI-MS: Waters Autospec Premier with Agilent 7890B GC. Infrared spectra were measured on a Thermo Scientific Nicolet iS10 FT-IR Spectrometer. Characteristic absorption bands are displayed in wavelengths $\tilde{\nu}$ in cm^{-1} and were analyzed with the software Spectral Manager from JASCO.

2 Synthesis of BT-SR_F Reagents

2.1 Perfluoroalkylation of MBT with Perfluoroalkyl Iodides

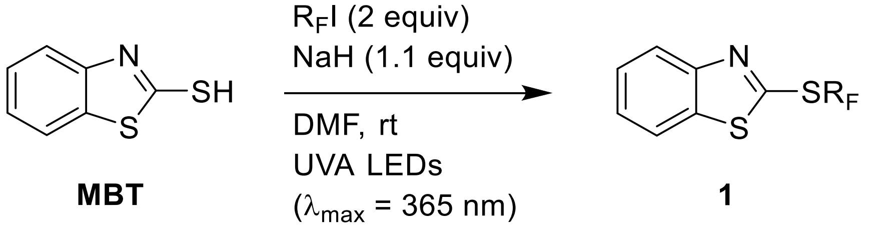

General Procedure A:[1] Mercaptobenzothiazole (1.0 equiv) was dissolved in DMF (0.16-0.25 M) under argon. Sodium hydride (60 wt % in mineral oil, 1.1 equiv) was added and the mixture was stirred at rt for 30 min. The perfluoroalkyl iodide (1.2 or 2 equiv) was then added (condensed in for I-C_2F_5) and the mixture was stirred under irradiation from UVA LEDS (λ_{max} = 365 nm) overnight. Water was added and the crude product was extracted with EtOAc (3×). The combined organic fractions were washed with water (3×), dried over anhydrous Na_2SO_4 and concentrated in vacuo. Purification by column chromatography over silica gel afforded the 2-((perfluoroalkyl)thio)benzo[*d*]thiazole intermediate **1**.

2-((Perfluoroethyl)thio)benzo[*d*]thiazole (1a)

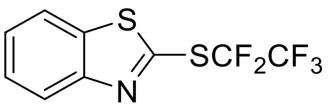

1a

Prepared using General Procedure A with 2 equiv of I-C_2F_5 on a 29.9 mmol scale. Pale yellow oil (5.21 g, 18.2 mmol, 61%).

^{1}H NMR (400 MHz, Chloroform-*d*) δ = 8.16 (dm, *J* = 8.1 Hz, 1H), 7.90 (dm, *J* = 8.1 Hz, 1H), 7.56 (ddd, *J* = 8.3, 7.2, 1.4 Hz, 1H), 7.50 (t, *J* = 8.5, 7.2, 1.3 Hz, 1H). **^{19}F NMR (376 MHz, Chloroform-*d*) δ** = -82.5 (t, *J* = 3 Hz, 3F), -90.1 (q, *J* = 3 Hz, 2F). **^{13}C NMR (151 MHz, Chloroform-*d*) δ** = 153.3 (C_q), 150.0 (C_q), 138.6 (C_q), 127.2 (CH), 127.0 (CH), 124.5 (CH), 121.4 (CH), 119.7 (tq, *J* = 294, 42 Hz, CF_3), 118.4 (qt, *J* = 287, 36 Hz, SCF_2). **HRMS (ESI):** m/z calculated for $[C_9H_5F_5NS_2]^+$ ($[M+H]^+$): 285.9778, measured: 285.9793. **IR (ATR):** ν (cm^{-1}): 3071, 2927, 1592, 1556, 1456, 1410, 1329, 1312, 1206, 1103, 1016, 994, 949, 853, 757, 751, 727, 708, 676, 651, 629, 608, 597, 550.

[1] Procedure adapted from: A. Harsányi, É. Dorkó, Á. Csapó, T. Bakó, C. Peltz, J. Rábai, *J. Fluorine Chem.* **2011**, *132*, 1241-1246.

2-((Perfluoropropyl)thio)benzo[*d*]thiazole (1b)

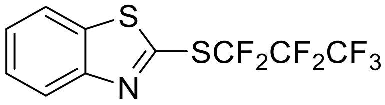

1b

Prepared using General Procedure A with 2 equiv of I-C_3F_7 on a 10.0 mmol scale. Pale yellow oil (2.64 g, 7.87 mmol, 79%).

^{1}H NMR (600 MHz, Chloroform-*d*) δ = 8.16 (dd, *J* = 8.2, 0.8 Hz, 1H), 7.90 (d, *J* = 8.2, 0.9 Hz, 1H), 7.55 (ddd, *J* = 8.3, 7.2, 1.3 Hz, 1H), 7.50 (ddd, *J* = 8.3, 7.2, 1.3 Hz, 1H). **^{19}F NMR (565 MHz, Chloroform-*d*) δ** = -79.9 (t, *J* = 9 Hz, 3F), -85.8 (q, *J* = 9 Hz, 2F), -123.3 (m, 2F). **^{13}C NMR (151 MHz, Chloroform-*d*) δ** = 153.3 (C_q), 149.9 (C_q), 138.6 (C_q), 127.2 (CH), 127.0 (CH), 124.5 (CH), 121.4 (CH), 122.1 (tt, *J* = 295, 34 Hz, SCF_2), 117.6 (qt, *J* = 288, 34 Hz, CF_3), 108.7 (tm, *J* = 266 Hz, CF_2). **HRMS (EI):** m/z calculated for $[C_{10}H_4F_7NS_2]^+$ ($[M]^+$): 334.9668, measured: 334.9644. **IR (ATR):** ν (cm^{-1}): 3068, 2927, 2854, 1556, 1456, 1410, 1335, 1313, 1208, 1185, 1115, 1091, 1040, 1016, 916, 846, 757, 741, 727, 708, 687, 674, 598.

2-((Perfluoropentyl)thio)benzo[*d*]thiazole (1c)

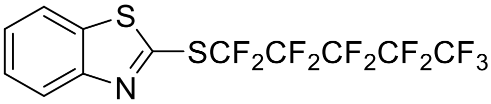

1c

Prepared using General Procedure A with 2 equiv of I-C_5F_{11} on a 6.0 mmol scale. Pale yellow oil (1.79 g, 4.11 mmol, 69%).

^{1}H NMR (600 MHz, Chloroform-*d*) δ = 8.16 (dm, *J* = 8.3 Hz, 1H), 7.90 (dm, *J* = 8.0 Hz, 1H), 7.56 (ddm, *J* = 8.3, 7.1 Hz, 1H), 7.50 (ddm, *J* = 8.1, 7.1 Hz, 1H). **^{19}F NMR (565 MHz, Chloroform-*d*) δ** = -80.8 (m, 3F), -84.9 (m, 2F), -119.0 (m, 2F), -122.2 (m, 2F), -126.1 (m, 2F). **^{13}C NMR (151 MHz, Chloroform-*d*) δ** = 153.3 (C_q), 150.0 (C_q), 138.7 (C_q), 127.2 (CH), 127.1 (CH), 124.5 (CH), 121.4 (CH), 122.8 (tt, *J* = 297, 35 Hz, SCF_2), 117.3 (qt, *J* = 286, 33 Hz, CF_3), 110.7 (tm, *J* = 270 Hz, CF_2) *Note: Two perfluoroalkyl ^{13}C peaks could not be observed.* **HRMS (EI):** m/z calculated for $[C_{12}H_4F_{11}NS_2]^+$ ($[M]^+$): 434.9604, measured: 434.9620. **IR (ATR):** ν (cm^{-1}): 3065, 3046, 1552, 1456, 1410, 1357, 1314, 1271, 1232, 1199, 1136, 1111, 1086, 1075, 1017, 999, 941, 854, 813, 767, 761, 745, 722, 696, 682, 659, 619, 594, 570.

2-((Perfluorooctyl)thio)benzo[*d*]thiazole (1d)[2]

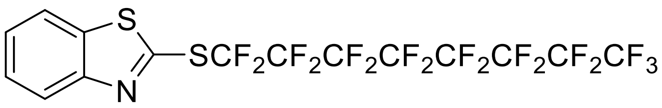

1d

Prepared using General Procedure A with 1.2 equiv of I-C_8F_{17} on a 3.0 mmol scale. White solid (1.67 g, 2,85 mmol, 95%).

^{1}H NMR (400 MHz, Chloroform-*d*) δ = 8.17 (dm, *J* = 8.3 Hz, 1H), 7.92 (dm, *J* = 8.1 Hz, 1H), 7.57 (m, 1H), 7.52 (m, 1H). **^{19}F NMR (376 MHz, Chloroform-*d*) δ** = -80.6 (t, *J* = 11 Hz, 3F), -84.8 (t, *J* = 15 Hz, 2F), -118.7 (m, 2F), -121.0 (m, 2F), -121.6 (m, 2F), -121.8 (m, 2F), -122.6 (m, 2F), -126.0 (m, 2F).

The data agree with literature precedents.[2]

2-((Perfluoropropan-2-yl)thio)benzo[*d*]thiazole (1e)

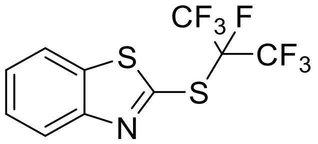

1e

Prepared using General Procedure A with 1.2 equiv of I-$CF(CF_3)_2$ on a 14.1 mmol scale. Pale yellow oil (4.30 g, 12.8 mmol, 91%).

^{1}H NMR (400 MHz, Chloroform-*d*) δ = 8.16 (dm, *J* = 8.2 Hz, 1H), 7.88 (dm, *J* = 7.9 Hz, 1H), 7.54 (ddd, *J* = 8.2, 7.3, 1.4 Hz, 1H), 7.48 (ddd, *J* = 8.0, 7.2, 1.3 Hz, 1H). **^{19}F NMR (376 MHz, Chloroform-*d*) δ** = -73.9 (d, *J* = 10 Hz, 6F), -155.6 (sept, *J* = 11 Hz, 1F). **^{13}C NMR (101 MHz, Chloroform-*d*) δ =** 153.2 (C_q), 149.9 (C_q), 138.8 (C_q), 127.1 (CH), 127.1 (CH, two overlapping peaks), 124.6 (CH), 121.4 (CH), 120.1 (qd, *J* = 288, 29 Hz, CF_3), 98.1 (d(sept), *J* = 255, 35 Hz, CF). **HRMS (EI):** m/z calculated for $[C_{10}H_4F_7NS_2]^+$ ($[M]^+$): 334.9668, measured: 334.9688. **IR (ATR):** ν (cm^{-1}): 3068, 2927, 2854, 1555, 1456, 1409, 1281, 1263, 1219, 1170, 1134, 1092, 992, 959, 934, 853, 755, 728, 717, 676, 619, 589, 602, 551.

[2] D. E. Yerien, S. Barata-Vallejo, B. Camps, A. E. Cristófalo, M. E. Cano, M. L. Uhrig, A. Postigo, *Catal. Sci. Technol.* **2017**, *7*, 2274-2282.

2.2 *N*-Methylation of 2-((Perfluoroalkyl)thio)benzo[*d*]thiazoles 1

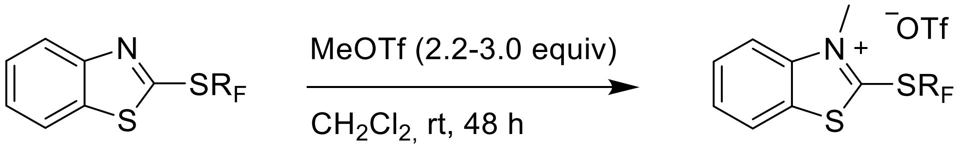

1 **BT-SR$_F$**

General Procedure B: 2-Substituted benzothiazoles (1.0 equiv) were dissolved in dry CH_2Cl_2 (0.10 M) and methyl trifluoromethanesulfonate (2.2-3.0 equiv) was added. The reaction mixture was stirred at rt for 48 h and the product was precipitated with diethyl ether. The suspension was then filtered, and the residue washed with diethyl ether (3×). After drying in vacuo, **BT-SR$_F$** salts were obtained as off-white solids.

3-Methyl-2-((perfluoroethyl)thio)benzo[*d*]thiazol-3-ium trifluoromethanesulfonate (BT-SC$_2$F$_5$)

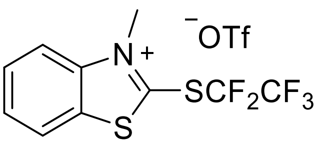

BT-SC$_2$F$_5$

Prepared from **1a** using General Procedure B with 2.2 equiv of MeOTf on a 16.3 mmol scale. Off-white solid (6.70 g, 14,9 mmol, 91%).

^{1}H NMR (400 MHz, Acetonitrile-*d*$_3$) δ = 8.40 (dm, J = 8.3 Hz, 1H), 8.29 (dm, J = 8.8 Hz, 1H), 8.07 (ddm, J = 8.6, 7.2 Hz, 1H), 7.99 (ddm, J = 8.4, 7.2 Hz, 1H), 4.47 (s, 3H). **^{19}F NMR (565 MHz, Acetonitrile-*d*$_3$) δ** = -78.5 (3F), -82.0 (t, J = 3 Hz, 3F), -87.8 (q, J = 3 Hz, 2F). **^{13}C NMR (151 MHz, Acetonitrile-*d*$_3$) δ** = 158.6 (C_q), 143.9 (C_q), 134.6 (C_q), 132.5 (CH), 131.7 (CH), 125.3 (CH), 122.0 (q, J = 322 Hz, SO_2CF_3), 119.9 (tq, J = 299 Hz, 42, CF_3), 119.6 (CH), 118.7 (qt, J = 286 Hz, 35, SCF_2), 40.2 (CH_3). **HRMS (ESI):** m/z calculated for $[C_{10}H_7F_5NS_2]^+$ ($[M-OTf]^+$): 299.9935, measured: 299.9948. **IR (ATR):** ν (cm^{-1}): 3092, 3028, 1578, 1491, 1463, 1433, 1383, 1330, 1278, 1267, 1241, 1211, 1160, 1337, 1111, 1207, 955, 815, 160, 150, 722, 714, 658, 638, 602, 573.

3-Methyl-2-((perfluoropropyl)thio)benzo[*d*]thiazol-3-ium trifluoromethanesulfonate (BT-SC$_3$F$_7$)

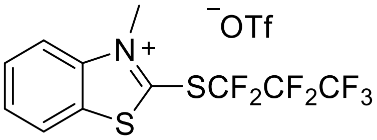

BT-SC$_3$F$_7$

Prepared from **1b** using General Procedure B with 3.0 equiv of MeOTf on a 5.25 mmol scale. Off-white solid (2.46 g, 4.92 mmol, 94%).

^{1}H NMR (400 MHz, Acetonitrile-*d*$_3$) δ = 8.39 (d, *J* = 8.5 Hz, 1H), 8.27 (d, *J* = 8.8 Hz, 1H), 8.04 (t, *J* = 8.0 Hz, 1H), 7.96 (t, *J* = 7.8 Hz, 1H), 4.45 (s, 3H). **^{19}F NMR (376 MHz, Acetonitrile-*d*$_3$) δ** = -79.2 (3F), -80.5 (t, *J* = 9 Hz, 3F), -83.8 (q, *J* = 9 Hz, 2F), -123.2 (m, 2F). **^{13}C NMR (176 MHz, Acetonitrile-*d*$_3$) δ** = 158.7 (C_q), 144.1 (C_q), 134.7 (C_q), 132.6 (CH), 131.8 (CH), 125.3 (CH), 122.5 (tt, *J* = 299, 35 Hz, SCF_2), 122.0 (q, *J* = 320 Hz, SO_2CF_3), 118.3 (qt, *J* = 288, 34 Hz, CF_3), 119.7 (CH), 109.4 (tm, *J* = 267 Hz, CF_2), 40.3 (CH_3). **HRMS (ESI):** m/z calculated for $[C_{11}H_7F_7NS_2]^+$ ($[M-OTf]^+$): 349.9903, measured: 349.9922. **IR (ATR):** ν (cm^{-1}): 3089, 3031, 1578, 1490, 1463, 1433, 1382, 1340, 1277, 1239, 1266, 1218, 1189, 1163, 1163, 1121, 1054, 1027, 914, 848, 815, 761, 751, 743, 723, 714, 687, 639, 574.

3-Methyl-2-((perfluoropentyl)thio)benzo[*d*]thiazol-3-ium trifluoromethanesulfonate (BT-SC_5F_{11})

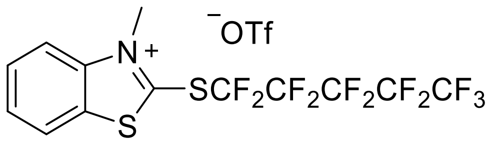

BT-SC_5F_{11}

Prepared from **1c** using General Procedure B with 2.5 equiv of MeOTf on a 3.68 mmol scale. Off-white solid (2.07 g, 3.45 mmol, 94%).

^{1}H NMR (400 MHz, Acetonitrile-*d*$_3$) δ = 8.42 (d, *J* = 8.3 Hz, 1H), 8.31 (d, *J* = 8.6 Hz, 1H), 8.07 (t, *J* = 8.0 Hz, 1H), 8.00 (t, *J* = 7.8 Hz, 1H), 4.48 (s, 3H). **^{19}F NMR (376 MHz, Acetonitrile-*d*$_3$) δ** = -79.2 (3F), -81.4 (t, *J* = 10 Hz, 3F), -82.7 (t, *J* = 14 Hz, 2F), -118.8 (m, 2F), -122.5 (m, 2F), -126.5 (m, 2F). **^{13}C NMR (151 MHz, Acetonitrile-*d*$_3$) δ** = 159.6 (C_q), 144.0 (C_q), 134.7 (C_q), 132.6 (CH), 131.8 (CH), 125.3 (CH), 123.1 (tt, *J* = 300, 35 Hz, SCF_2), 122.0 (q, *J* = 320 Hz, SO_2CF_3), 119.6 (CH), 118.0 (qt, *J* = 289, 33 Hz, CF_3), 111.3 (tm, *J* = 267 Hz, CF_2), 40.2 (CH_3) *Note: Two perfluoroalkyl ^{13}C peaks could not be observed.* **HRMS (ESI):** m/z calculated for $[C_{13}H_7F_{11}NS_2]^+$ ($[M-OTf]^+$): 449.9839, measured: 449.9843. **IR (ATR):** ν (cm^{-1}): 3101, 3031, 1577, 1490, 1462, 1433, 1357, 1277, 1266, 1241, 1224, 1201, 1163, 1142, 1081, 1028, 941, 863, 847, 815, 762, 737, 722, 697, 664, 639, 597, 572.

3-Methyl-2-((perfluorooctyl)thio)benzo[d]thiazol-3-ium trifluoromethanesulfonate (BT-SC_8F_{17})

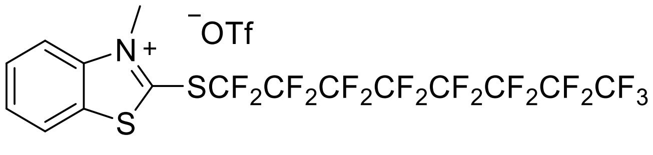

BT-SC_8F_{17}

Prepared from **1d** using General Procedure B with 3.0 equiv of MeOTf on a 1.57 mmol scale. Off-white solid (1.08 g, 1.44 mmol, 92%).

^{1}H NMR (400 MHz, Acetonitrile-*d*$_3$) δ = 8.40 (d, *J* = 8.4 Hz, 1H), 8.29 (d, *J* = 8.7 Hz, 1H), 8.07 (t, *J* = 7.6 Hz, 1H), 7.99 (t, *J* = 7.8 Hz, 1H), 4.47 (s, 3H). **^{19}F NMR (376 MHz, Acetonitrile-*d*$_3$) δ** = -79.3 (3F), -81.4 (t, *J* = 10 Hz, 3F), -82.5 (t, *J* = 14 Hz, 2F), -118.5 (m, 2F), -121.4 (m, 2F), -121.5 (m, 2F), -122.2 (m, 2F), -123.0 (m, 2F), -126.5 (m, 2F). **^{13}C NMR (176 MHz, Acetonitrile-*d*$_3$) δ** = 158.5 (C_q), 143.9 (C_q), 134.7

(C_q), 132.6 (CH), 131.8 (CH), 125.3 (CH), 123.1 (tt, J = 300, 35 Hz, SCF_2), 122.0 (q, J = 318 Hz, SO_2CF_3), 119.6 (CH), 118.0 (qt, J = 288, 32 Hz, CF_3), 111.6 (m, several overlapping peaks, CF_2), 40.2 (CH_3) *Note: Several perfluoroalkyl ^{13}C peaks could not be assigned.* **HRMS (ESI):** m/z calculated for $[C_{16}H_7F_{17}NS_2]^+$ ($[M\text{-}OTf]^+$): 599.9743, measured: 599.9730. **IR (ATR):** ν (cm^{-1}): 3101, 2361, 1576, 1490, 1461, 1435, 1370, 1328, 1281, 1251, 1200, 1149, 1134, 1097, 1054, 1031, 958, 930, 849, 816, 798, 768, 756, 741, 723, 714, 707, 655.

3-Methyl-2-((perfluoroisopropyl)thio)benzo[*d*]thiazol-3-ium trifluoromethanesulfonate (BT-SCF(CF_3)$_2$)

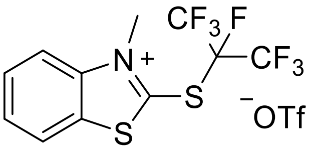

BT-SCF(CF_3)$_2$

Prepared from **1e** using General Procedure B with 3.0 equiv of MeOTf on a 12.8 mmol scale. Off-white solid (3.73 g, 10.7 mmol, 83%).

^{1}H NMR (700 MHz, Acetonitrile-*d$_3$*) δ = 8.43 (ddd, J = 8.4, 1.2, 0.7 Hz, 1H), 8.33 (dt, J = 8.7, 0.9 Hz, 1H), 8.07 (ddd, J = 8.6, 7.2, 1.2 Hz, 1H), 7.99 (ddd, J = 8.3, 7.2, 1.0 Hz, 1H), 4.51 (s, 3H). **^{19}F NMR (376 MHz, Acetone-*d$_6$*) δ** = -74.7 (d, J = 11 Hz, 6F), -78.9 (3F), -154.7 (sept, J = 11 Hz, 1F). **^{13}C NMR (177 MHz, Acetonitrile-*d$_3$*) δ** = 158.3 (C_q), 143.8 (C_q), 134.8 (C_q), 132.7 (CH), 132.0 (CH), 125.4 (CH), 122.0 (q, J = 321 Hz, SO_2CF_3), 120.4 (qd, J = 289, 28 Hz, CF_3), 119.7 (CH), 98.4 (d(sept), J = 260, 36 Hz, CF), 40.4 (CH_3). **HRMS (ESI):** m/z calculated for $[C_{11}H_7F_7NS_2]^+$ ($[M\text{-}OTf]^+$): 349.9903, measured: 349.9923. **IR (ATR):** ν (cm^{-1}): 3086, 3031, 1579, 1491, 1463, 1432, 1382, 1276, 1240, 1221, 1178, 1159, 1139, 1099, 1027, 965, 937, 814, 755, 720, 638, 572, 540.

3 Scope and Limitations of the Deoxyperfluoroalkylthiolation of Alcohols

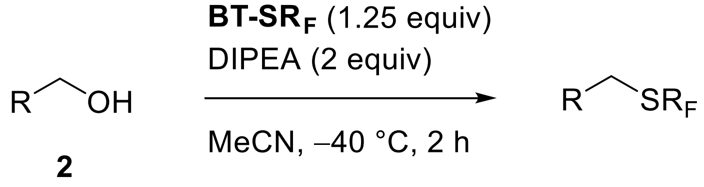

General Procedure C: The alcohol (0.50 mmol, 1.0 equiv) was dissolved in MeCN (0.17 M), **BT-SR$_F$** (0.625 mmol, 1.25 equiv) was added and the reaction mixture was cooled to −40 °C. NEt(iPr)$_2$ (174 µL,1.0 mmol, 2.0 equiv) was then added dropwise and the reaction mixture was stirred for 1-2 h at −40 °C. The reaction mixture was concentrated in vacuo and purified by column chromatography over silica gel.

3.1 Deoxyperfluoroalkylation of 4-Nitrobenzyl alcohol (2a) with Different BT-SR$_F$ Reagents

3.1.1 With BT-SC$_2$F$_5$

a) At rt (DIPEA added at 0 °C then reaction warmed to rt over 2 h)

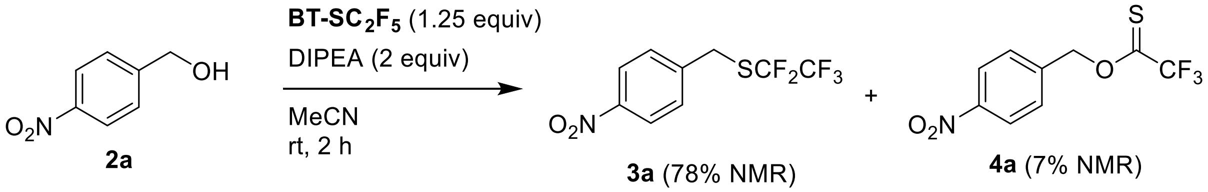

Crude ^{1}H NMR Spectrum: 0.5 mmol scale, CH$_2$Br$_2$ (0.1 mmol) as internal reference

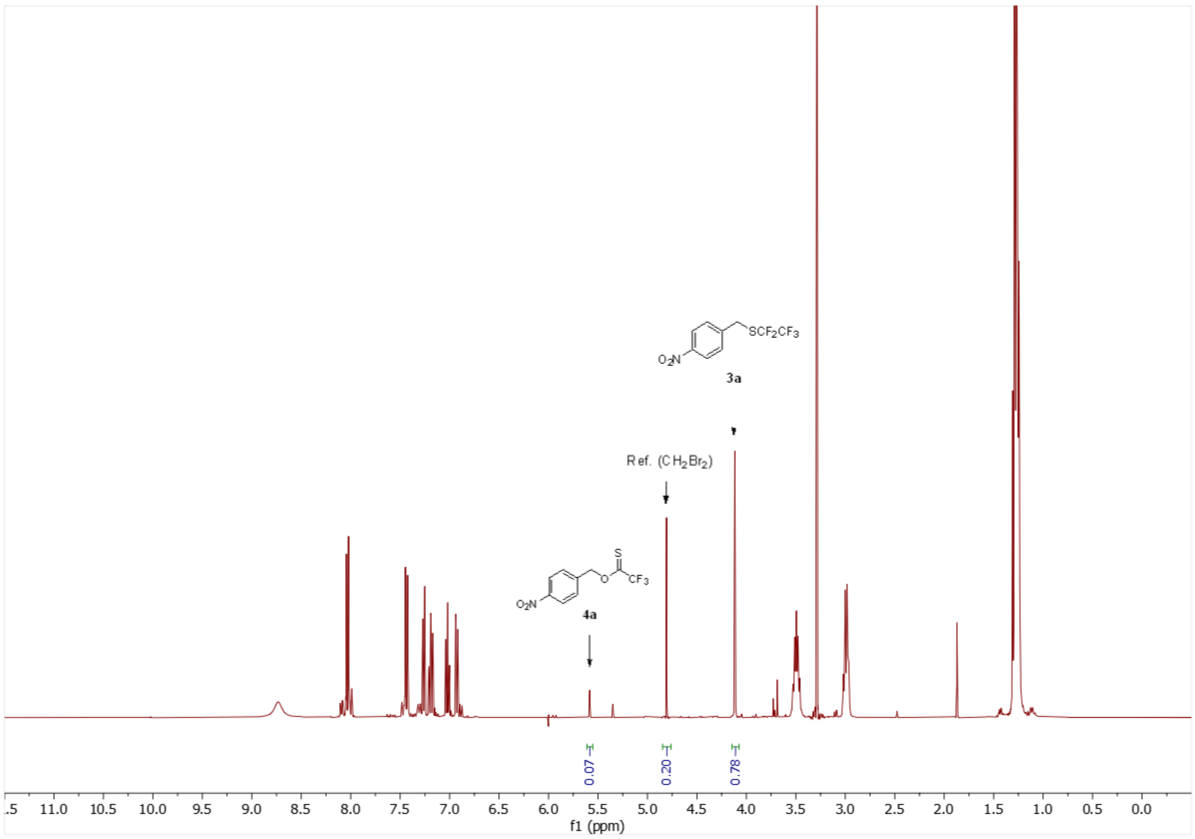

b) At −40 °C (General Procedure C)

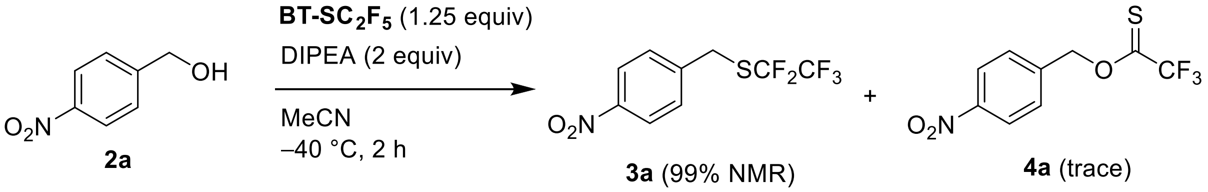

Crude ^{1}H NMR Spectrum: 0.5 mmol scale, CH_2Br_2 (0.1 mmol) as internal reference

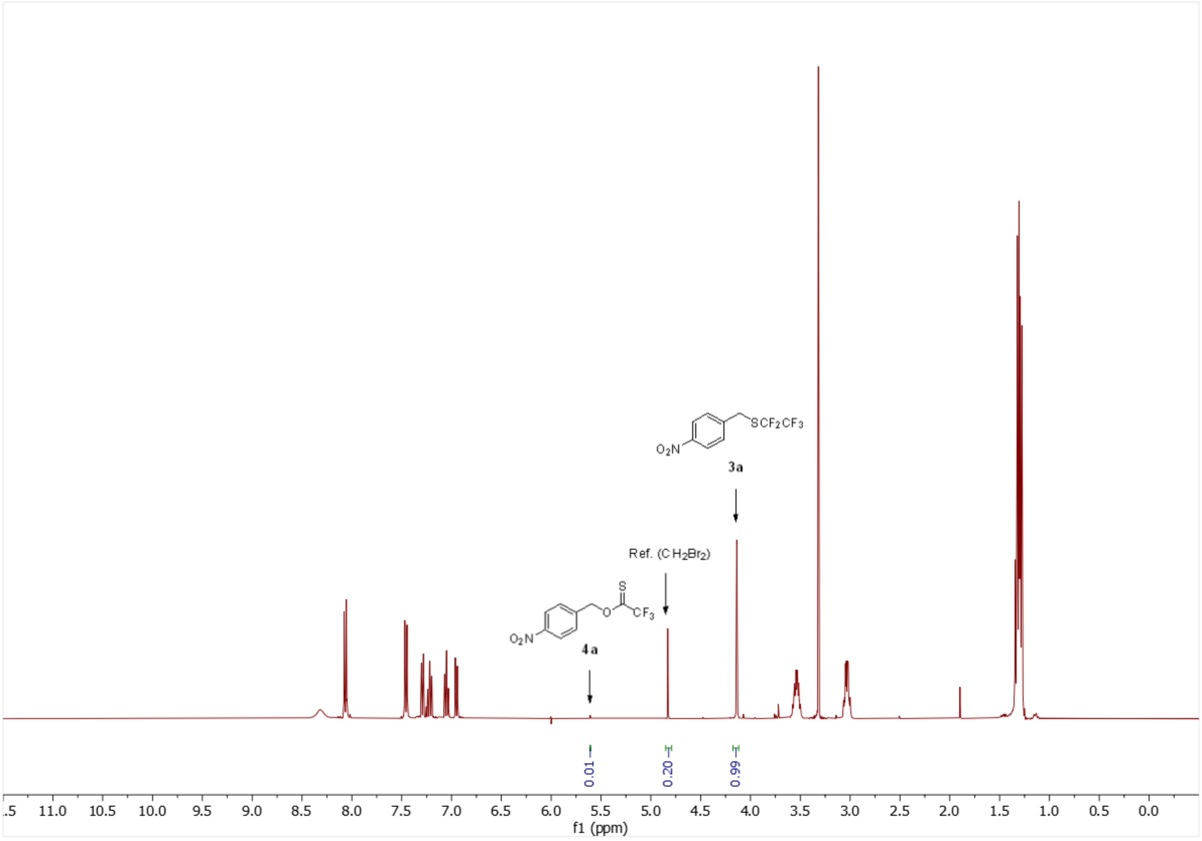

(4-Nitrobenzyl)(perfluoroethyl)sulfane (3a)

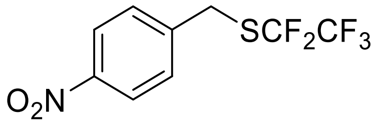

3a

Prepared from 4-nitrobenzyl alcohol (**2a**) and BT-SC_2F_5 using General Procedure C. Yellow liquid (131 mg, 0.46 mmol, 91%).

^{1}H NMR (600 MHz, Chloroform-*d*) δ = 8.20 (d, *J* = 8.6 Hz, 2H), 7.53 (d, *J* = 8.6 Hz, 2H), 4.21 (s, 2H). **^{19}F NMR (565 MHz, Chloroform-*d*) δ** = -83.3 (m, 3F), -92.0 (m, 2F). **^{13}C NMR (151 MHz, Chloroform-*d*) δ** = 147.8 (C_q), 142.7 (C_q), 130.0 (CH), 124.2 (CH), 121.3 (tq, *J* = 289, 41 Hz, CF_2), 118.5 (qt, *J* = 286, 36 Hz, CF_3), 32.2 (t, *J* = 4 Hz, CH_2). **HRMS (ESI)**: m/z calculated for $[C_9H_5F_5NO_2S]^-$ ($[M-H]^-$): 285.9967, measured: 285.9967. **IR (ATR):** ν (cm^{-1}): 3117, 3093, 2952, 2860, 1602, 1521, 1346, 1323, 1254, 1205, 1095, 1016, 966, 889, 858, 819, 802, 750, 706, 642, 624, 590, 551.

3.1.2 With BT-SC_3F_7

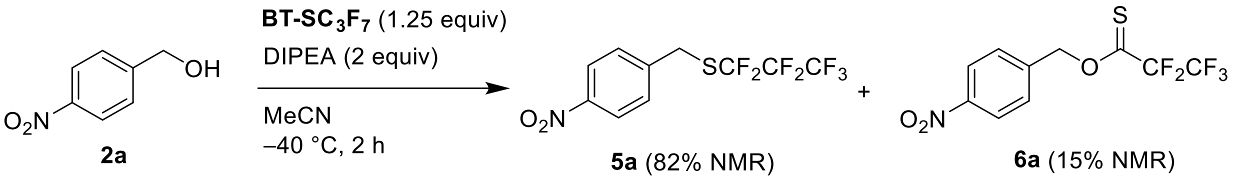

Crude ^{1}H NMR Spectrum: 0.5 mmol scale, CH_2Br_2 (0.5 mmol) as internal reference

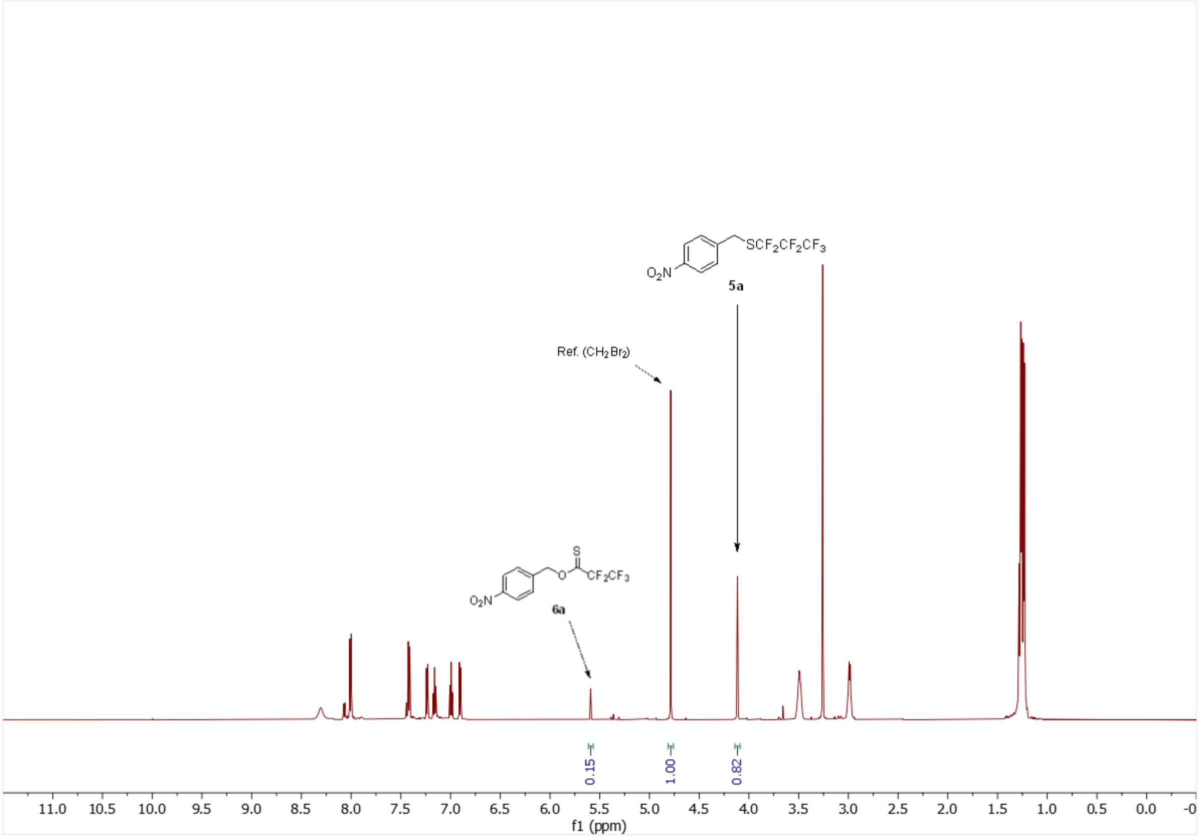

(4-Nitrobenzyl)(perfluoropropyl)sulfane (5a)

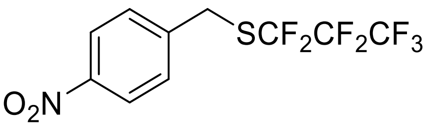

5a

Prepared from 4-nitrobenzyl alcohol (**2a**) and BT-SC_3F_7 using General Procedure C. Yellow solid (139 mg, 0.412 mmol, 82%).

^{1}H NMR (400 MHz, Chloroform-*d*) δ = 8.21 (dm, *J* = 8.7 Hz, 2H), 7.55 (dm, *J* = 8.7 Hz, 2H), 4.24 (s, 2H). **^{19}F NMR (377 MHz, Chloroform-*d*) δ** = -80.0 (t, *J* = 10 Hz, 3F), -88.0 (qt, *J* = 9, 4 Hz, 2F), -124.0 (t, *J* = 4 Hz, 2F). **^{13}C NMR (177 MHz, Chloroform-*d*) δ** = 147.8 (C_q), 142.8 (C_q), 130.1 (CH), 124.3 (CH), 123.8 (tt, *J* = 290, 34 Hz, SCF_2), 117.7 (qt, *J* = 288, 34 Hz, CF_3), 108.6 (tm, *J* = 265 Hz, CF_2), 32.3 (t, *J* = 4 Hz, CH_2). **HRMS (EI)**: m/z calculated for $[C_{10}H_6F_7NO_2S]^+$ ($[M]^+$): 337.0002, measured: 337.0004. **IR (ATR):** ν (cm^{-1}): 3083, 2929, 2857, 1931, 1726, 1602, 1522, 1494, 1424, 1346, 1208, 1182, 1109, 1087, 1038, 1017, 926, 890, 855, 801, 751, 742, 717, 706, 678, 653, 631, 622, 606.

3.1.3 With BT-SC_5F_{11}

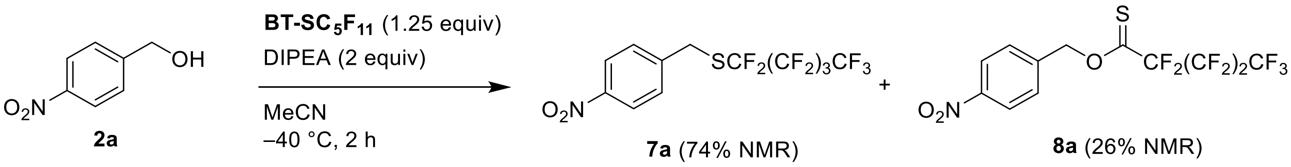

Crude ^{1}H NMR Spectrum: 0.5 mmol scale, CH_2Br_2 (0.5 mmol) as internal reference

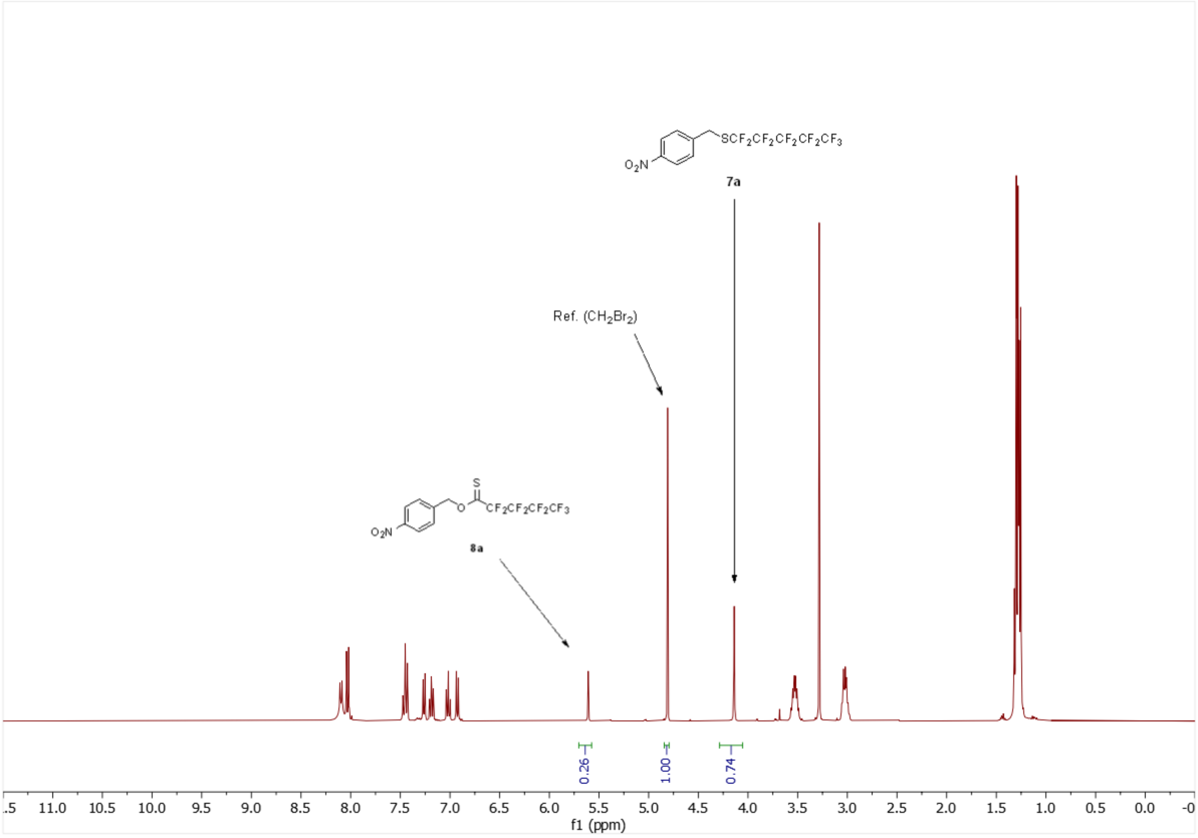

3.1.4 With BT-SC_8F_{17}

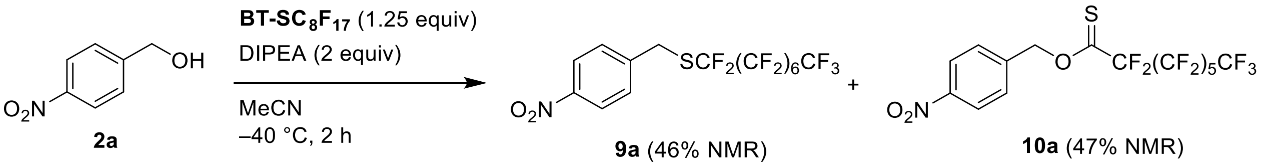

Crude ^{1}H NMR Spectrum: 0.5 mmol scale, CH_2Br_2 (0.5 mmol) as internal reference

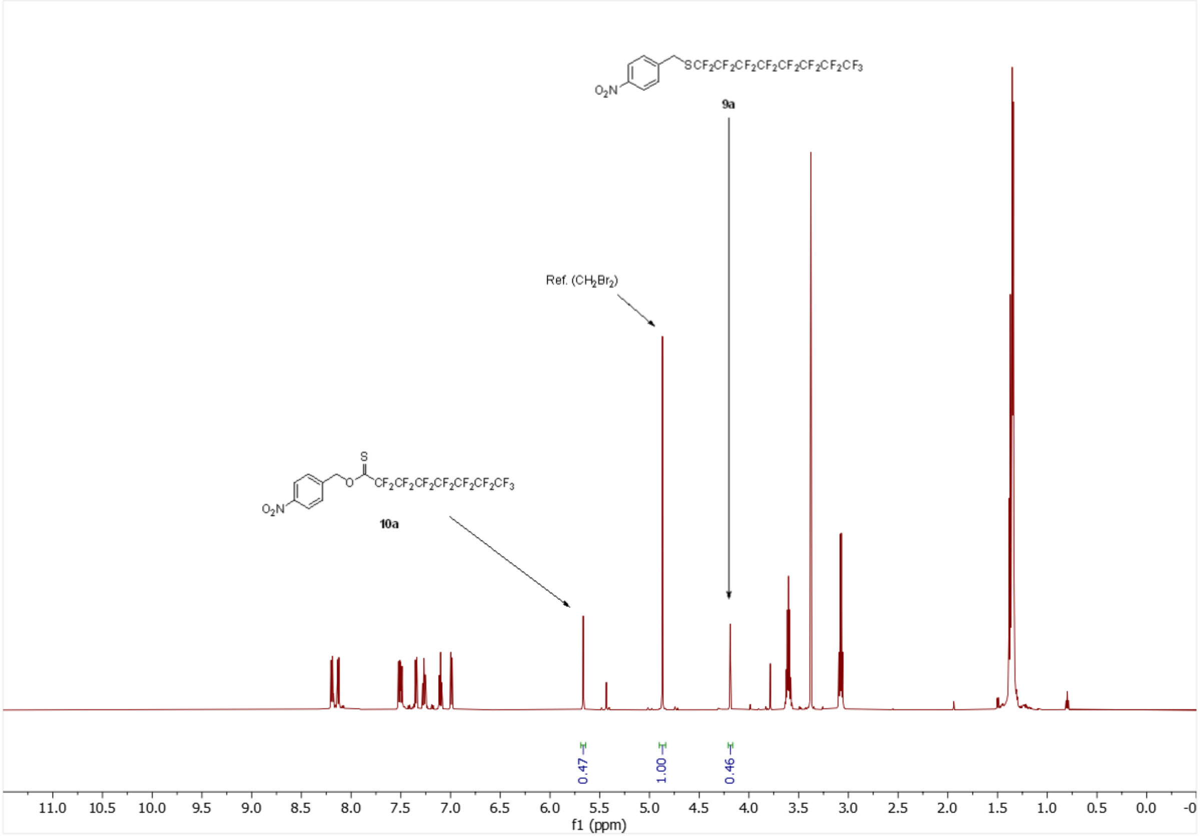

3.1.5 With BT-SCF(CF_3)$_2$

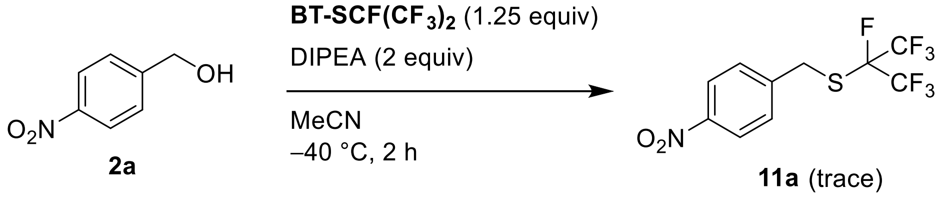

2a

11a (trace)

Crude ^{1}H NMR Spectrum: 0.5 mmol scale, CH_2Br_2 (0.5 mmol) as internal reference

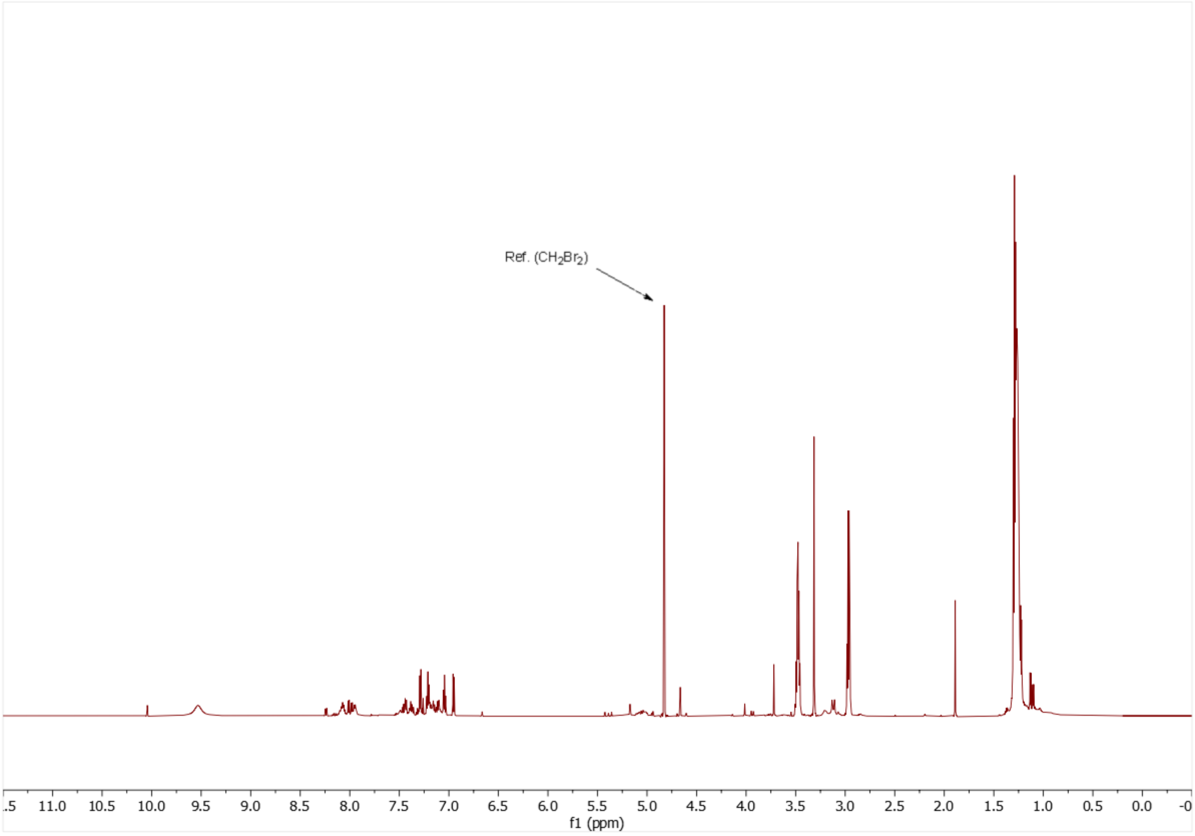

3.2 Deoxypentafluoroethylation of Alcohols 2 with BT-SC_2F_5

(Benzyl)(perfluoroethyl)sulfane (3b)[3]

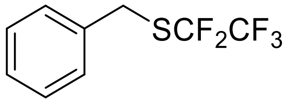

3b

Prepared from benzyl alcohol (**2b**) and BT-SC_2F_5 using General Procedure C. Pale yellow liquid (88 mg, 0.36 mmol, 73%).

^{1}H NMR (400 MHz, Chloroform-*d*) δ = 7.29 – 7.38 (m, 5H), 4.17 (s, 2H). **^{19}F NMR (377 MHz, Chloroform-*d*) δ** = -83.3 (t, *J* = 4 Hz, 3F), -92.3 (t, *J* = 4 Hz, 2F).

The data agree with literature precedents.[3]

(4-Iodobenzyl)(perfluoroethyl)sulfane (3c)

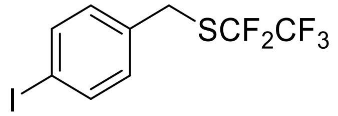

3c

Prepared from 4-iodobenzyl alcohol (**2c**) and BT-SC_2F_5 using General Procedure C. Yellow liquid (140 mg, 0.38 mmol, 76%).

^{1}H NMR (400 MHz, Chloroform-*d*) δ = 7.69 (dm, *J* = 8.5 Hz, 2H), 7.10 (dm, *J* = 8.5 Hz, 2H), 7.30 (m, 1H), 4.09 (s, 2H). **^{19}F NMR (376 MHz, Chloroform-*d*) δ** = -83.2 (t, *J* = 4 Hz, 3F), -92.2 (q, *J* = 4 Hz, 2F). **^{13}C NMR (101 MHz, Chloroform-*d*) δ** = 138.2 (CH), 134.6 (C_q), 131.0 (CH), 121.4 (tq, *J* = 288, 41 Hz, CF_2), 118.7 (qt, *J* = 286, 37 Hz, CF_3), 92.8 (C_q), 32.6 (t, *J* = 4 Hz, CH_2). **HRMS (EI)**: m/z calculated for $[C_9H_6F_5IS]^+$ ($[M]^+$): 367.9150, measured: 367.9176. **IR (ATR):** ν (cm^{-1}): 3040, 2946, 1906, 1588, 1485, 1400, 1322, 1205, 1094, 1060, 1008, 966, 878, 827, 809, 750, 736, 676, 642, 624, 598, 551.

---

3 Q. Glenadel, M. Bordy, S. Alazet, A. Tlili, T. Billard, *Asian J. Org. Chem.* **2016**, *5*, 428-433.

(Perfluoroethyl)(2,4,6-trichlorobenzyl)sulfane (3d)

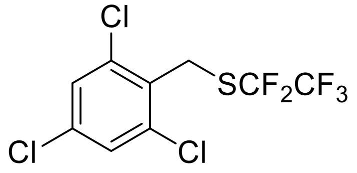

3d

Prepared from 2,4,6-trichlorobenzyl alcohol (**2d**) and BT-SC_2F_5 using General Procedure C. Colourless liquid (166 mg, 0.48 mmol, 96%).

^{1}H NMR (400 MHz, Chloroform-*d*) δ = 7.37 (s, 2H), 4.44 (s, 2H). **^{19}F NMR (376 MHz, Chloroform-*d*) δ** = -83.1 (t, *J* = 4 Hz, 3F), -92.2 (q, *J* = 4 Hz, 2F). **^{13}C NMR (101 MHz, Chloroform-*d*) δ** = 136.6 (C_q), 135.3 (C_q), 130.1 (C_q), 128.8 (CH), 121.5 (tq, *J* = 288, 41 Hz, CF_2), 118.7 (qt, *J* = 286, 37 Hz, CF_3), 28.2 (t, *J* = 4 Hz, CH_2). **HRMS (EI)**: m/z calculated for $[C_9H_4Cl_3F_5S]^+$ ($[M]^+$): 343.9014, measured: 343.9037. **IR (ATR):** ν (cm^{-1}): 3089, 2933, 2857, 1726, 1581, 1550, 1441, 1420, 1376, 1321, 1254, 1208, 1135, 1094, 967, 897, 857, 786, 750, 679, 656, 640, 625, 590, 563, 551.

([1,1'-Biphenyl]-4-ylmethyl)(perfluoroethyl)sulfane (3e)

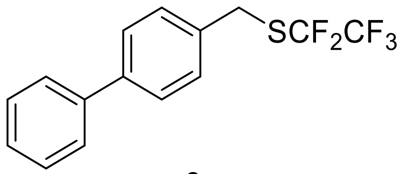

3e

Prepared from biphenyl-4-methanol (**2e**) and BT-SC_2F_5 using General Procedure C. Colourless solid (144 mg, 0.45 mmol, 90%).

^{1}H NMR (400 MHz, Chloroform-*d*) δ = 7.68 – 7.59 (m, 4H), 7.56 – 7.37 (m, 5H), 4.25 (s, 2H). **^{19}F NMR (376 MHz, Chloroform-*d*) δ** = -83.2 (t, *J* = 4 Hz, 3F), -92.1 (q, *J* = 4 Hz, 2F). **^{13}C NMR (101 MHz, Chloroform-*d*) δ** = 141.3 (C_q), 140.5 (C_q), 133.7 (C_q), 129.7 (CH), 129.0 (CH), 127.8 (CH), 127.7 (CH), 127.2 (CH), 121.6 (tq, *J* = 288, 41 Hz, CF_2), 118.8 (qt, *J* = 286, 37 Hz, CF_3), 32.8 (t, *J* = 4 Hz, CH_2). **HRMS (EI)**: m/z calculated for $[C_{15}H_{11}F_5S]^+$ ($[M]^+$): 318.0496, measured: 318.0509. **IR (ATR):** ν (cm^{-1}): 3083, 3033, 2939, 2860, 1977, 1919, 1796, 1683, 1598, 1565, 1522, 1488, 1443, 1408, 1324, 1248, 1199, 1129, 1096, 1007, 966, 843, 751, 769, 735, 714, 690, 638, 625, 593, 540.

((4-*tert*-Butyl)benzyl)(perfluoroethyl)sulfane (3f)

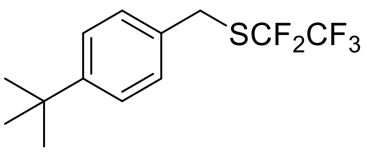

3f

Prepared from 4-*tert*-butylbenzyl alcohol (**2f**) and BT-SC_2F_5 using General Procedure C. Yellow liquid (119 mg, 0.40 mmol, 80%).

^{1}H NMR (600 MHz, Chloroform-*d*) δ = 7.39 (d, *J* = 8.3 Hz, 2H), 7.30 (d, *J* = 8.1 Hz, 2H), 4.15 (s, 2H), 1.33 (s, 9H). **^{19}F NMR (565 MHz, Chloroform-*d*) δ** = -83.2 (t, *J* = 5 Hz, 3F), -92.3 (q, *J* = 5 Hz, 2F). **^{13}C NMR (151 MHz, Chloroform-*d*) δ** = 151.4 (C_q), 131.4 (C_q), 128.9 (CH), 126.0 (CH), 123.4 (tq, *J* = 288, 40 Hz, CF_2), 118.7 (qt, *J* = 286, 36 Hz, CF_3), 34.7 (C_q), 32.6 (t, *J* = 4 Hz, CH_2), 31.3 (CH_3). **HRMS (EI)**: m/z calculated for $[C_{13}H_{15}F_5S]^+$ ($[M]^+$): 298.0815, measured: 298.0840. **IR (ATR):** ν (cm^{-1}): 3028, 2965, 2909, 2869, 1517, 1465, 1414, 1365, 1322, 1269, 1252, 1209, 1095, 1019, 986, 836, 750, 705, 624, 590, 559.

Methyl 4-(((perfluoroethyl)thio)methyl)benzoate (3g)

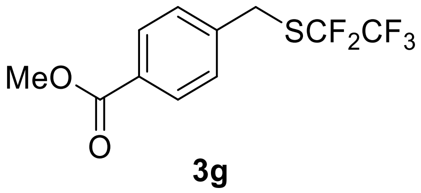

3g

Prepared from methyl 4-(hydroxymethyl)benzoate (**2g**) and BT-SC_2F_5 using General Procedure C. Yellow liquid (128 mg, 0.43 mmol, 85%).

^{1}H NMR (600 MHz, Chloroform-*d*) δ = 8.02 (d, *J* = 8.3 Hz, 2H), 7.42 (d, *J* = 8.3 Hz, 2H), 4.17 (s, 2H), 3.91 (s, 3H). **^{19}F NMR (565 MHz, Chloroform-*d*) δ** = -83.3 (m, 3F), -92.2 (m, 2F). **^{13}C NMR (151 MHz, Chloroform-*d*) δ** = 167.3 (C_q), 138.8 (C_q), 130.3 (CH), 130.0 (C_q), 129.1 (CH), 120.5 (tq, *J* = 289, 41 Hz, CF_2), 118.6 (qt, *J* = 286, 36 Hz, CF_3), 52.3 (CH_3), 32.6 (t, *J* = 4 Hz, CH_2). **HRMS (EI)**: m/z calculated for $[C_{11}H_9F_5O_2S]^+$ ($[M]^+$): 300.0243, measured: 300.0254. **IR (ATR):** ν (cm^{-1}): 3007, 2956, 2848, 1720, 1613, 1578, 1437, 1415, 1323, 1279, 1206, 1180, 1095, 1021, 966, 861, 839, 797, 775, 750, 723, 711, 624, 590, 552.

(3-Bromobenzyl)(perfluoroethyl)sulfane (3h)

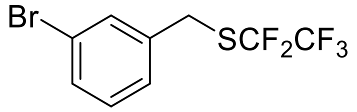

3h

Prepared from methyl 3-bromobenzyl alcohol (**2h**) and BT-SC_2F_5 using General Procedure C. Pale red liquid (126 mg, 0.39 mmol, 78%).

^{1}H NMR (400 MHz, Chloroform-*d*) δ = 7.52 (t, *J* = 1.8 Hz, 1H), 7.45 (ddd, *J* = 7.8, 2.0, 1.2 Hz, 1H), 7.30 (m, 1H), 7.23 (t, *J* = 7.8 Hz, 1H), 4.11 (s, 2H). **^{19}F NMR (376 MHz, Chloroform-*d*) δ** = -83.3 (t, *J* = 4 Hz, 3F), -92.2 (q, *J* = 4 Hz, 2F). **^{13}C NMR (101 MHz, Chloroform-*d*) δ** = 137.2 (C_q), 132.2 (CH), 131.5 (CH), 130.6 (CH), 127.4 (CH), 122.9 (C_q), 121.4 (tq, *J* = 288, 41 Hz, CF_2), 118.7 (qt, *J* = 286, 37 Hz, CF_3), 32.4 (t, *J* = 4 Hz, CH_2). **HRMS (EI)**: m/z calculated for $[C_9H_6BrF_5S]^+$ ($[M]^+$): 319.9288, measured: 319.9280. **IR (ATR):** ν (cm^{-1}): 3071, 2949, 1601, 1571, 1476, 1429, 1322, 1206, 1095, 1072, 998, 966, 888, 848, 787, 750, 730, 706, 681, 668, 625, 591, 577, 550.

(Perfluoroethyl)(3-phenylprop-2-yn-1-yl)sulfane (3i)

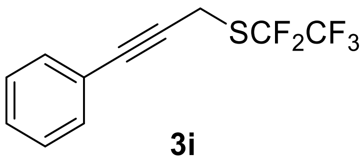

3i

Prepared from 3-phenylprop-2-yn-1-ol (**2i**) and BT-SC_2F_5 using General Procedure C. Yellow liquid (108 mg, 0.41 mmol, 81%).

^{1}H NMR (600 MHz, Chloroform-*d*) δ = 7.44 (dd, *J* = 7.7, 1.8 Hz, 2H), 7.37 – 7.30 (m, 3H), 7.30 (m, 1H), 3.94 (s, 2H). **^{19}F NMR (565 MHz, Chloroform-*d*) δ** = -83.3 (t, *J* = 4 Hz, 3F), -93.2 (q, *J* = 4 Hz, 2F). **^{13}C NMR (151 MHz, Chloroform-*d*) δ** = 131.9 (CH), 128.9 (CH), 128.5 (CH), 122.3 (C_q), 121.3 (tq, *J* = 289, 41 Hz, CF_2), 118.7 (qt, *J* = 286, 36 Hz, CF_3), 84.9 (C_q), 82.2 (C_q), 18.5 (t, *J* = 6 Hz, CH_2). **HRMS (EI)**: m/z calculated for $[C_{11}H_7F_5S]^+$ ($[M]^+$): 266.0183, measured: 266.0193. **IR (ATR):** ν (cm^{-1}): 3059, 2928, 2854, 2224, 1729, 1599, 1492, 1443, 1410, 1312, 1271, 1207, 1131, 1095, 1029, 964, 916, 860, 751, 726, 689, 640 625, 551.

(Cinnamyl)(perfluoroethyl)sulfane (3j)[3]

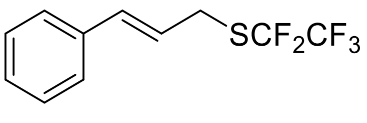

3j

Prepared from cinnamyl alcohol (**2j**) and BT-SC_2F_5 using General Procedure C. Pale yellow liquid (17 mg, 0.063 mmol, 13%).

^{1}H NMR (400 MHz, Chloroform-*d*) δ = 7.30 – 7.48 (m, 4H), 7.28 (m, 1H), 6.63 (d, *J* = 15.5 Hz, 1H), 6.22 (dt, *J* = 15.5, 7.4 Hz, 1H), 3.77 (d, *J* = 7.4 Hz, 2H). **^{19}F NMR (377 MHz, Chloroform-*d*) δ** = -83.3 (t, *J* = 4 Hz, 3F), -91.9 (t, *J* = 4 Hz, 2F).

The data agree with literature precedents.[3]

(Perfluoroethyl)(3-phenylpropyl)sulfane (3k) + *O*-(3-Phenylpropyl) 2,2,2-trifluoroethanethioate (4k)

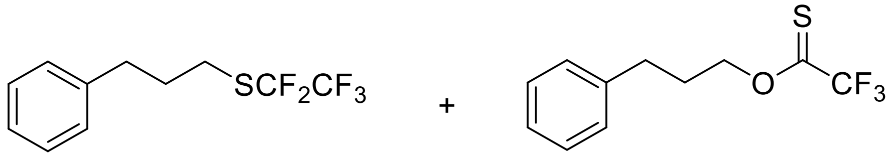

3k **4k**

Prepared from 3-phenylprop-2-yn-1-ol (**2k**) and BT-SC_2F_5 using General Procedure C. Thioether **3k** and thionoester **4k** were isolated as a partially separable mixture. Yellow liquid (76 mg, ratio **3k**:**4k** = 64:36, calculated yield: **3k** = 37%, **4m** = 21%).

3k: ^{1}H NMR (600 MHz, Chloroform-*d*) δ = 7.28 – 7.34 (m, 2H), 7.18 – 7.25 (m, 3H), 2.93 (t, *J* = 7.3 Hz, 2H), 2.76 (t, *J* = 7.6 Hz, 2H), 2.04 (pent, *J* = 7.4 Hz, 2H). **^{19}F NMR (565 MHz, Chloroform-*d*) δ** = -83.4 (t, *J* = 4 Hz, 3F), -91.9 (q, *J* = 4 Hz, 2F).

4k: ^{1}H NMR (600 MHz, Chloroform-*d*) δ = 7.28 – 7.34 (m, 2H), 7.18 – 7.25 (m, 3H), 4.57 (t, *J* = 6.4 Hz, 2H), 2.79 (t, *J* = 7.3 Hz, 2H), 2.21 (m, 2H). **^{19}F NMR (565 MHz, Chloroform-*d*) δ** = -71.0 (3F).

Mixture (definitive assignment of all peaks to 3k or 4k was not possible): ^{13}C NMR (151 MHz, Chloroform-*d*) δ = 196.4 (q, *J* = 39 Hz, C_q), 140.5 (C_q), 140.4 (C_q) 128.8 (CH), 128.7 (CH), 128.57 (CH), 128.56 (CH), 126.5 (CH, 126.4 (CH), 122.0 (tq, *J* = 288, 39 Hz, CF_2), 118.7 (qt, *J* = 286, 37 Hz, CF_3), 115.9 (q, *J* = 280 Hz, CF_3), 73.3 (CH_2), 34.5 (CH_2), 31.9 (CH_2), 31.3 (CH_2), 29.3 (CH_2), 28.0 (t, *J* = 4 Hz, CH_2). **HRMS (EI)**: m/z calculated for $[C_{11}H_{11}F_5S]^+$ ($[M]^+$ for **3k**): 270.0496, measured: 270.0514. *Note: A molecular ion peak for **4k** could not be found, however, a possible fragmentation product was identified: m/z calculated for $[C_{11}H_{10}F_3O]^+$ ($[M-SH]^+$ for **4k**: 215.0678, measured: 215.0676.* **IR (ATR, mixture):** ν (cm^{-1}): 3089, 3068, 3029, 2928, 2859, 1604, 1497, 1455, 1391, 1321, 1289, 1209, 1152, 1097, 1069, 1030, 911, 749, 698, 669, 625, 639, 590, 567, 550.

3.3 Deoxyheptafluoropropylation of Alcohols 2 with BT-SC_3F_7

(Perfluoropropyl)(2,4,6-trichlorobenzyl)sulfane (5d)

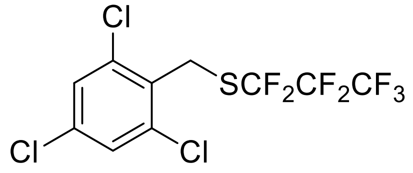

5d

Prepared from 2,4,6-trichlorobenzyl alcohol (**2d**) and BT-SC_3F_7 using General Procedure C. Colourless liquid (196 mg, 0.49 mmol, 98%).

^{1}H NMR (600 MHz, Chloroform-*d*) δ = 7.38 (s, 2H), 4.44 (s, 2H). **^{19}F NMR (565 MHz, Chloroform-*d*) δ** = -80.0 (t, *J* = 9 Hz, 3F), -88.3 (m, 2F), -123.9 (m, 2F). **^{13}C NMR (151 MHz, Chloroform-*d*) δ** = 136.6 (C_q), 135.2 (C_q), 130.1 (C_q), 128.8 (CH), 123.8 (tt, *J* = 290, 34 Hz, SCF_2), 117.8 (qt, *J* = 287, 35 Hz, CF_3), 108.7 (tm, *J* = 265 Hz, CF_2), 28.2 (t, *J* = 5 Hz, CH_2). **HRMS (EI)**: m/z calculated for $[C_{10}H_4Cl_3F_7S]^+$ ($[M]^+$): 393.8982, measured: 393.8983. **IR (ATR):** ν (cm^{-1}): 3083, 2928, 2851, 1726, 1581, 1550, 1528, 1441, 1420, 1376, 1336, 1207, 1184, 1110, 1088, 1072, 1037, 925, 852, 802, 786, 751, 742, 679, 654, 608, 563.

([1,1'-Biphenyl]-4-ylmethyl)(perfluoropropyl)sulfane (5e)

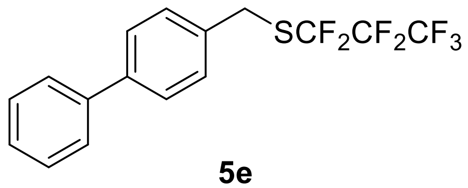

5e

Prepared from biphenyl-4-methanol (**2e**) and BT-SC_3F_7 using General Procedure C. Pale yellow solid (145 mg, 0.394 mmol, 79%).

^{1}H NMR (400 MHz, Chloroform-*d*) δ = 7.59 – 7.65 (m, 4H), 7.44 – 7.52 (m, 4H), 7.41 (m, 1H), 4.25 (s, 2H). **^{19}F NMR (376 MHz, Chloroform-*d*) δ** = -80.0 (t, *J* = 9 Hz, 3F), -88.2 (m, 2F), -124.0 (t, *J* = 4 Hz, 2F). **^{13}C NMR (101 MHz, Chloroform-*d*) δ** = 141.3 (C_q), 140.6 (C_q), 133.6 (C_q), 129.7 (CH), 129.0 (CH), 127.8 (CH), 127.7 (CH), 127.3 (CH), 123.9 (tt, *J* = 289, 34 Hz, SCF_2), 117.9 (qt, *J* = 288, 34 Hz, CF_3), 108.9 (tm, *J* = 265 Hz, CF_2), 32.8 (t, *J* = 4 Hz, CH_2). **HRMS (EI)**: m/z calculated for $[C_{16}H_{11}F_7S]^+$ ($[M]^+$): 368.0464, measured: 368.0476. **IR (ATR):** ν (cm^{-1}): 3074, 3032, 1520, 1488, 1445, 1407, 1335, 1207, 1183, 1112, 1089, 1037, 1007, 924, 857, 769, 740, 717, 695, 673, 610.

4 NMR Spectra of Novel Compounds

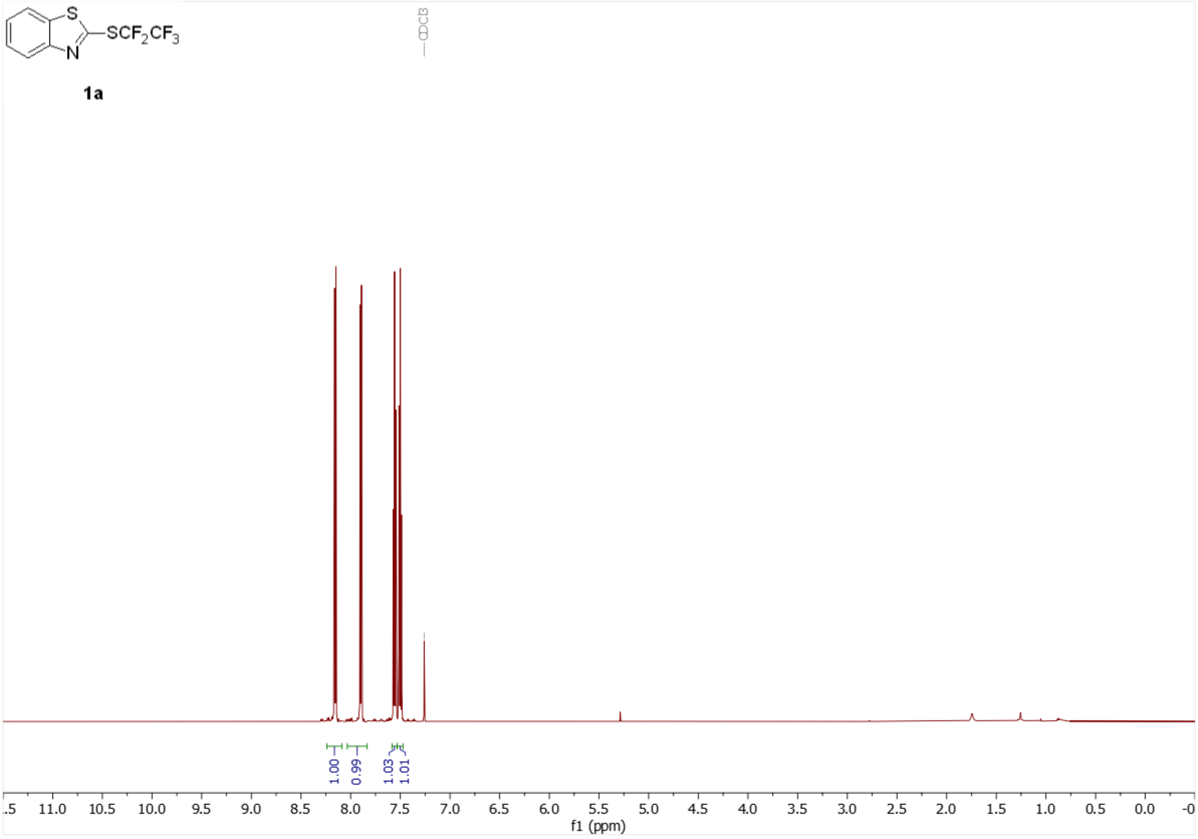

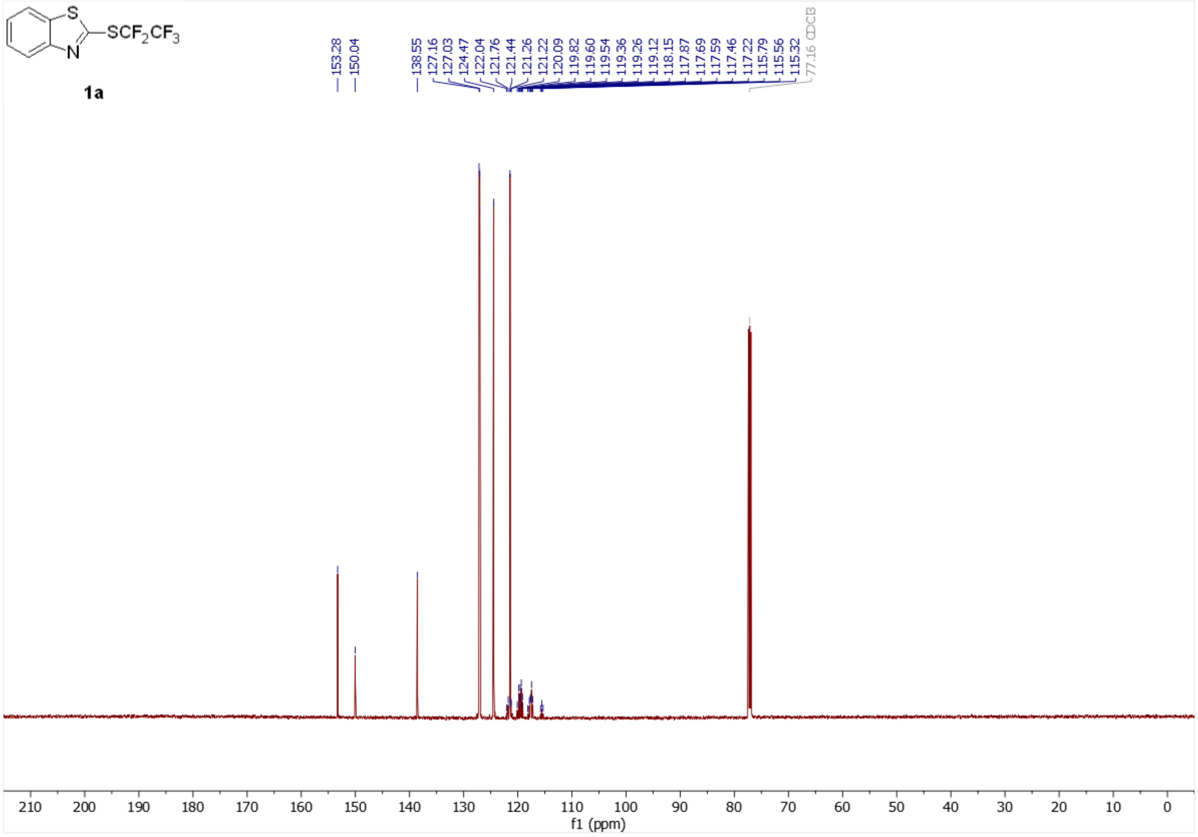

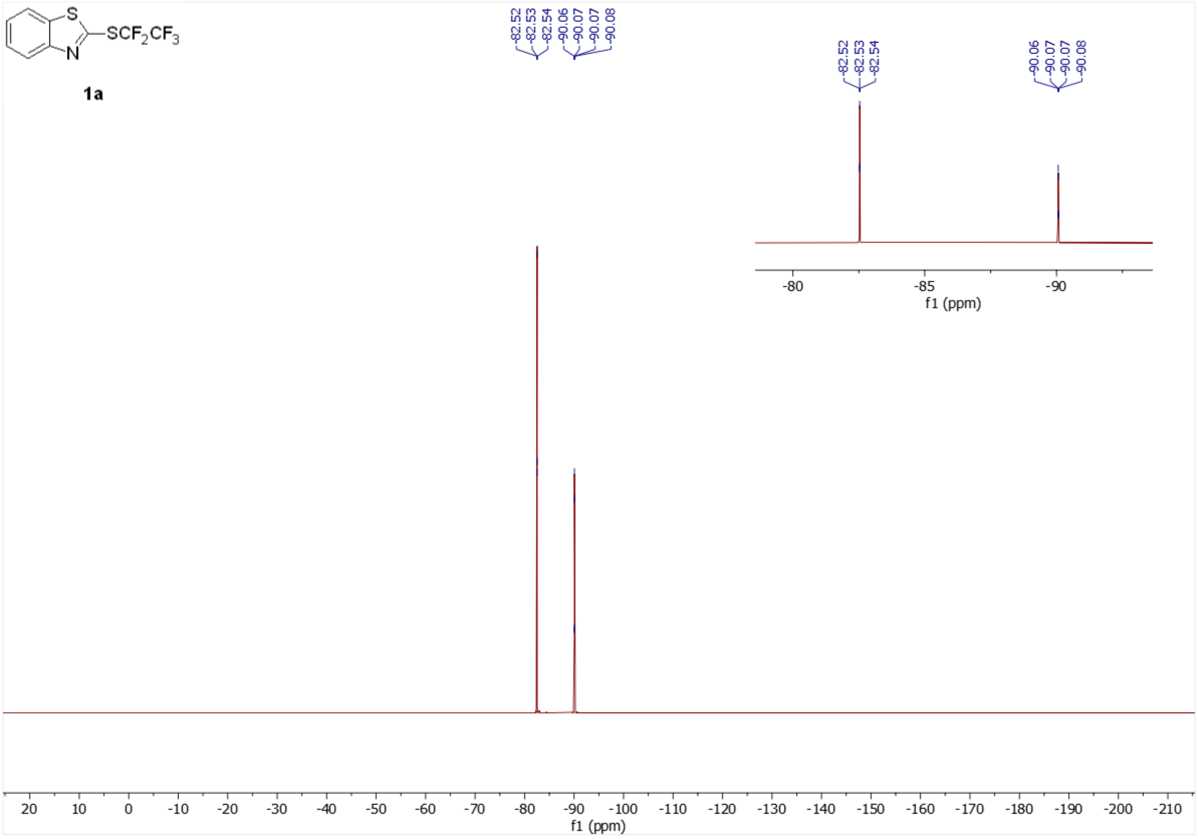
SCF2CF3
1a
-82.52
-82.53
-82.54
-90.06
-90.07
-90.07
-90.08
-80
-85
-90
f1 (ppm)
20
10
0
-10
-20
-30
-40
-50
-60
-70
-80
-90
-100
-110
-120
-130
-140
-150
-160
-170
-180
-190
-200
-210
f1 (ppm)

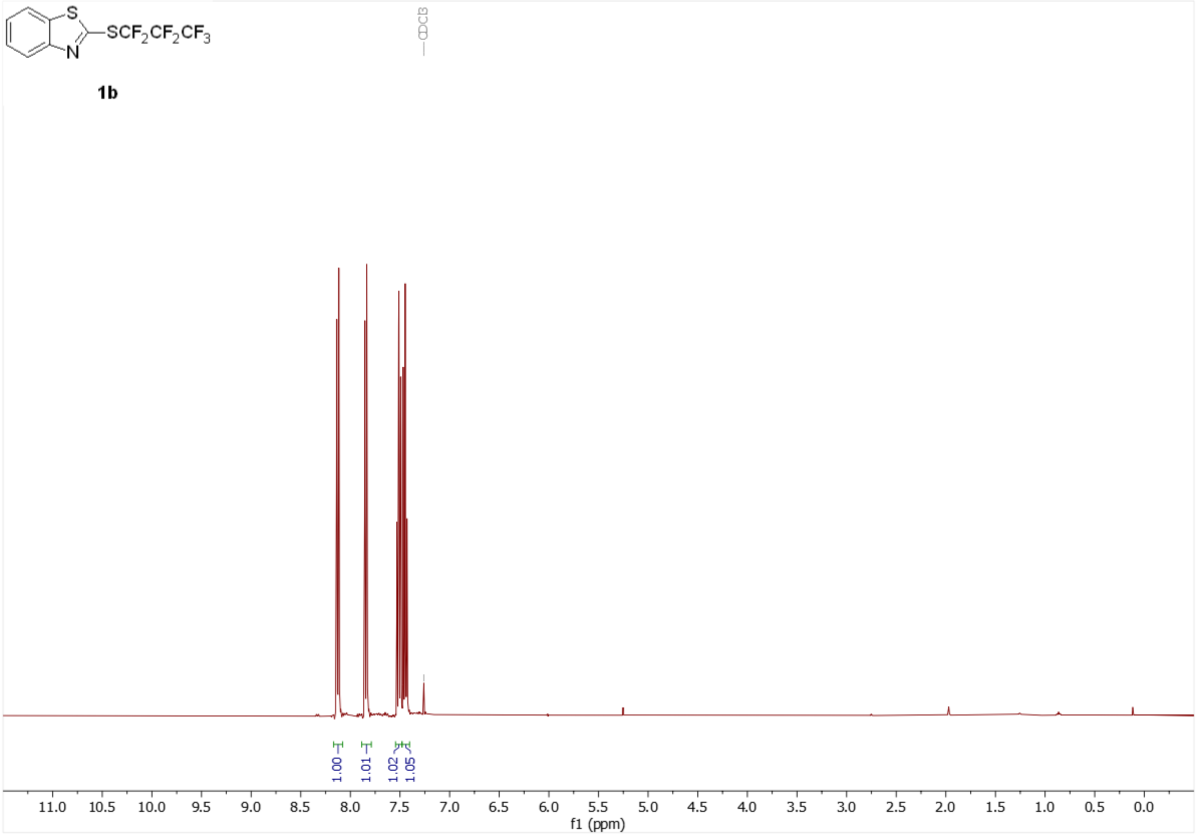
SCF2CF2CF3
1b
CDCl3
1.00
1.01
1.02
1.05
11.0
10.5
10.0
9.5
9.0
8.5
8.0
7.5
7.0
6.5
6.0
5.5
5.0
4.5
4.0
3.5
3.0
2.5
2.0
1.5
1.0
0.5
0.0
f1 (ppm)

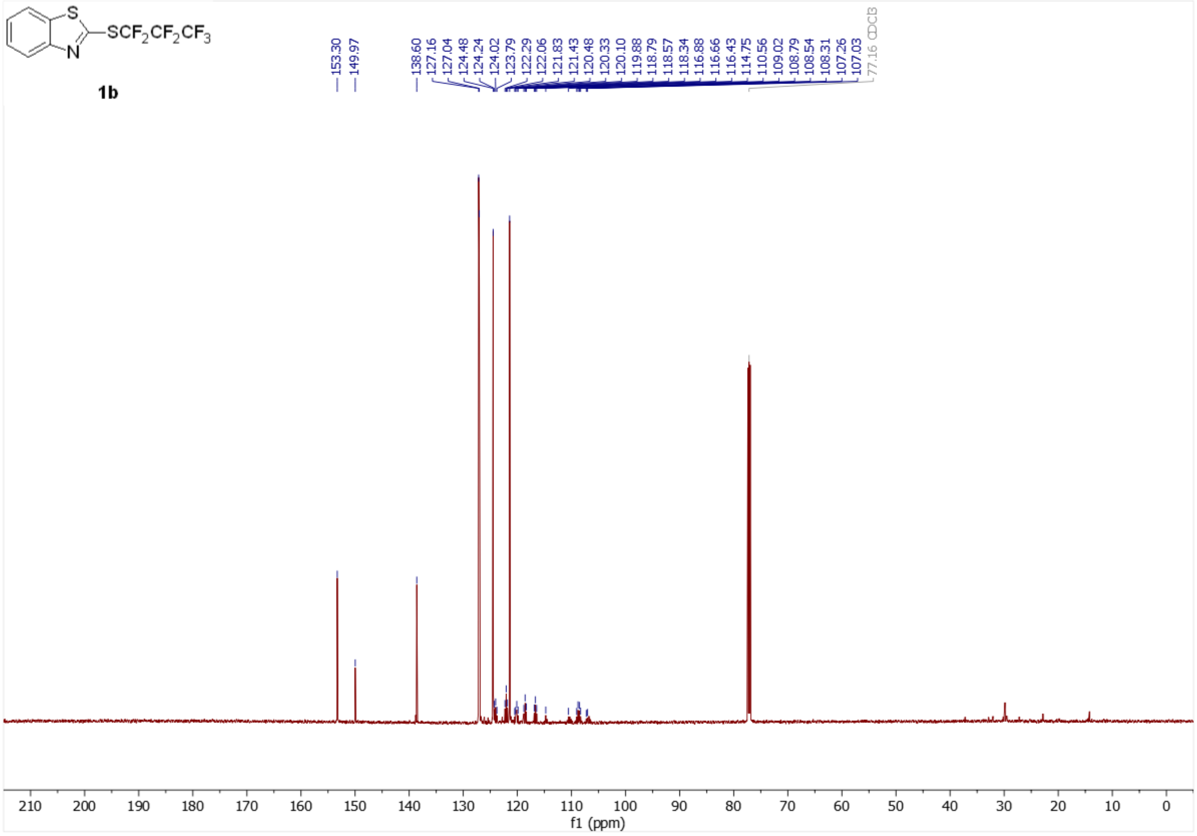
SCF2CF2CF3
1b
153.30
149.97
138.60
127.16
127.04
124.48
124.24
124.02
123.79
122.29
122.06
121.83
121.43
120.48
120.33
120.10
119.88
118.79
118.57
118.34
116.88
116.66
116.43
114.75
110.56
109.02
108.79
108.54
108.31
107.26
107.03
77.16 CDCl3
f1 (ppm)

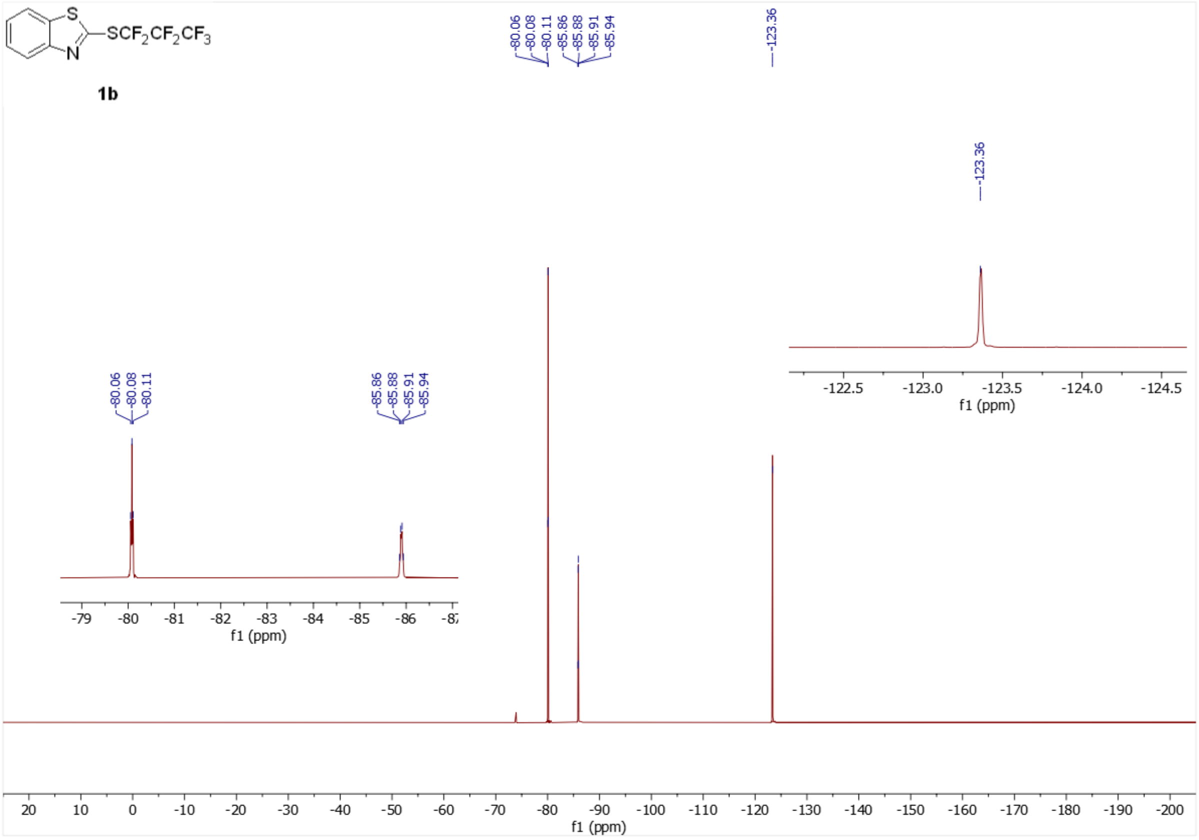
SCF2CF2CF3
1b
-80.06
-80.08
-80.11
-85.86
-85.88
-85.91
-85.94
-123.36
f1 (ppm)

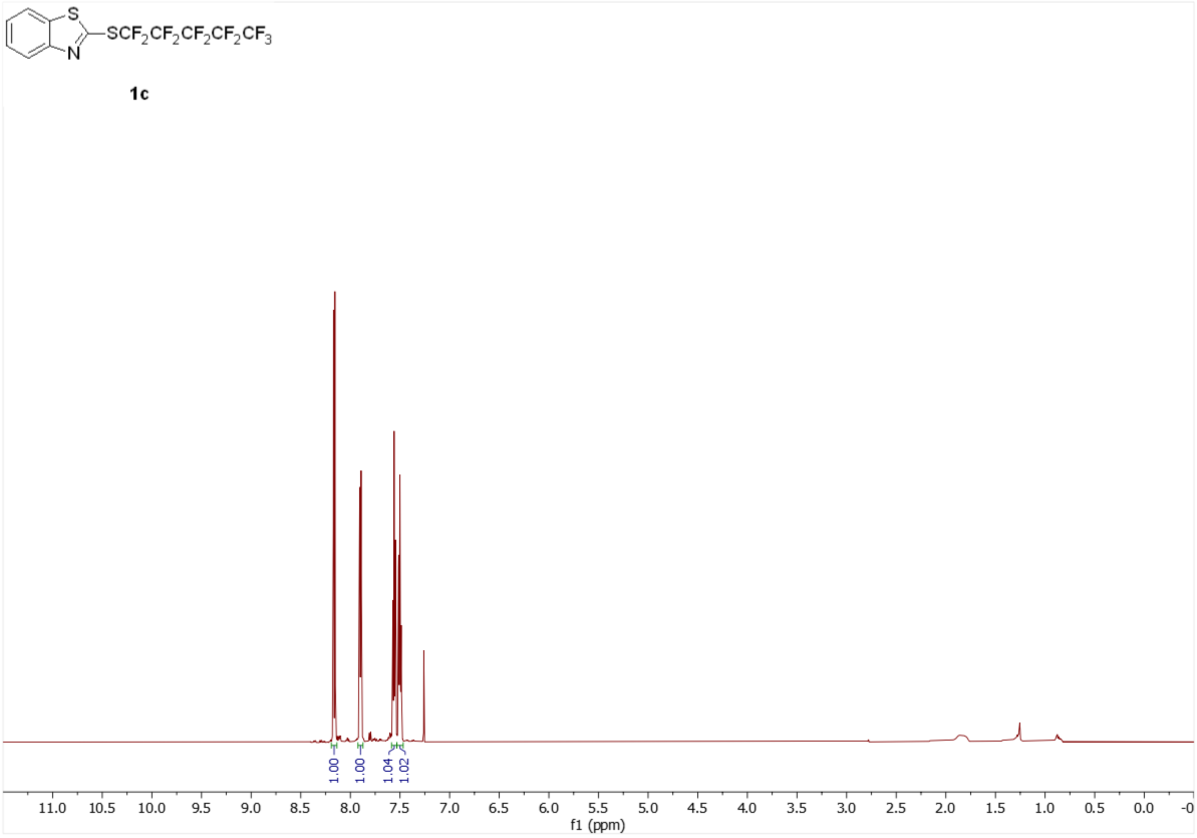
SCF2CF2CF2CF2CF3
1c
1.00
1.00
1.04
1.02
11.0 10.5 10.0 9.5 9.0 8.5 8.0 7.5 7.0 6.5 6.0 5.5 5.0 4.5 4.0 3.5 3.0 2.5 2.0 1.5 1.0 0.5 0.0
f1 (ppm)

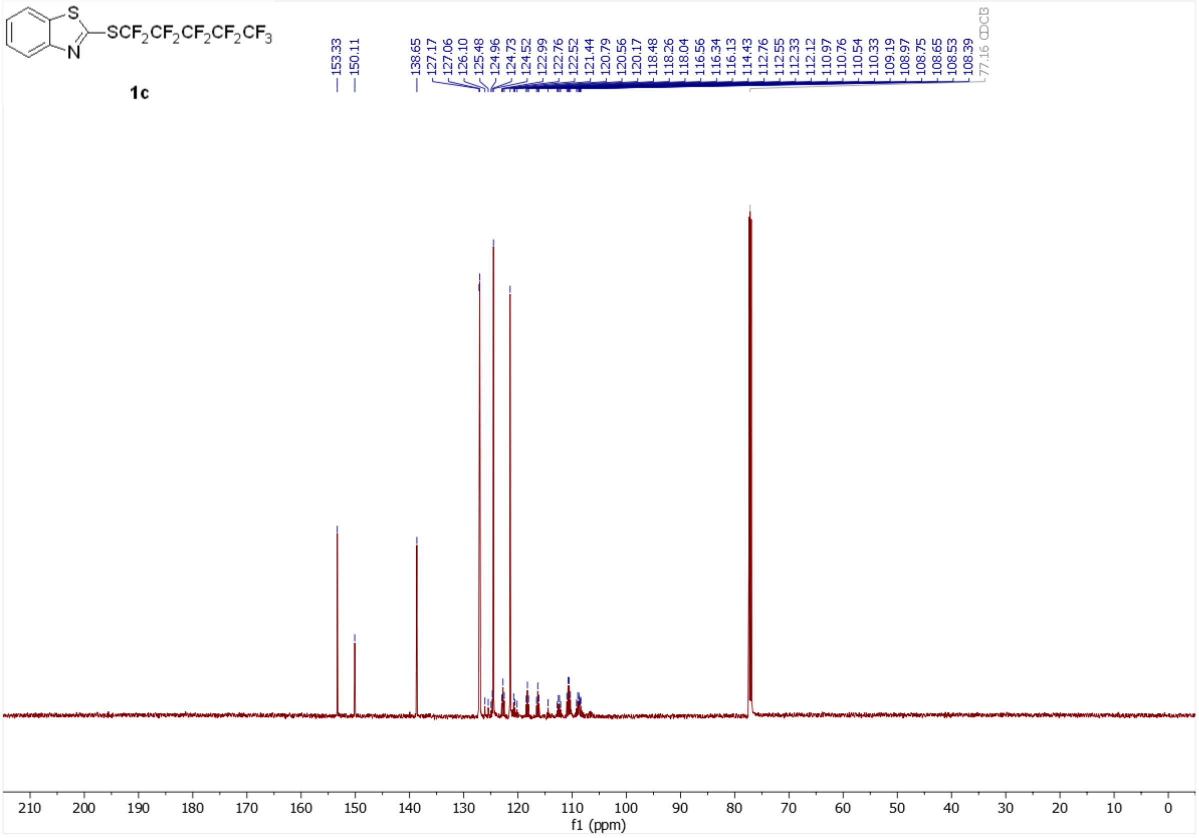
SCF2CF2CF2CF2CF3
1c
153.33
150.11
138.65
127.17
127.06
126.10
125.48
124.96
124.73
124.52
122.99
122.76
122.52
121.44
120.79
120.56
120.17
118.48
118.26
118.04
116.56
116.34
116.13
114.43
112.76
112.55
112.33
112.12
110.97
110.76
110.54
110.33
109.19
108.97
108.75
108.65
108.53
108.39
77.16 CDCl3
210 200 190 180 170 160 150 140 130 120 110 100 90 80 70 60 50 40 30 20 10 0
f1 (ppm)

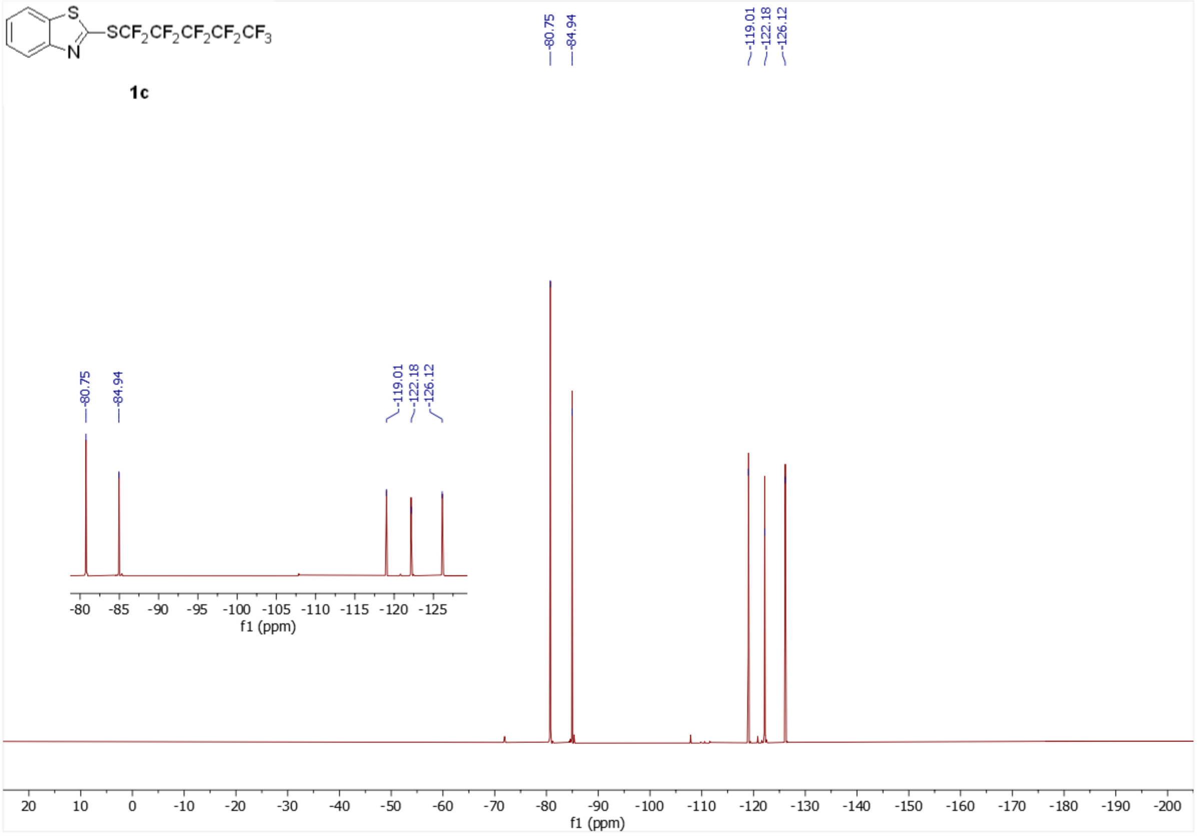
SCF2CF2CF2CF2CF3
1c
-80.75
-84.94
-119.01
-122.18
-126.12
f1 (ppm)

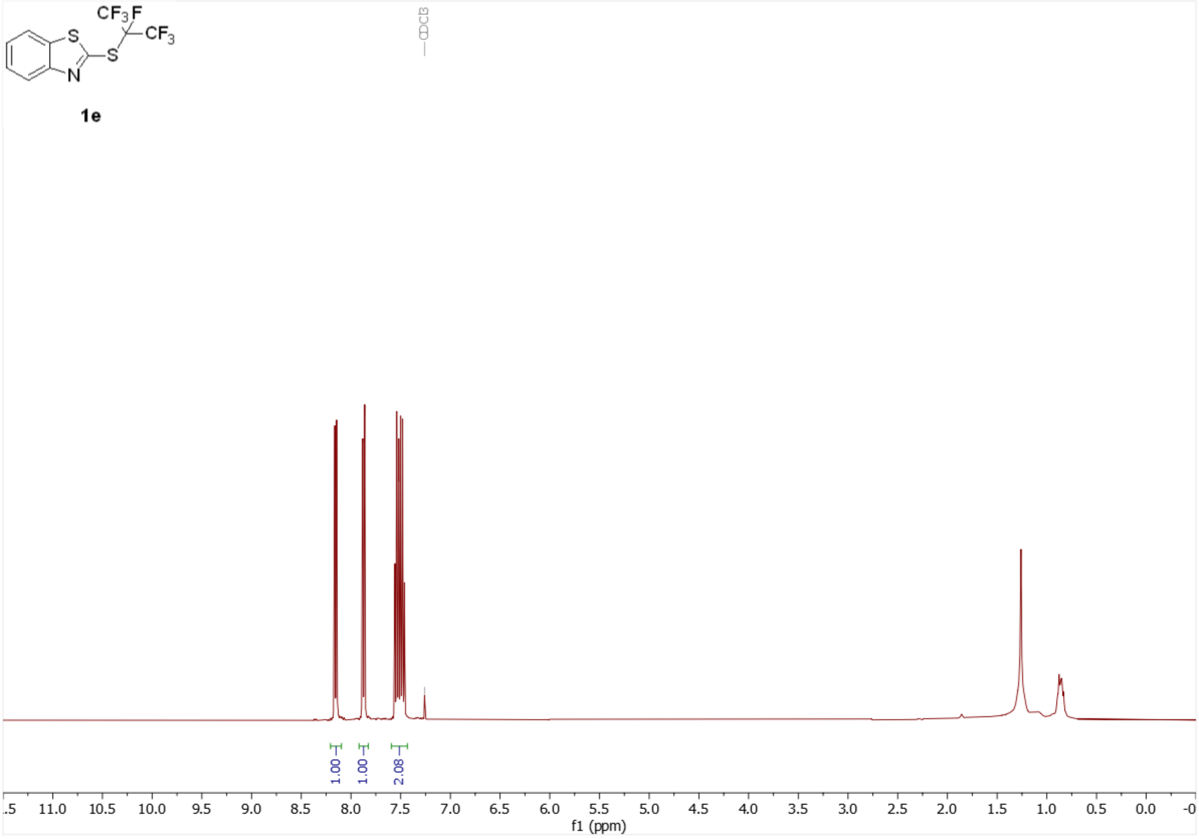
CF3
F
CF3
S
N
1e
CDCl3
1.00
1.00
2.08
f1 (ppm)

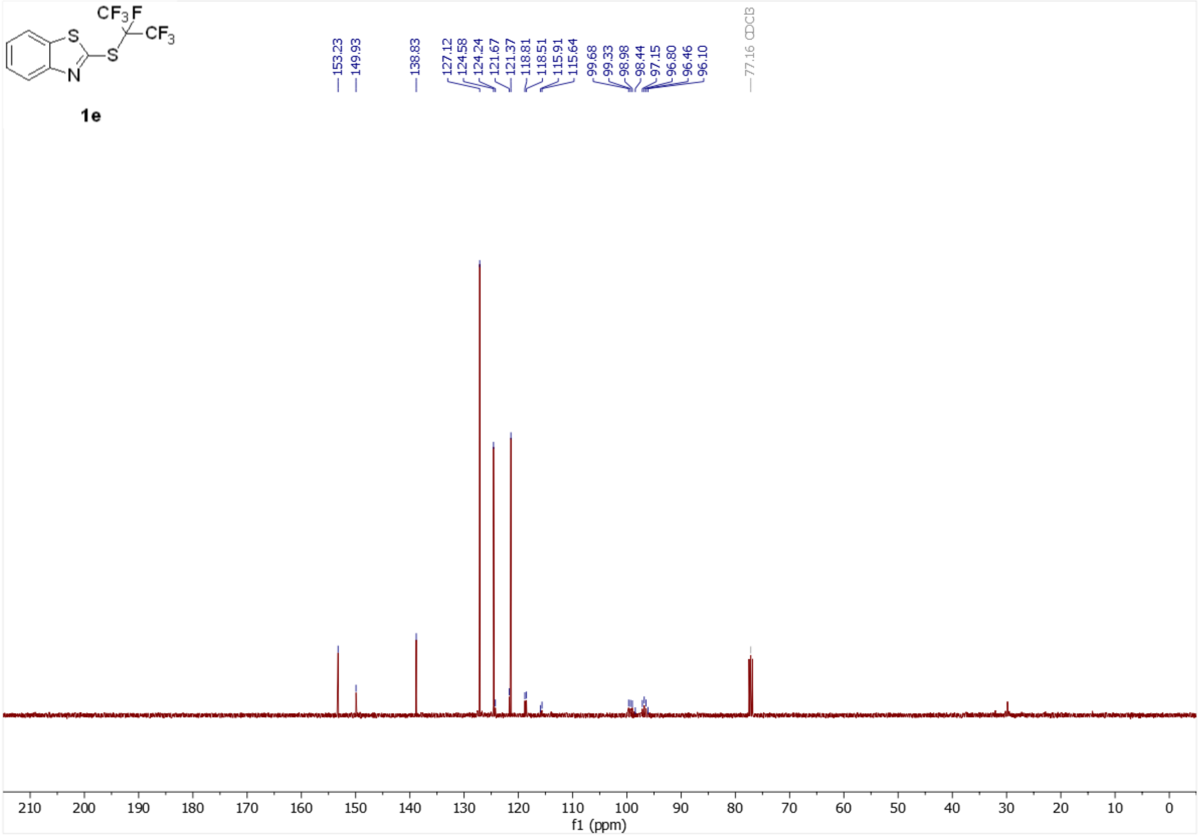
CF3 F
CF3
S
S
N
1e
153.23
149.93
138.83
127.12
124.58
124.24
121.67
121.37
118.81
118.51
115.91
115.64
99.68
99.33
98.98
98.44
97.15
96.80
96.46
96.10
77.16 CDCl3
210 200 190 180 170 160 150 140 130 120 110 100 90 80 70 60 50 40 30 20 10 0
f1 (ppm)

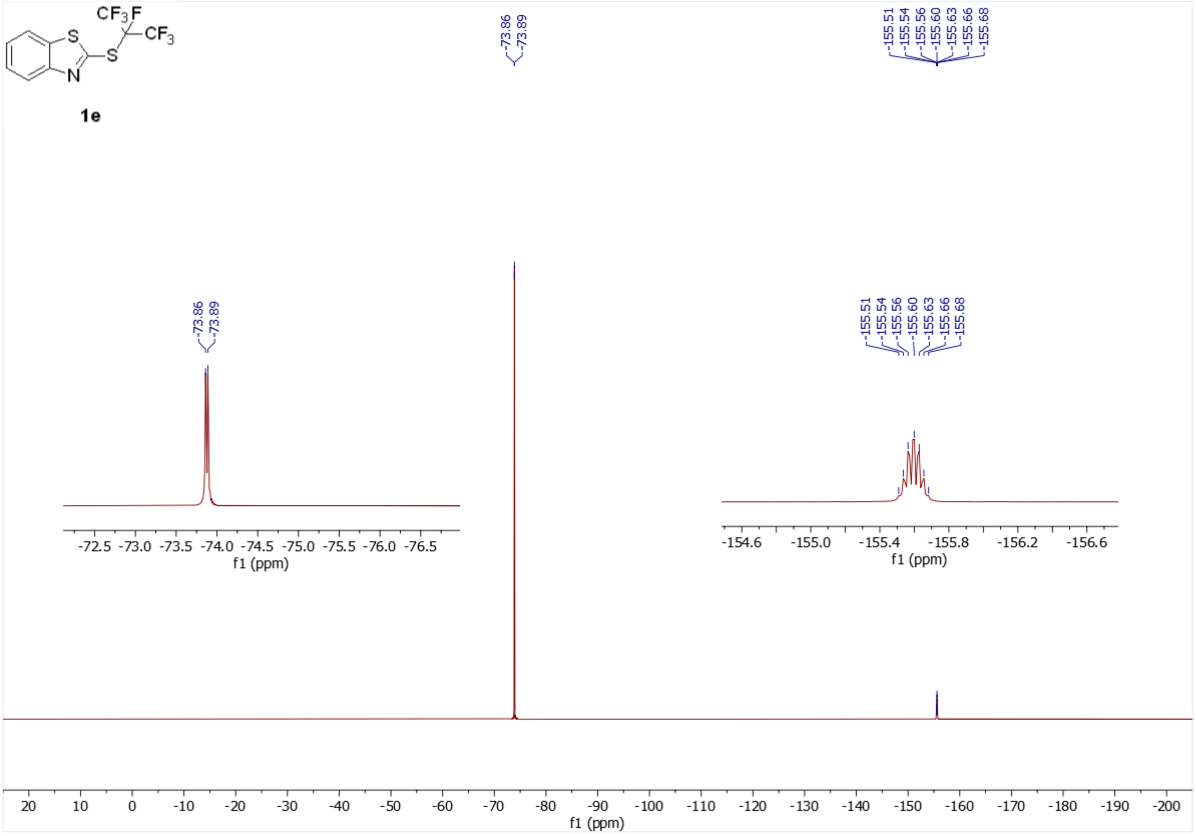
CF3 F
CF3
S
S
N
1e
-73.86
-73.89
-155.51
-155.54
-155.56
-155.60
-155.63
-155.66
-155.68
-72.5 -73.0 -73.5 -74.0 -74.5 -75.0 -75.5 -76.0 -76.5
f1 (ppm)
-154.6 -155.0 -155.4 -155.8 -156.2 -156.6
f1 (ppm)
20 10 0 -10 -20 -30 -40 -50 -60 -70 -80 -90 -100 -110 -120 -130 -140 -150 -160 -170 -180 -190 -200
f1 (ppm)

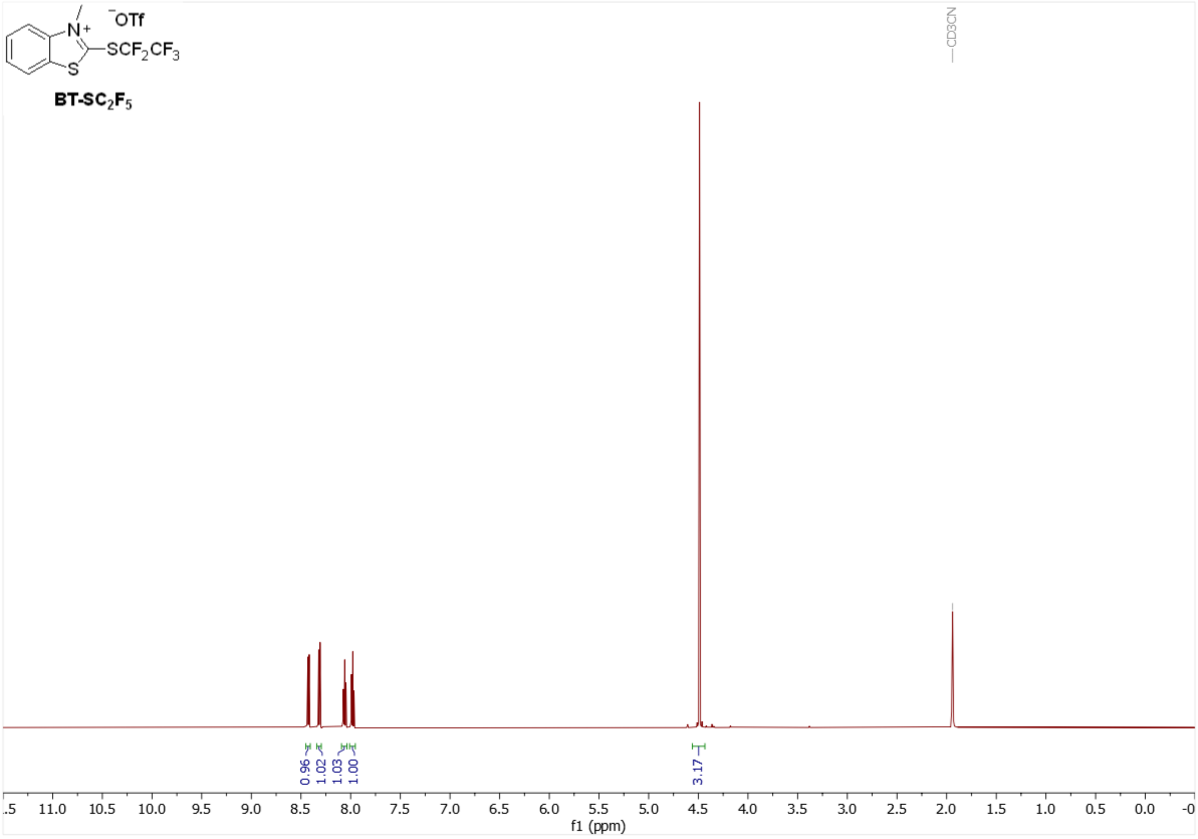
BT-SC2F5
OTf
SCF2CF3
CD3CN
0.96
1.02
1.03
1.00
3.17
f1 (ppm)

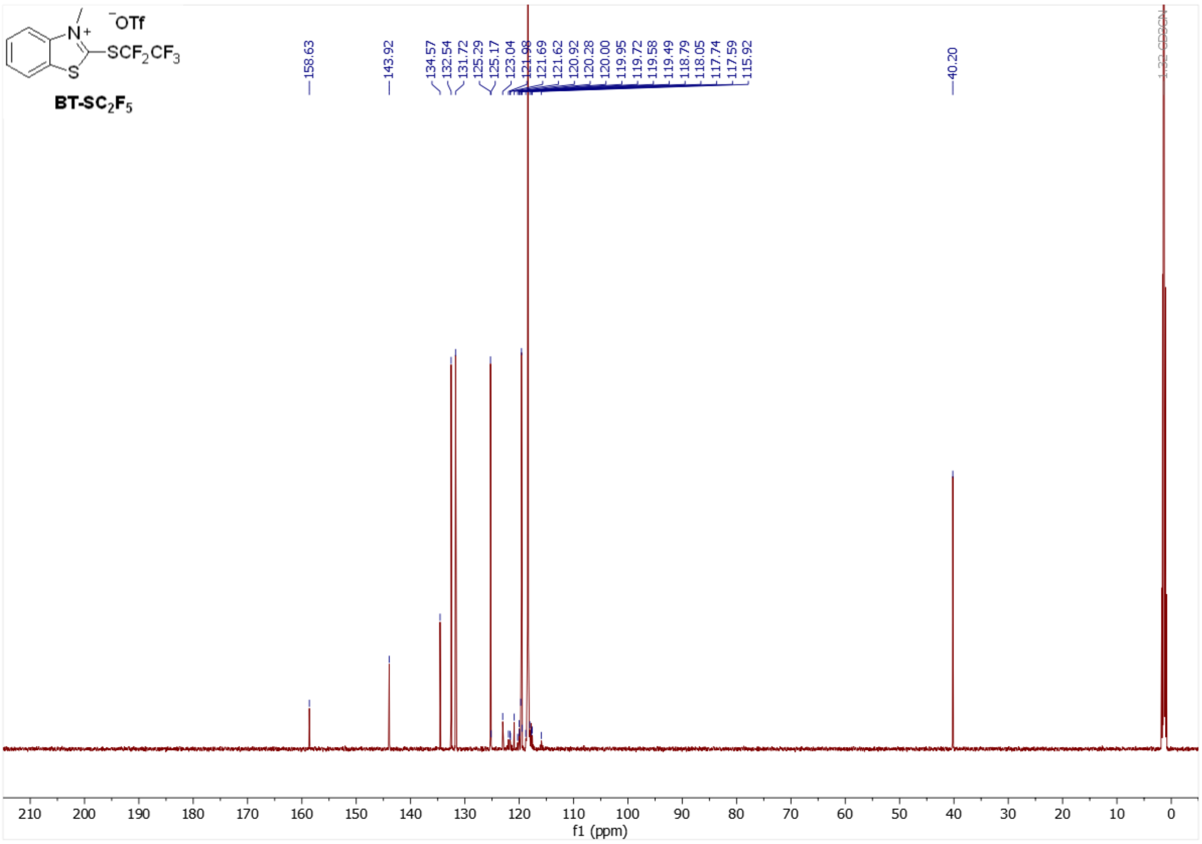
BT-SC2F5
OTf
SCF2CF3
158.63
143.92
134.57
132.54
131.72
125.29
125.17
123.04
121.98
121.69
121.62
120.92
120.28
120.00
119.95
119.72
119.58
119.49
118.79
118.05
117.74
117.59
115.92
40.20
f1 (ppm)

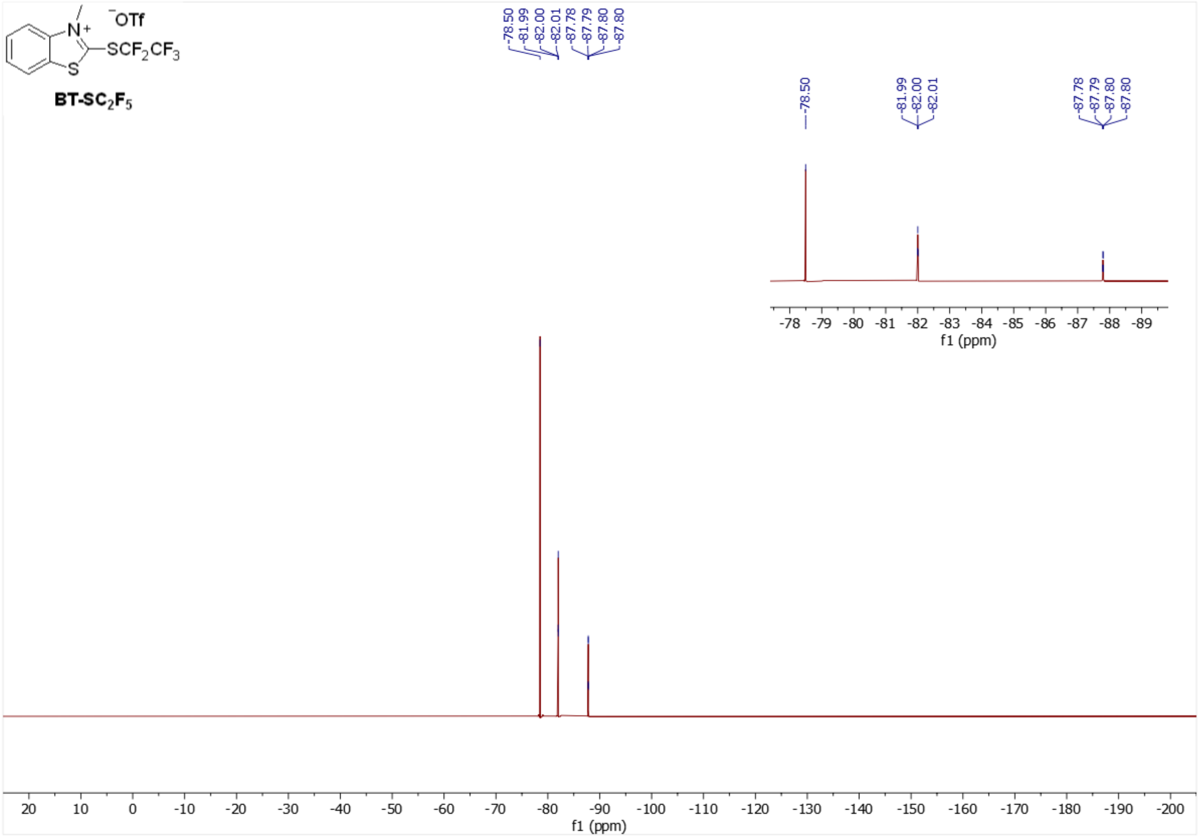

OTf
SCF2CF3
BT-SC2F5
-78.50
-81.99
-82.00
-82.01
-87.78
-87.79
-87.80
-87.80
f1 (ppm)

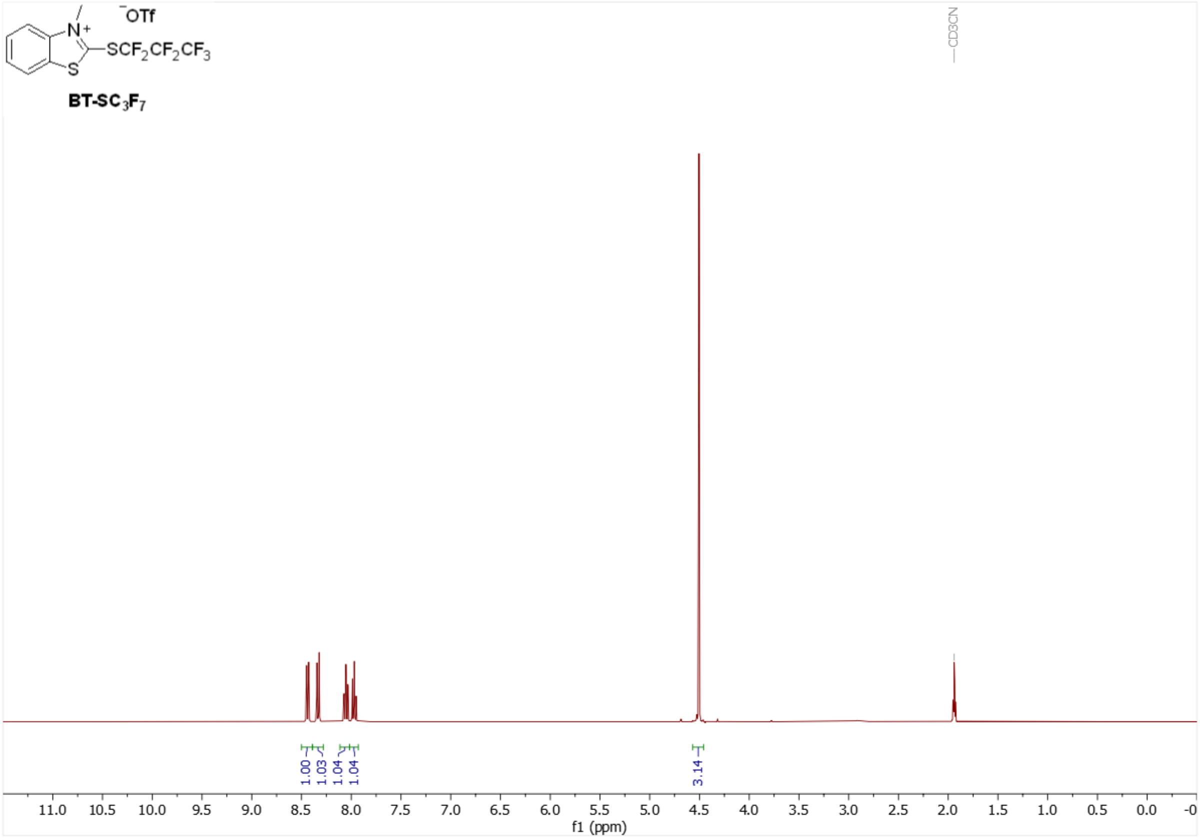

OTf
SCF2CF2CF3
BT-SC3F7
CD3CN
1.00
1.03
1.04
1.04
3.14
f1 (ppm)

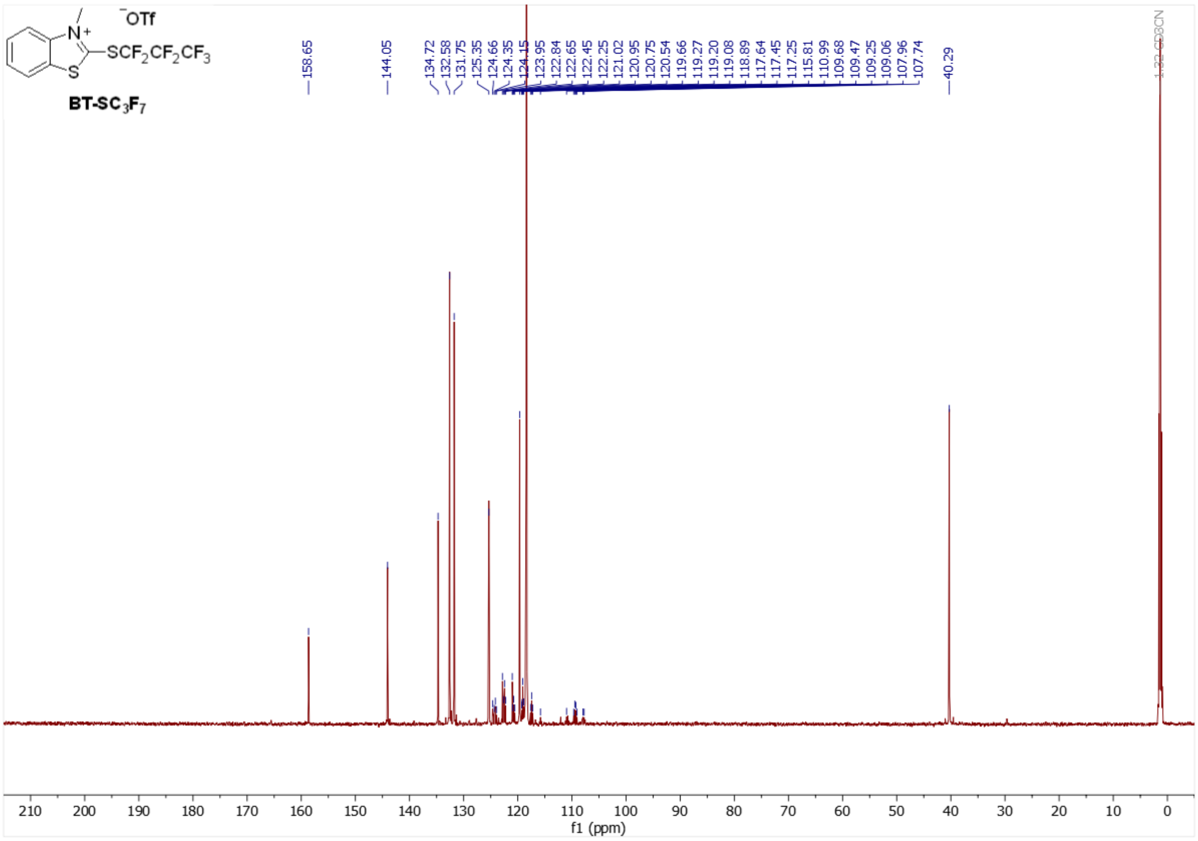

⁻OTf
SCF2CF2CF3
BT-SC3F7
158.65
144.05
134.72
132.58
131.75
125.35
124.66
124.35
124.15
123.95
122.84
122.65
122.45
122.25
121.02
120.95
120.75
120.54
119.66
119.27
119.20
119.08
118.89
117.64
117.45
117.25
115.81
110.99
109.68
109.47
109.25
109.06
107.96
107.74
40.29
f1 (ppm)

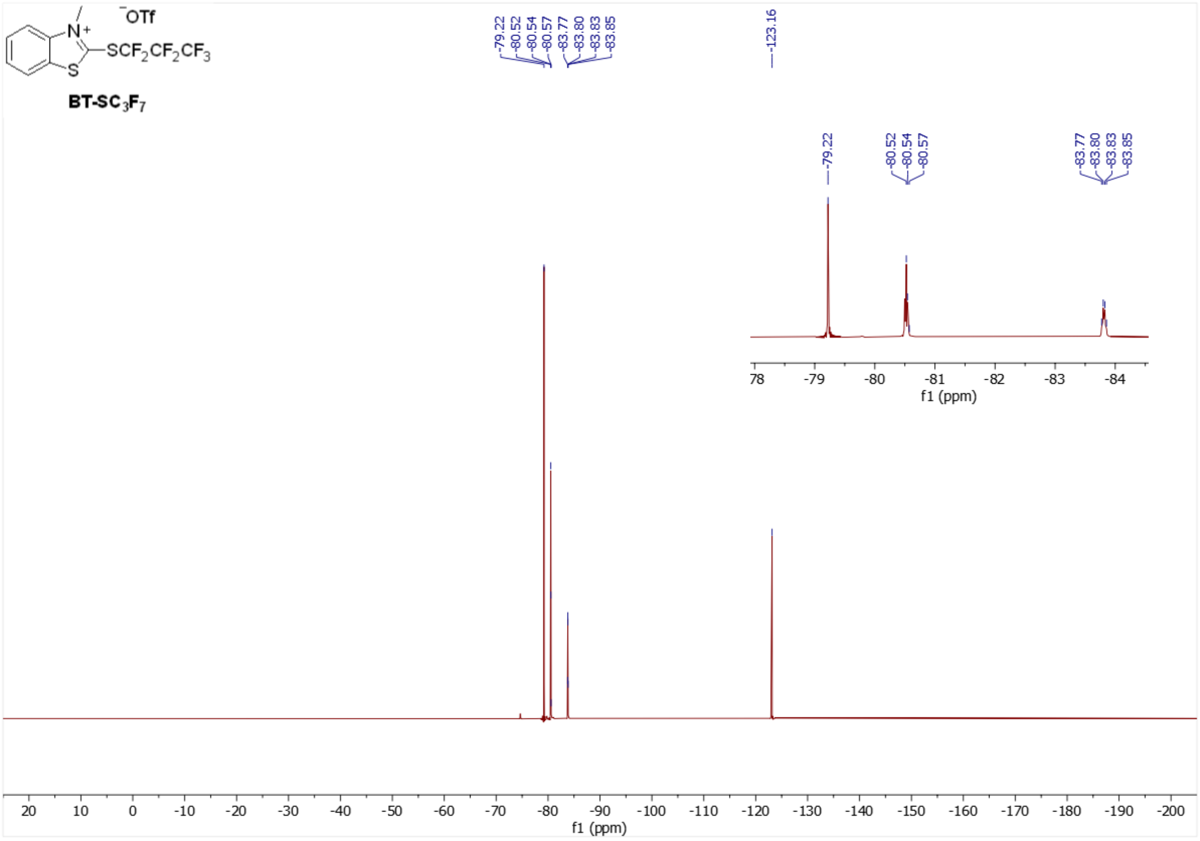

⁻OTf
SCF2CF2CF3
BT-SC3F7
-79.22
-80.52
-80.54
-80.57
-83.77
-83.80
-83.83
-83.85
-123.16
f1 (ppm)

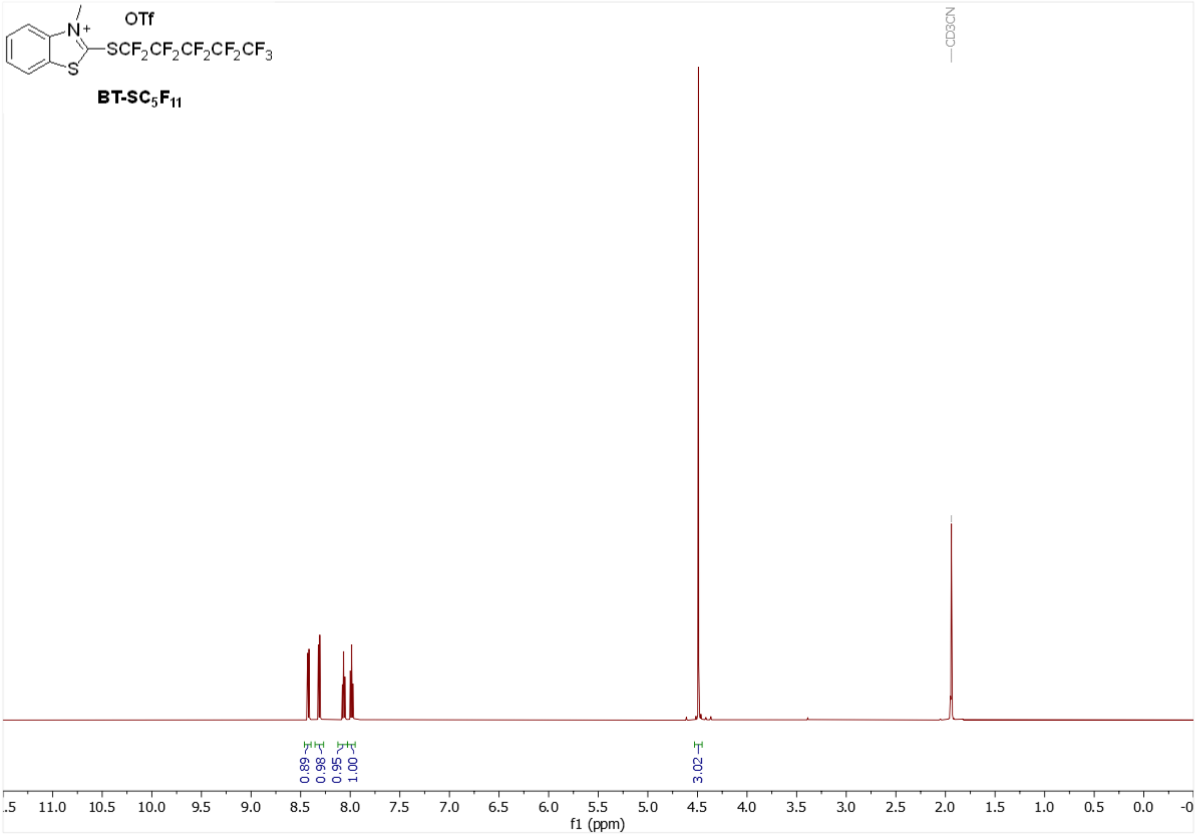
OTf
BT-SC5F11
CD3CN
0.89
0.98
0.95
1.00
3.02
f1 (ppm)

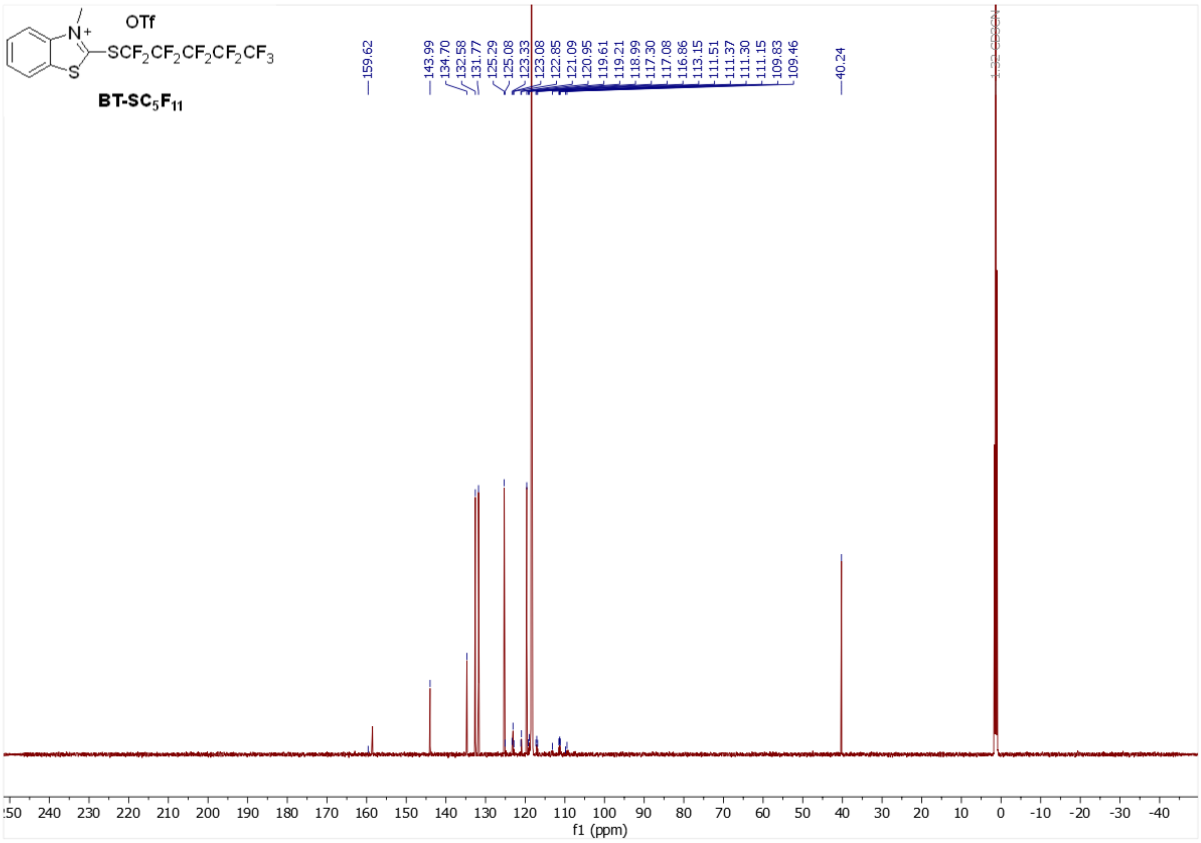
OTf
BT-SC5F11
159.62
143.99
134.70
132.58
131.77
125.29
125.08
123.33
123.08
122.85
121.09
120.95
119.61
119.21
118.99
117.30
117.08
116.86
113.15
111.51
111.37
111.30
111.15
109.83
109.46
40.24
f1 (ppm)

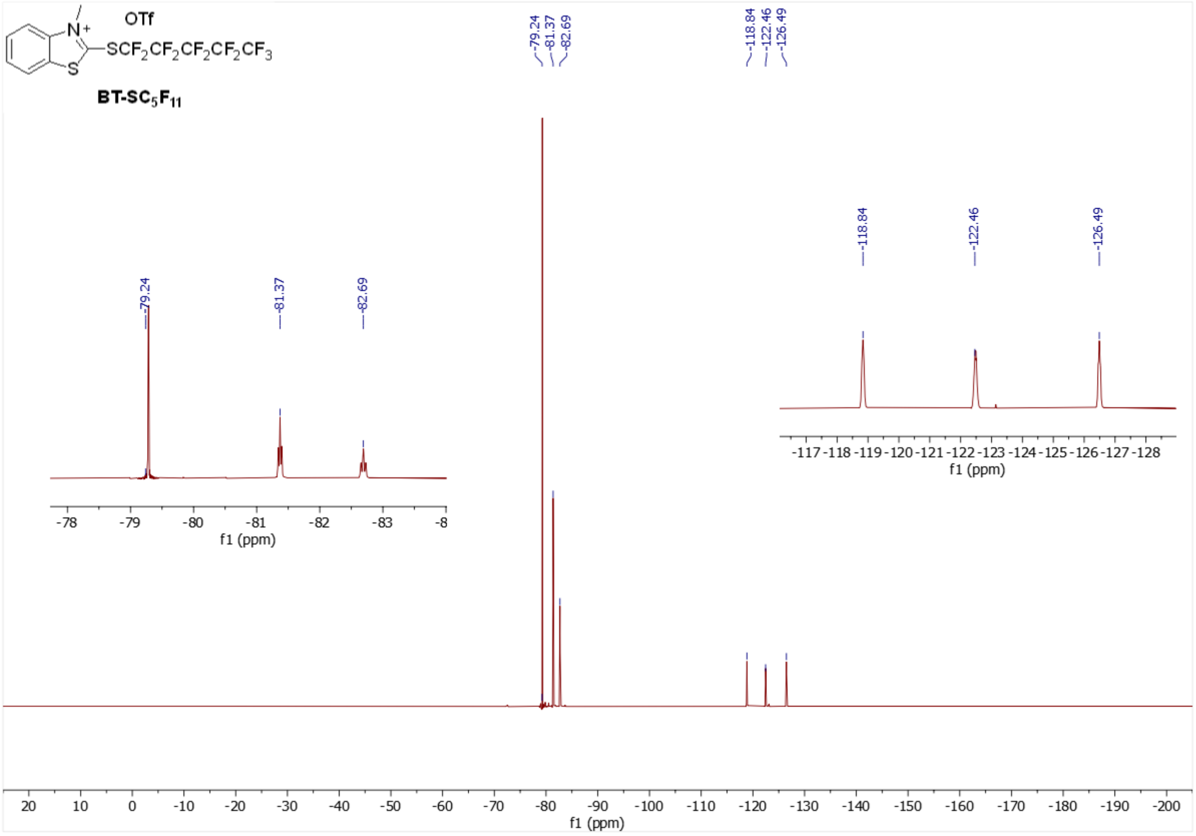
OTf
SCF2CF2CF2CF2CF3
BT-SC5F11
-79.24
-81.37
-82.69
-118.84
-122.46
-126.49
f1 (ppm)

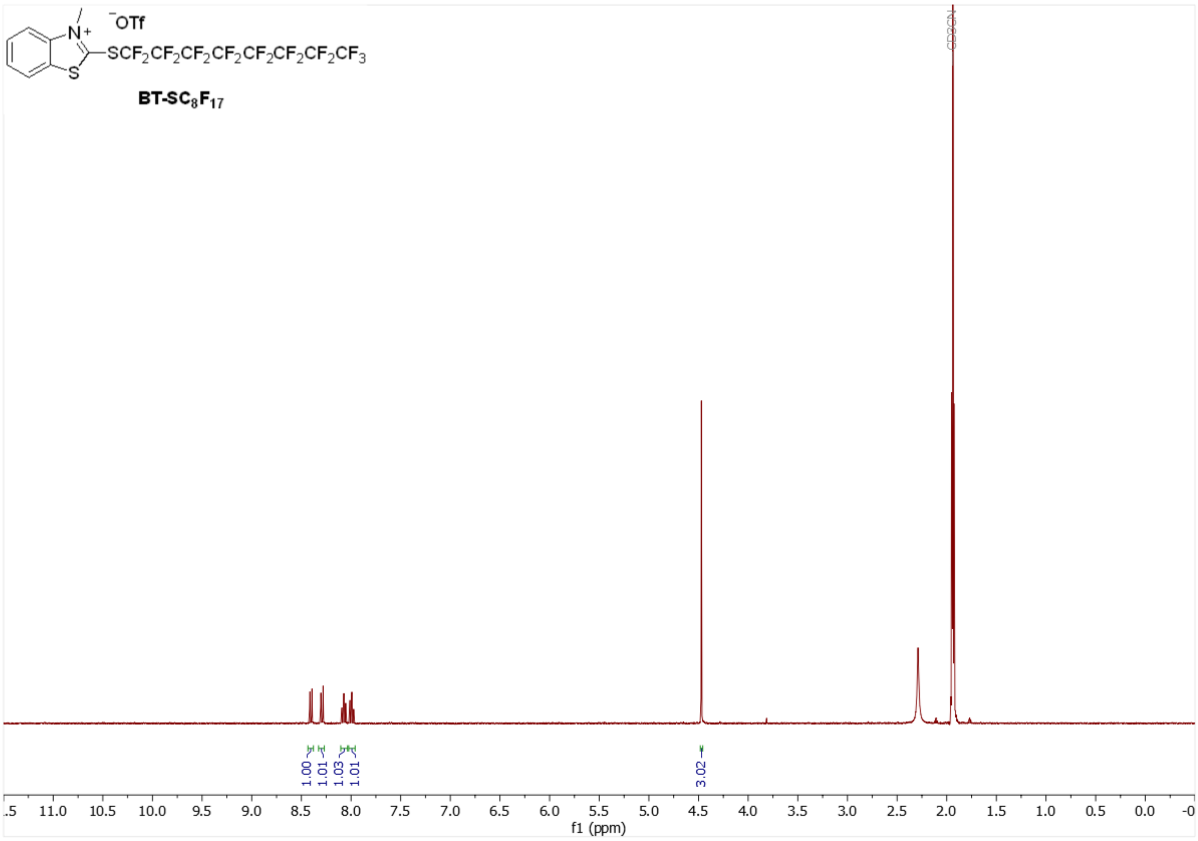
-OTf
SCF2CF2CF2CF2CF2CF2CF2CF3
BT-SC8F17
CD3CN
1.00
1.01
1.03
1.01
3.02
f1 (ppm)

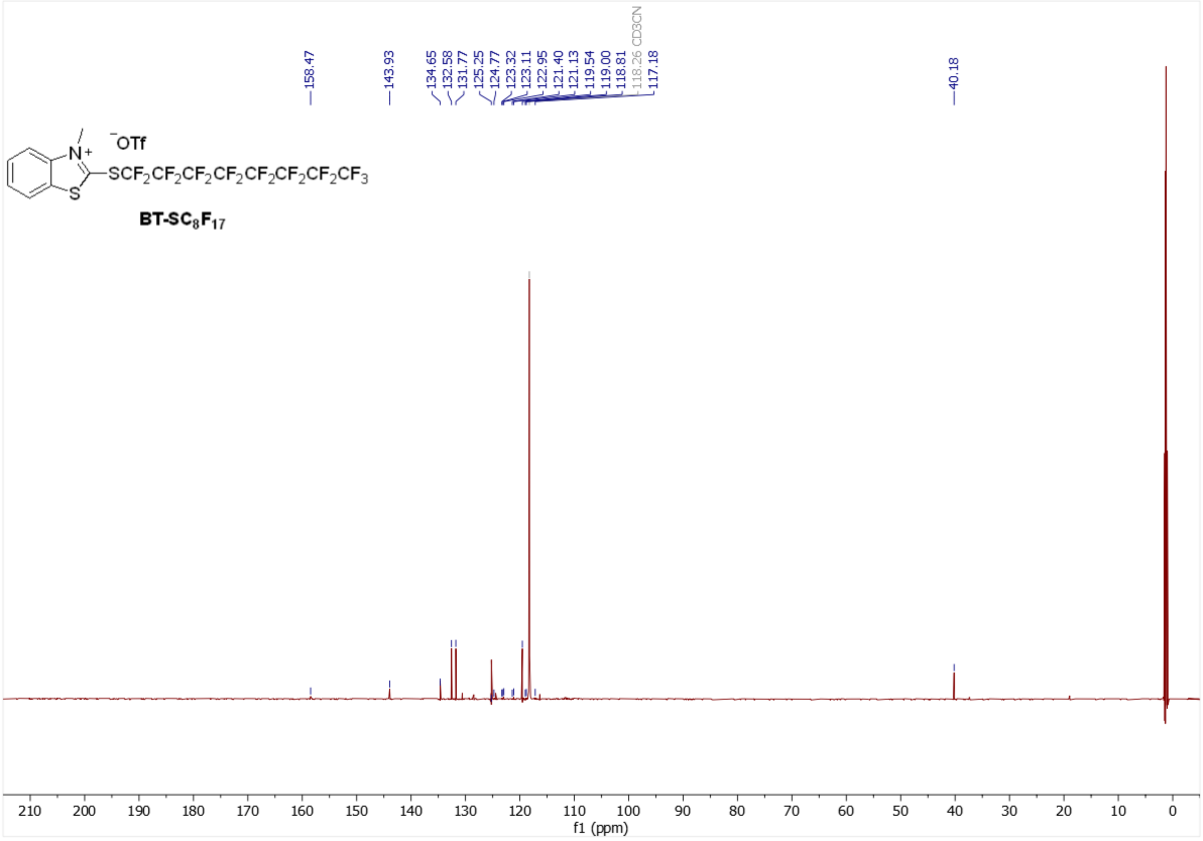
158.47
143.93
134.65
132.58
131.77
125.25
124.77
123.32
123.11
122.95
121.40
121.13
119.54
119.00
118.81
118.26 CD3CN
117.18
40.18
⁻OTf
N+
S
SCF2CF2CF2CF2CF2CF2CF2CF3
BT-SC8F17
f1 (ppm)

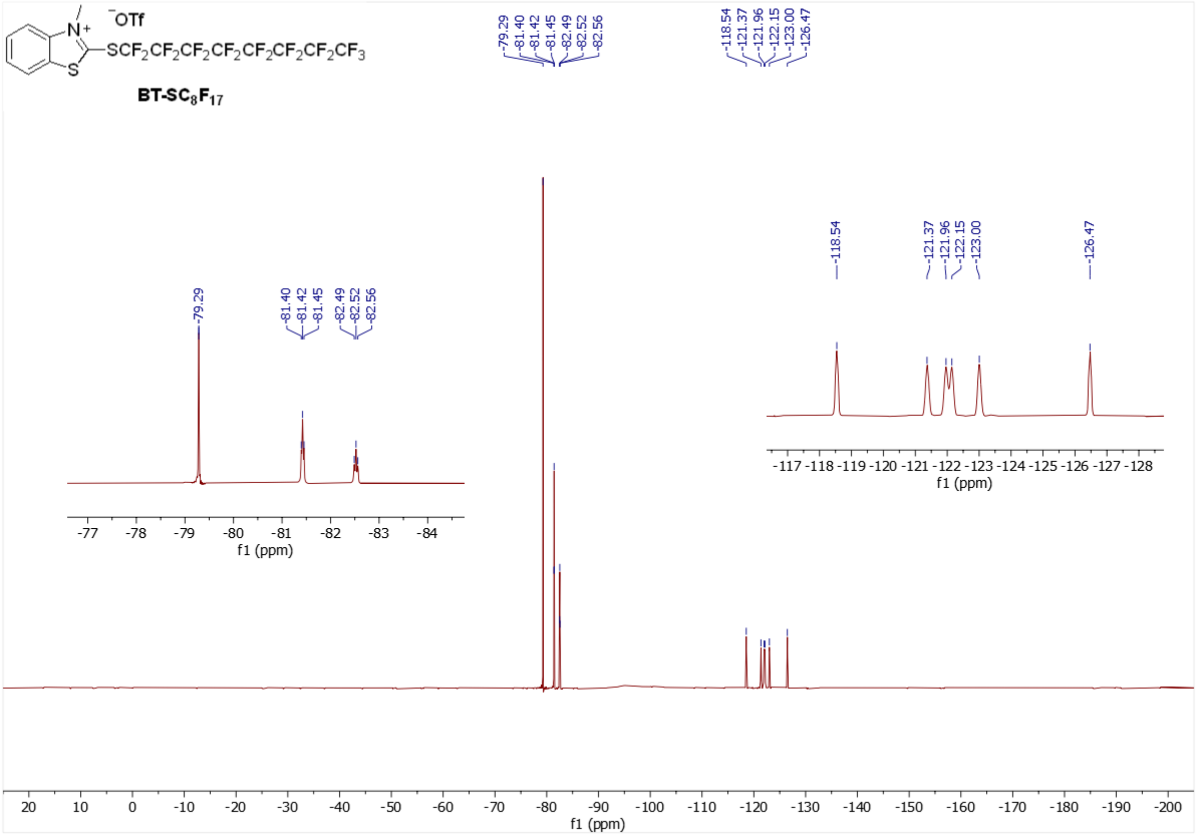
-79.29
-81.40
-81.42
-81.45
-82.49
-82.52
-82.56
-118.54
-121.37
-121.96
-122.15
-123.00
-126.47
⁻OTf
N+
S
SCF2CF2CF2CF2CF2CF2CF2CF3
BT-SC8F17
f1 (ppm)

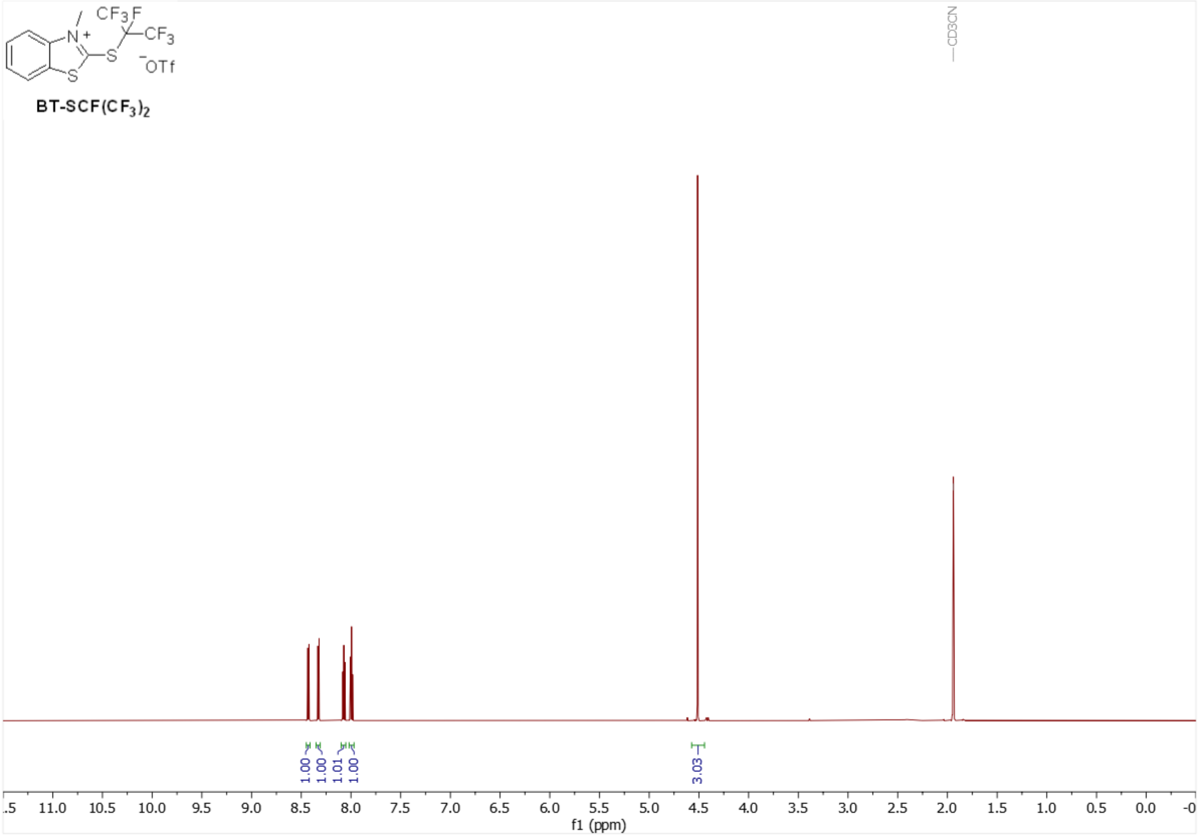
BT-SCF(CF3)2
OTf
CD3CN
1.00
1.00
1.01
1.00
3.03
f1 (ppm)

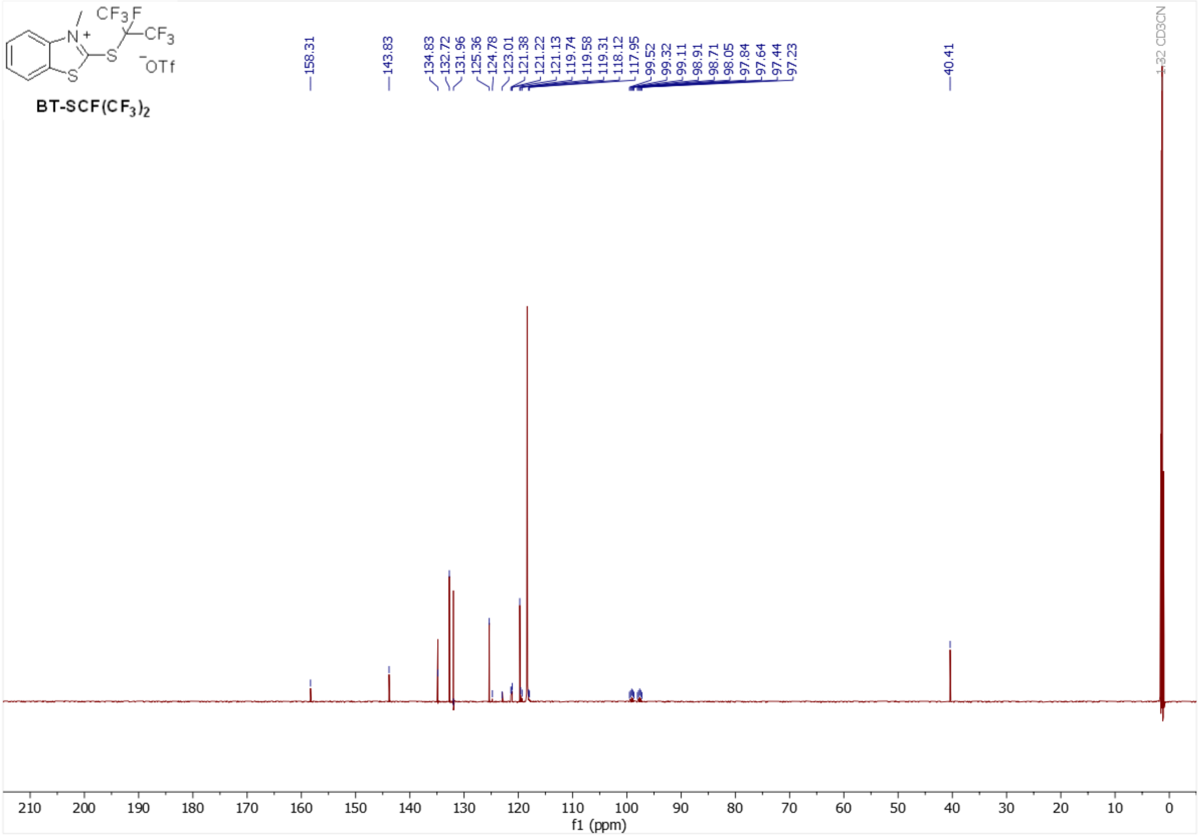
BT-SCF(CF3)2
OTf
158.31
143.83
134.83
132.72
131.96
125.36
124.78
123.01
121.38
121.22
121.13
119.74
119.58
119.31
118.12
117.95
99.52
99.32
99.11
98.91
98.71
98.05
97.84
97.64
97.44
97.23
40.41
1.32 CD3CN
f1 (ppm)

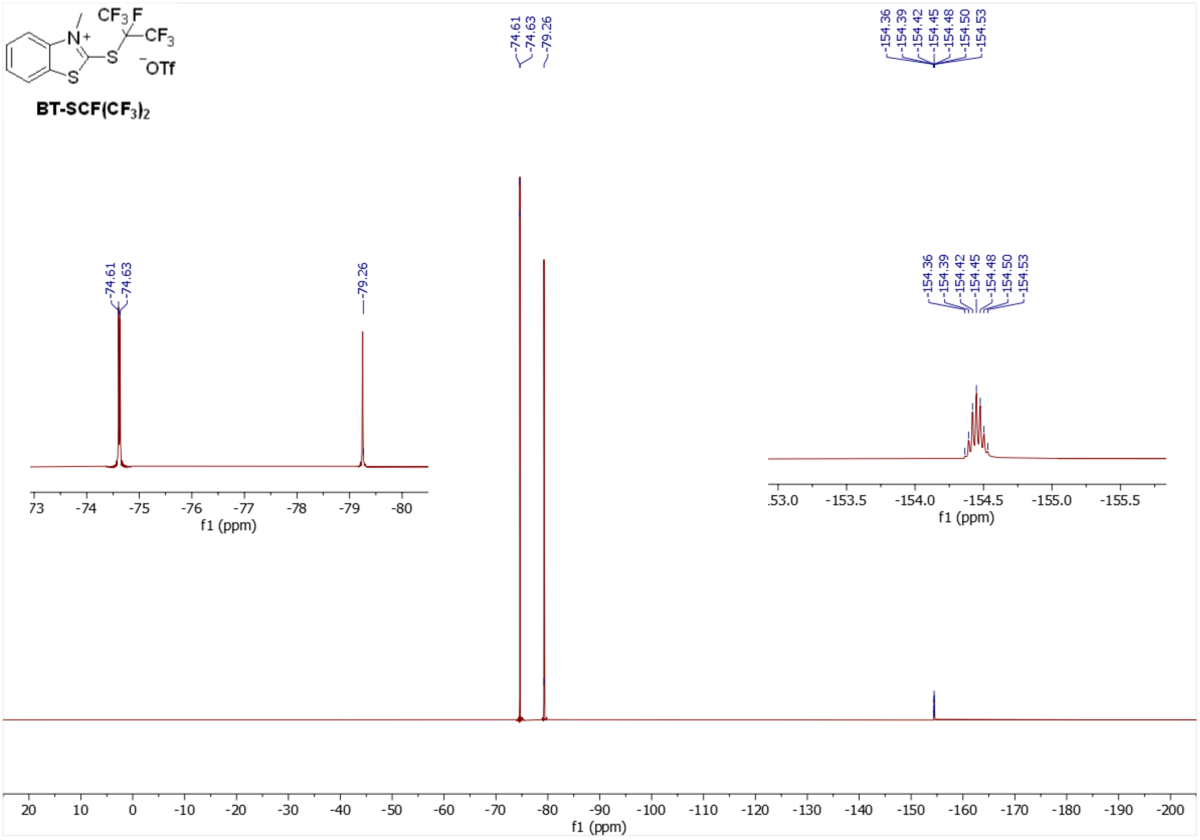
BT-SCF(CF3)2
-74.61
-74.63
-79.26
-154.36
-154.39
-154.42
-154.45
-154.48
-154.50
-154.53
f1 (ppm)

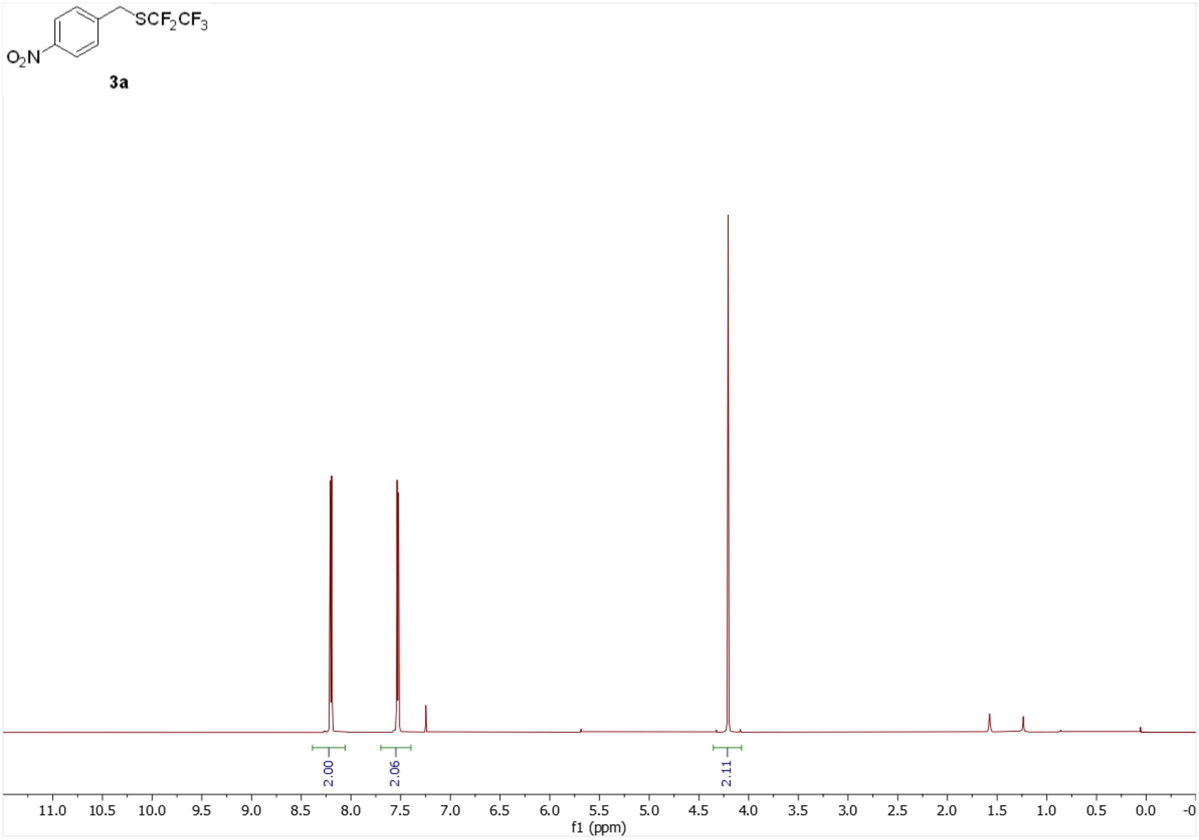
3a
2.00
2.06
2.11
f1 (ppm)

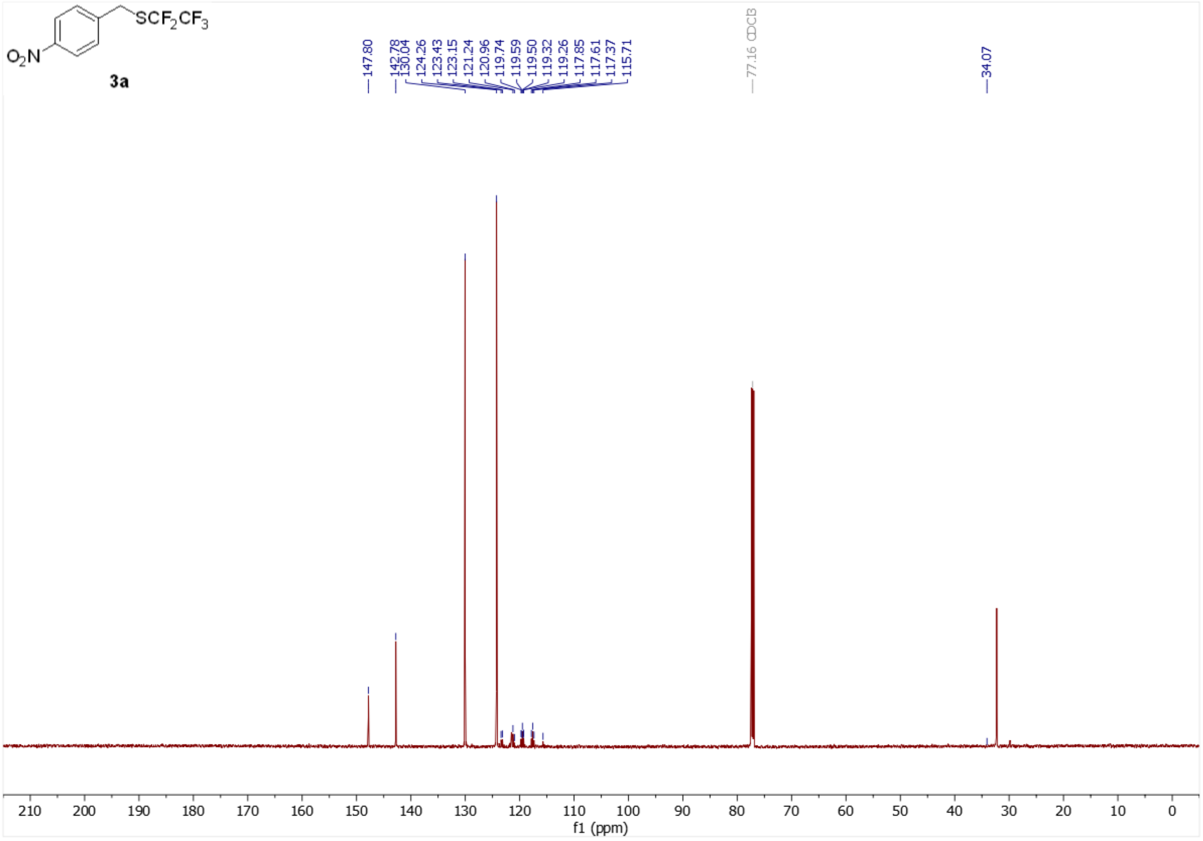

SCF2CF3
O2N
3a
147.80
142.78
130.04
124.26
123.43
123.15
121.24
120.96
119.74
119.59
119.50
119.32
119.26
117.85
117.61
117.37
115.71
77.16 CDCl3
34.07
210 200 190 180 170 160 150 140 130 120 110 100 90 80 70 60 50 40 30 20 10 0
f1 (ppm)

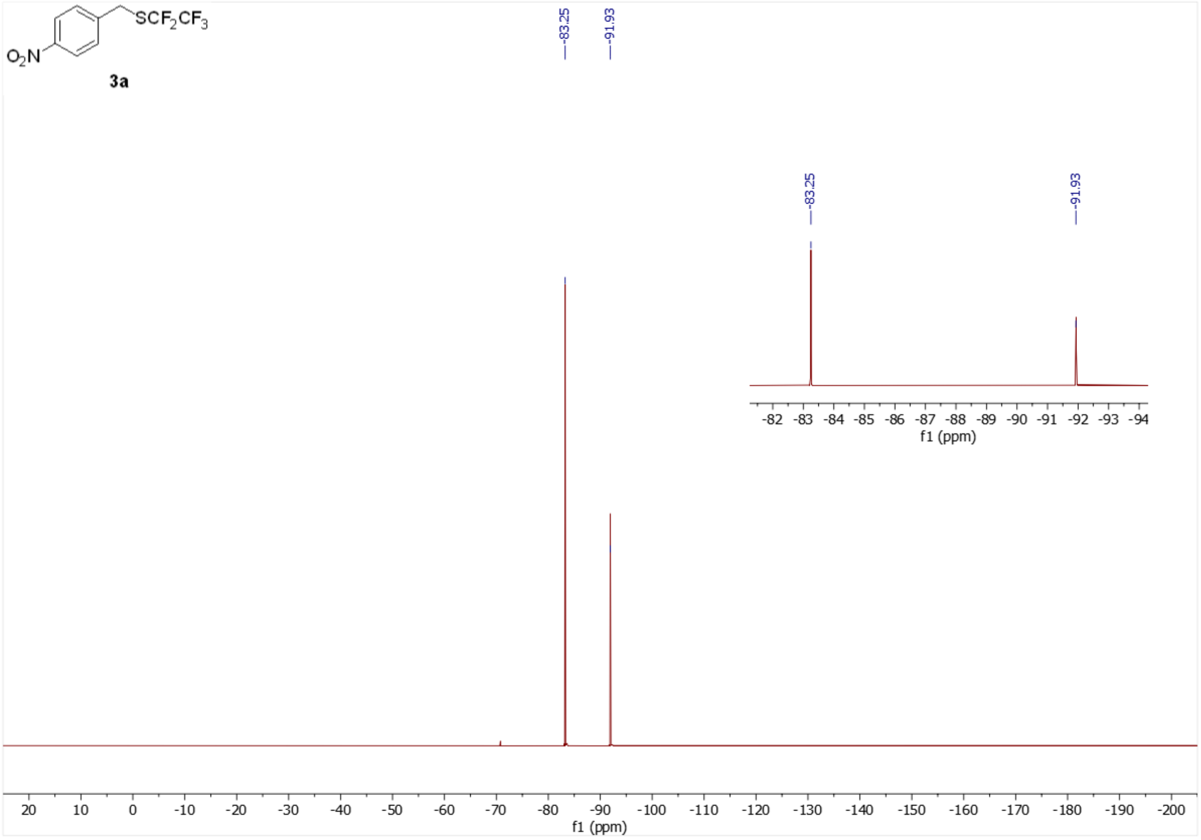

SCF2CF3
O2N
3a
-83.25
-91.93
-82 -83 -84 -85 -86 -87 -88 -89 -90 -91 -92 -93 -94
f1 (ppm)
20 10 0 -10 -20 -30 -40 -50 -60 -70 -80 -90 -100 -110 -120 -130 -140 -150 -160 -170 -180 -190 -200
f1 (ppm)

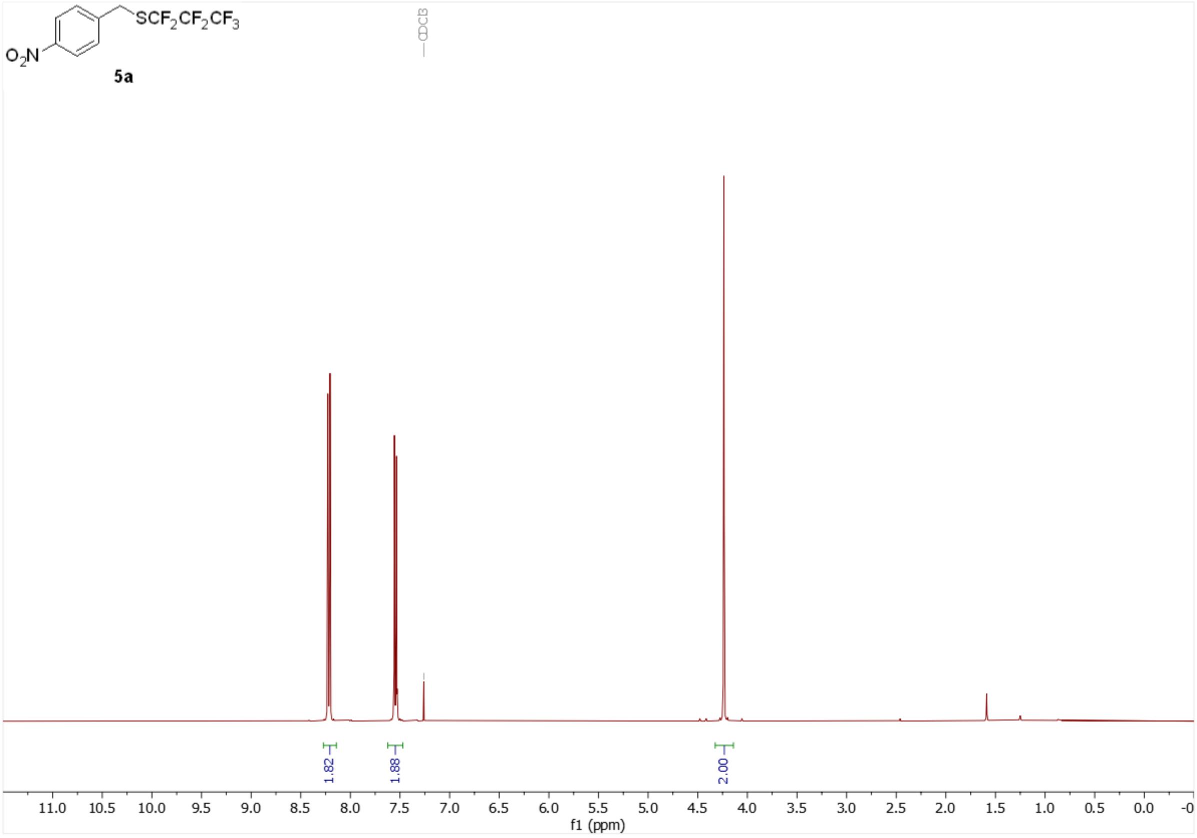
SCF2CF2CF3
O2N
5a
CDCl3
1.82
1.88
2.00
f1 (ppm)

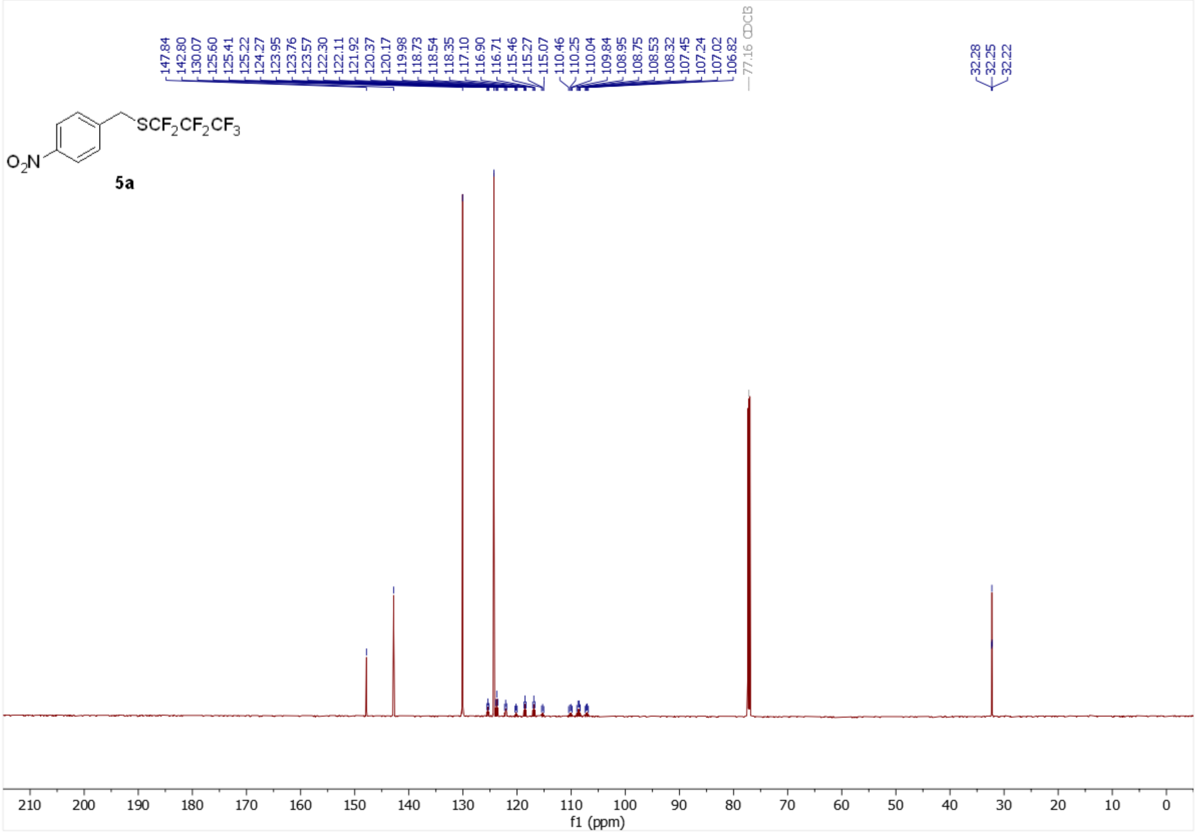
147.84
142.80
130.07
125.60
125.41
125.22
124.27
123.95
123.76
123.57
122.30
122.11
121.92
120.37
120.17
119.98
118.73
118.54
118.35
117.10
116.90
116.71
115.46
115.27
115.07
110.46
110.25
110.04
109.84
108.95
108.75
108.53
108.32
107.45
107.24
107.02
106.82
77.16 CDCl3
32.28
32.25
32.22
SCF2CF2CF3
O2N
5a
f1 (ppm)

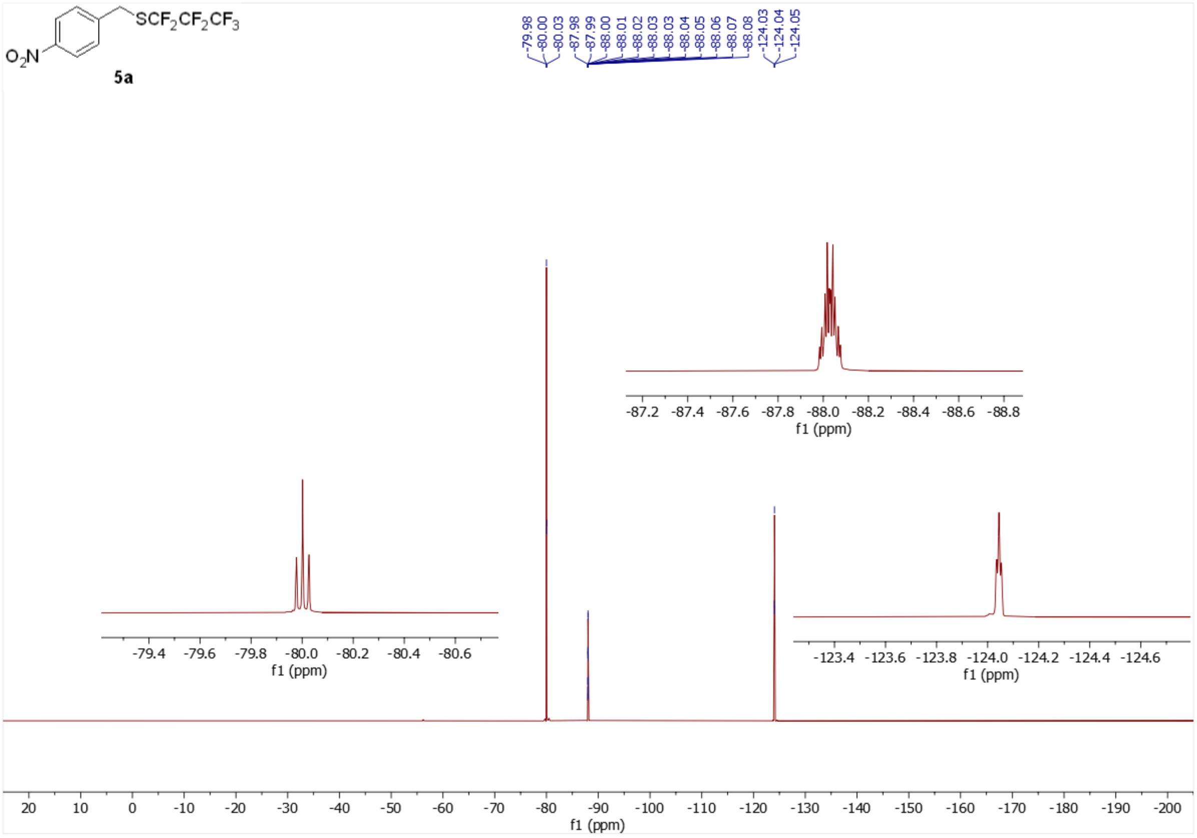
SCF2CF2CF3
O2N
5a
-79.98
-80.00
-80.03
-87.98
-87.99
-88.00
-88.01
-88.02
-88.03
-88.03
-88.04
-88.05
-88.06
-88.07
-88.08
-124.03
-124.04
-124.05
f1 (ppm)

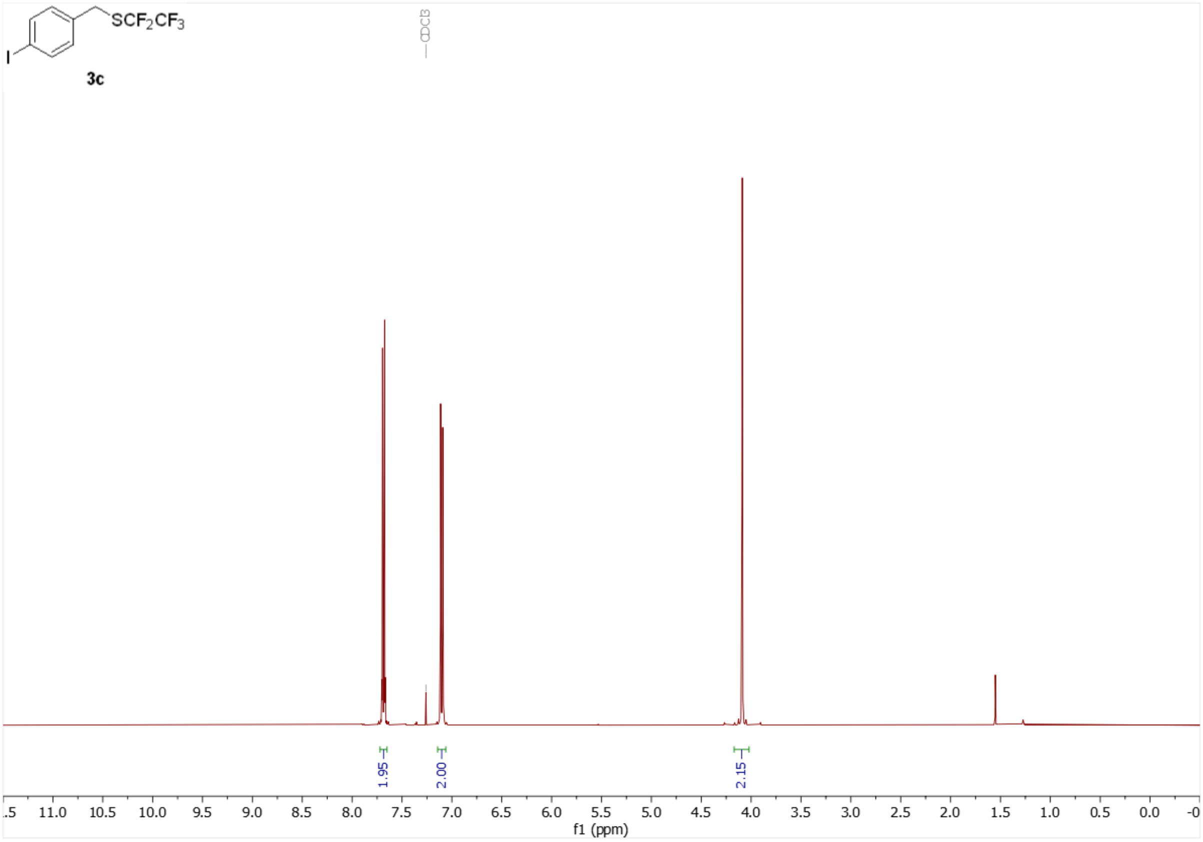
SCF2CF3
I
3c
CDCl3
1.95
2.00
2.15
f1 (ppm)

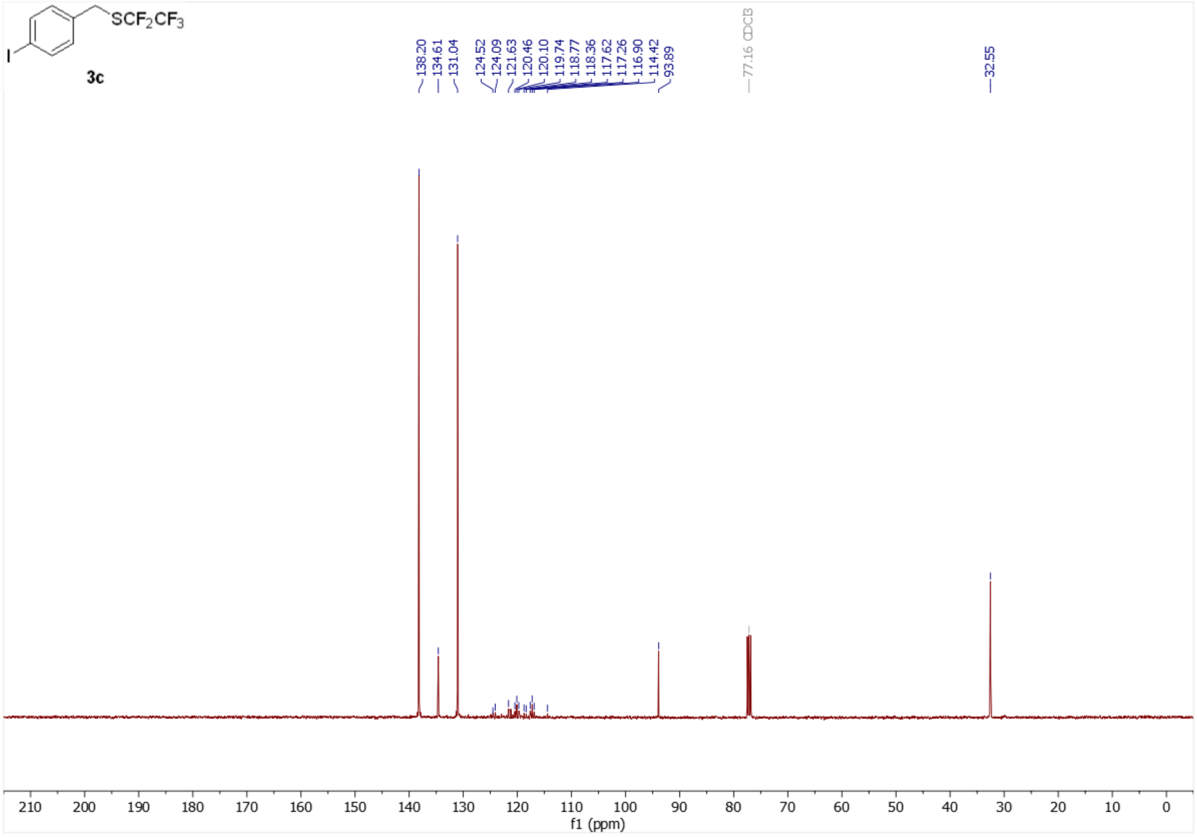
SCF2CF3
I
3c
138.20
134.61
131.04
124.52
124.09
121.63
120.46
120.10
119.74
118.77
118.36
117.62
117.26
116.90
114.42
93.89
77.16 CDCl3
32.55
f1 (ppm)

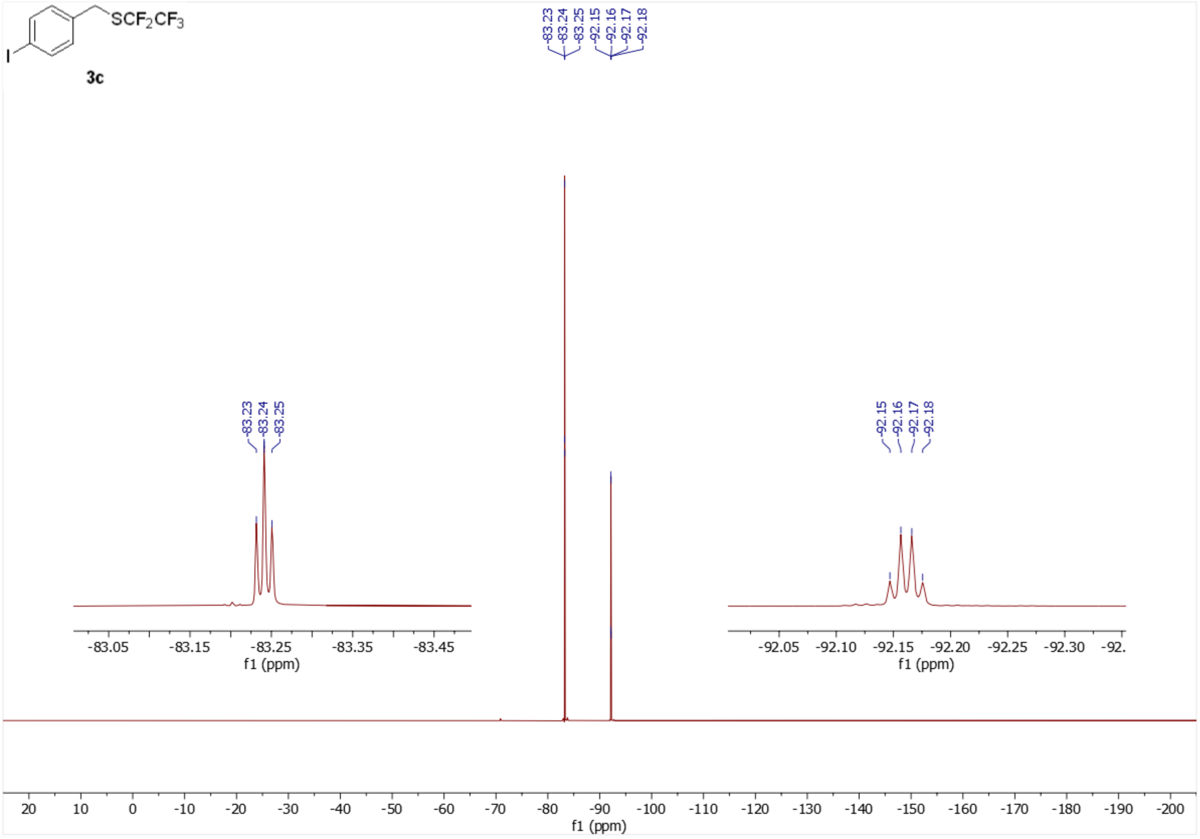
SCF2CF3
I
3c
-83.23
-83.24
-83.25
-92.15
-92.16
-92.17
-92.18
f1 (ppm)

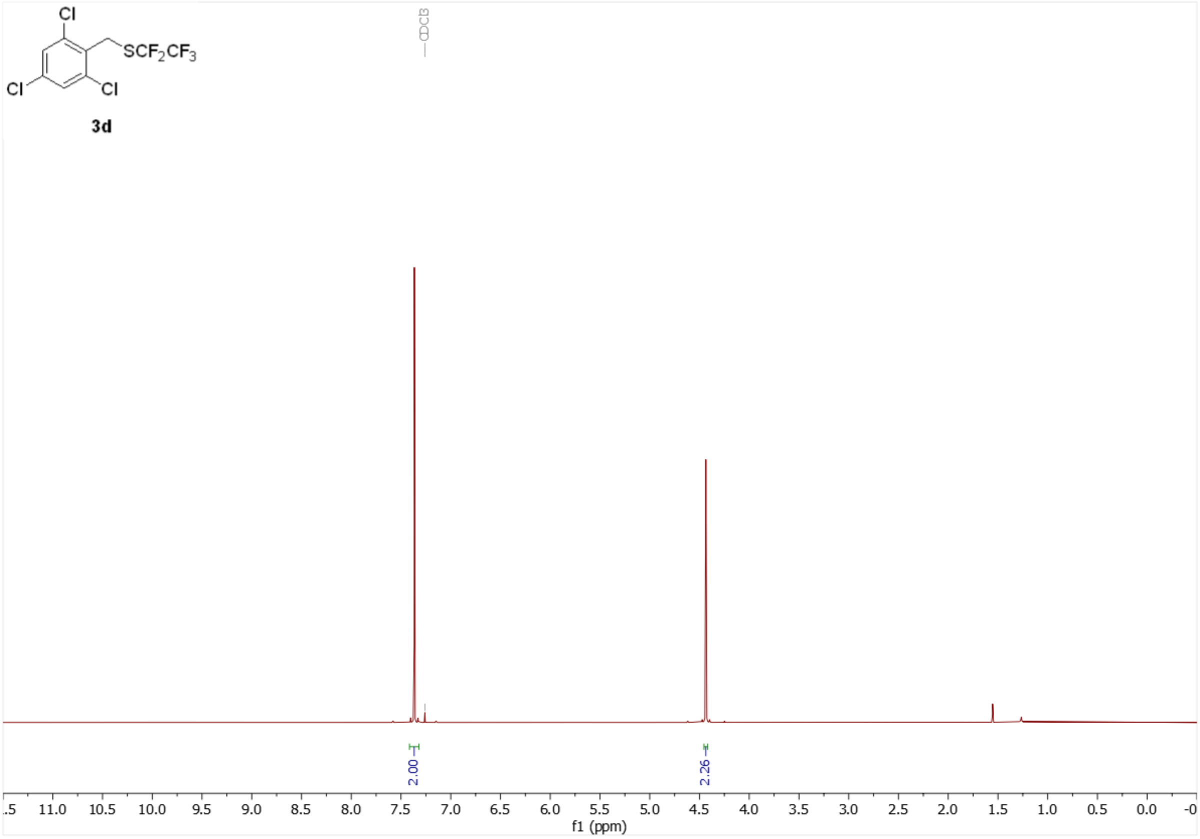

Cl
SCF2CF3
Cl
Cl
3d
CDCl3
2.00
2.26
f1 (ppm)

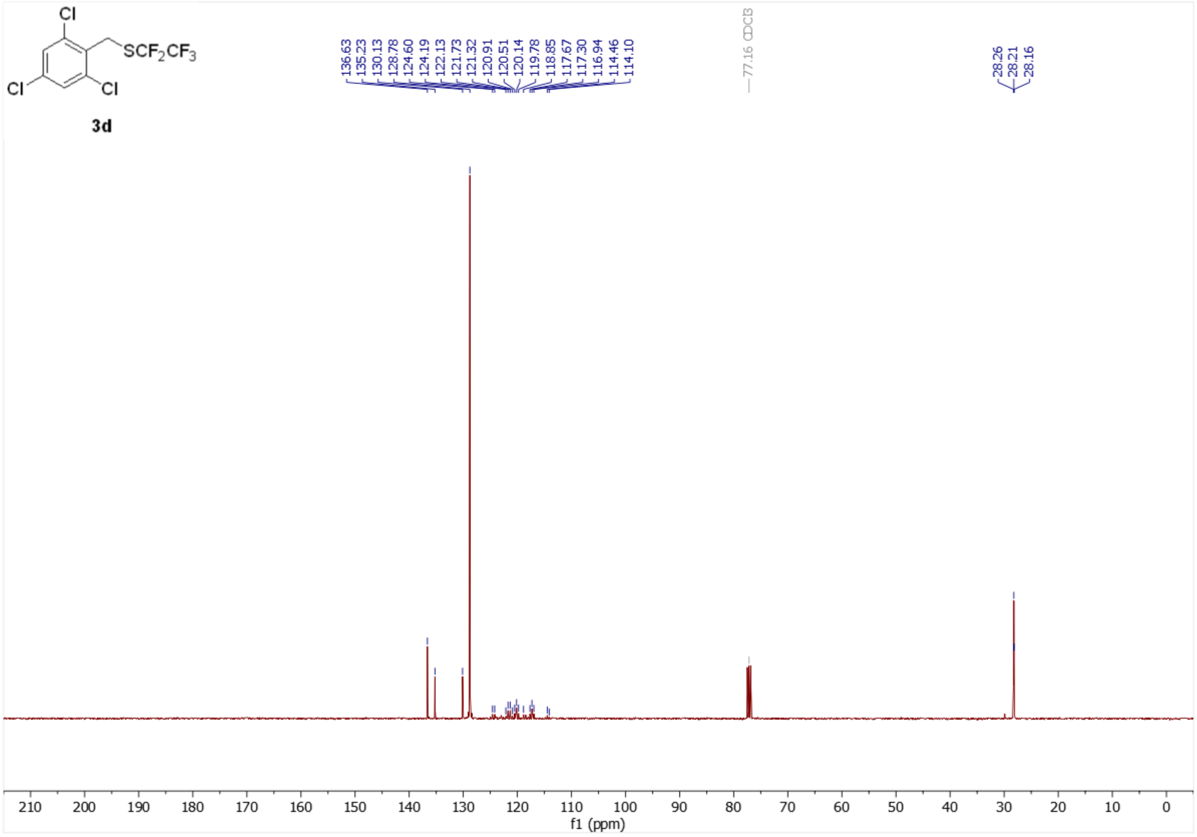

Cl
SCF2CF3
Cl
Cl
3d
136.63
135.23
130.13
128.78
124.60
124.19
122.13
121.73
121.32
120.91
120.51
120.14
119.78
118.85
117.67
117.30
116.94
114.46
114.10
77.16 CDCl3
28.26
28.21
28.16
f1 (ppm)

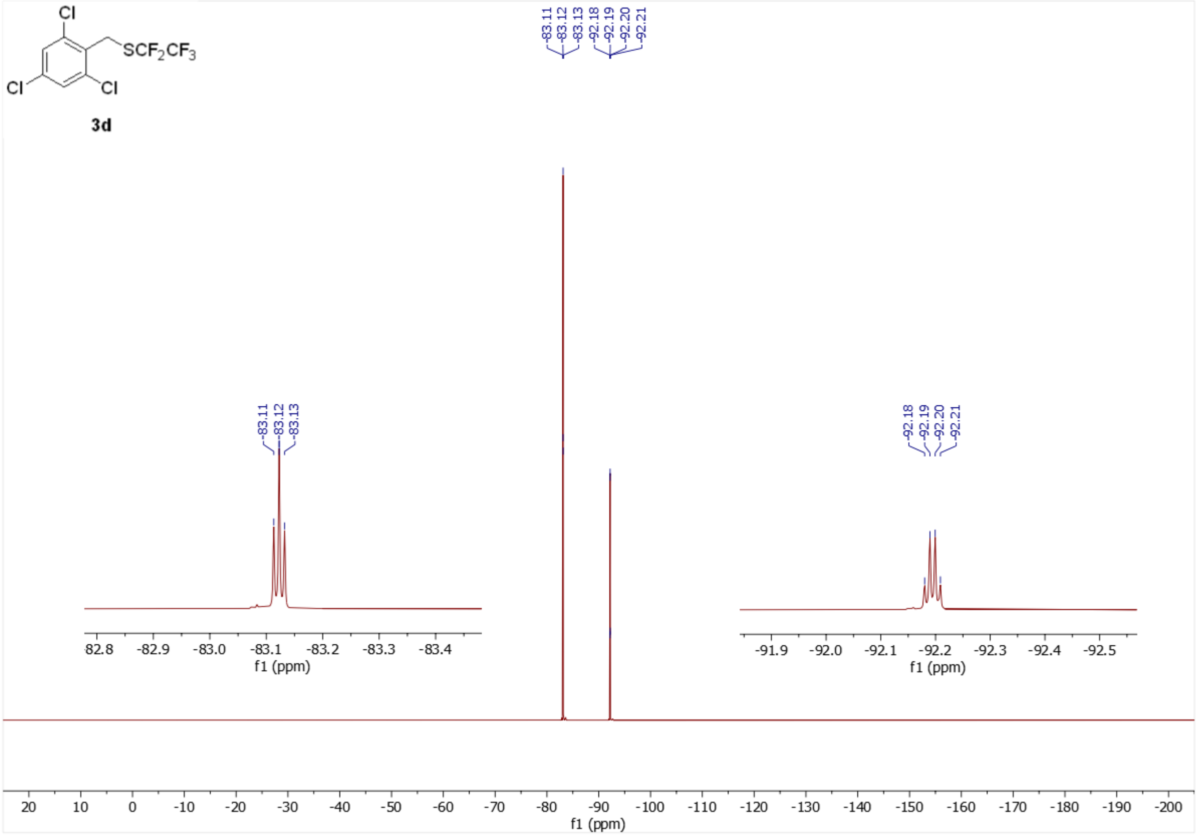
SCF2CF3
3d
-83.11
-83.12
-83.13
-92.18
-92.19
-92.20
-92.21
f1 (ppm)

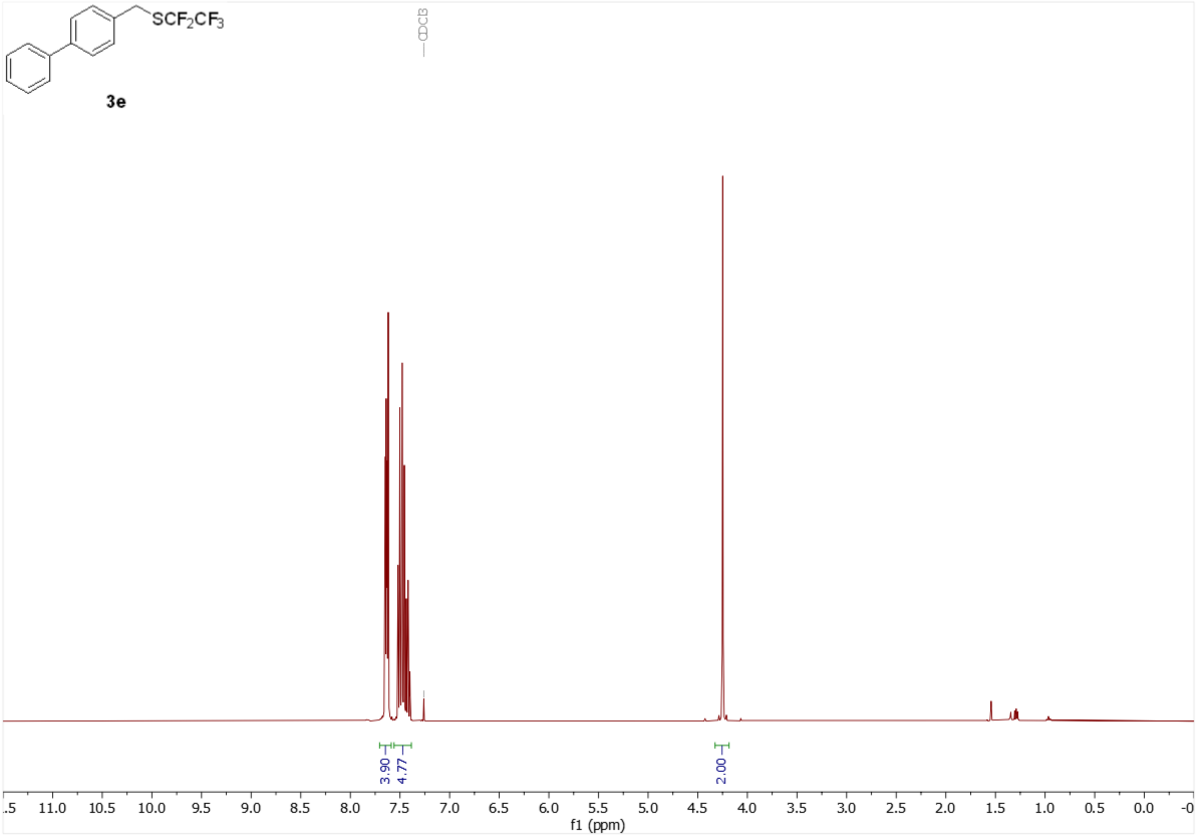
SCF2CF3
3e
CDCl3
3.90
4.77
2.00
f1 (ppm)

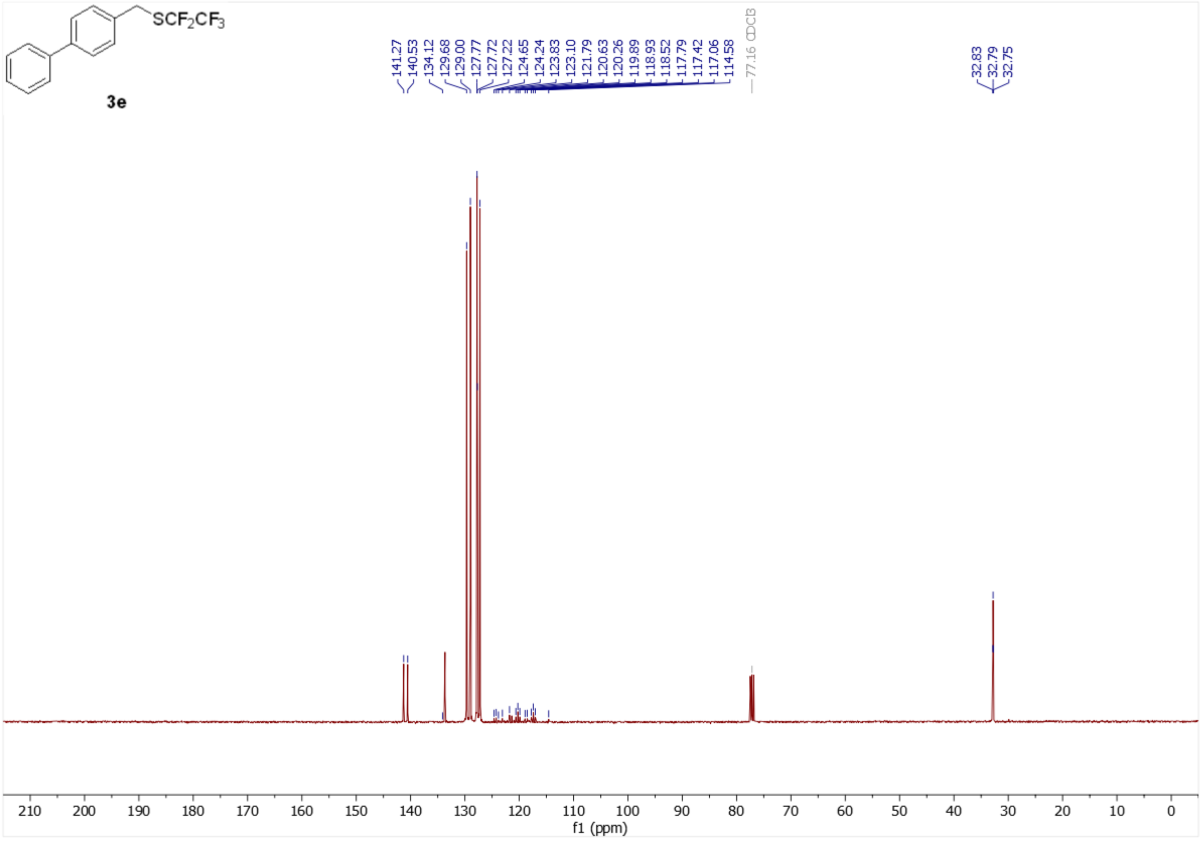
SCF2CF3
3e
141.27
140.53
134.12
129.68
129.00
127.77
127.72
127.22
124.65
124.24
123.83
123.10
121.79
120.63
120.26
119.89
118.93
118.52
117.79
117.42
117.06
114.58
77.16 CDCl3
32.83
32.79
32.75
f1 (ppm)

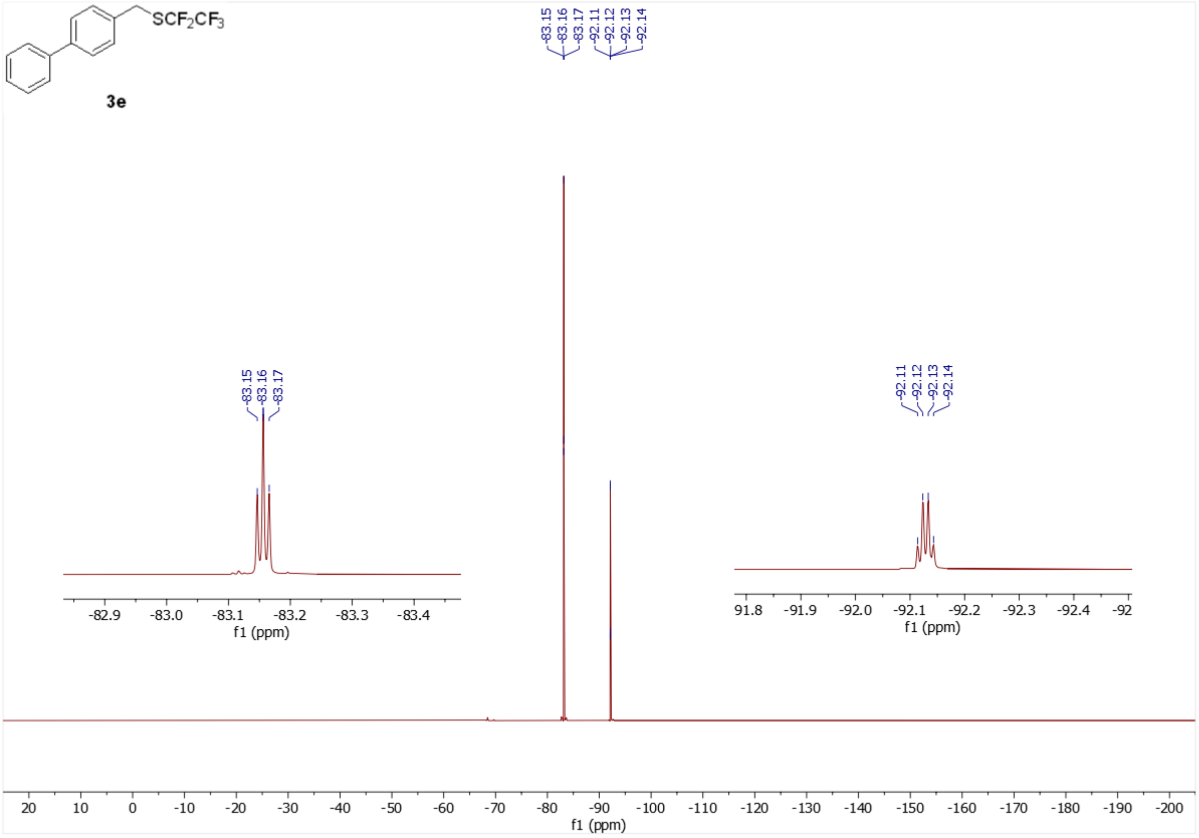
SCF2CF3
3e
-83.15
-83.16
-83.17
-92.11
-92.12
-92.13
-92.14
f1 (ppm)

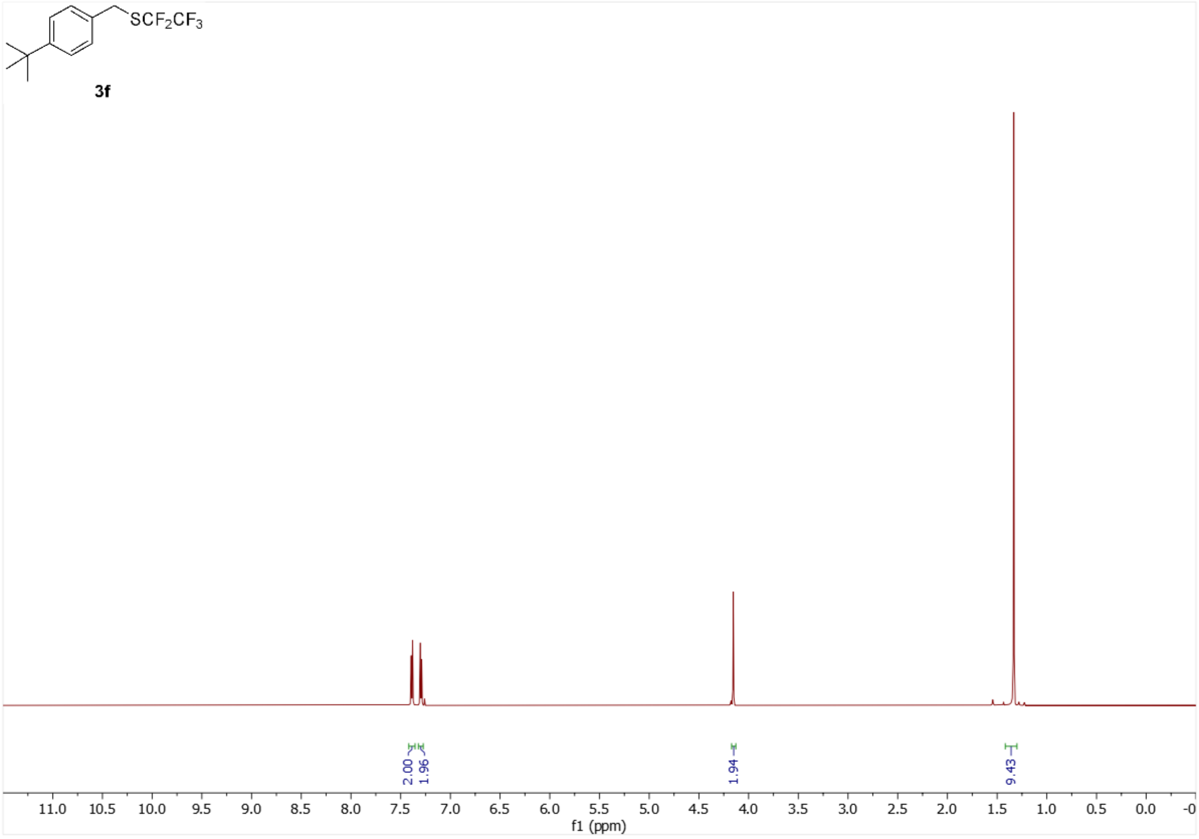
SCF2CF3
3f
2.00
1.96
1.94
9.43
11.0 10.5 10.0 9.5 9.0 8.5 8.0 7.5 7.0 6.5 6.0 5.5 5.0 4.5 4.0 3.5 3.0 2.5 2.0 1.5 1.0 0.5 0.0 -0
f1 (ppm)

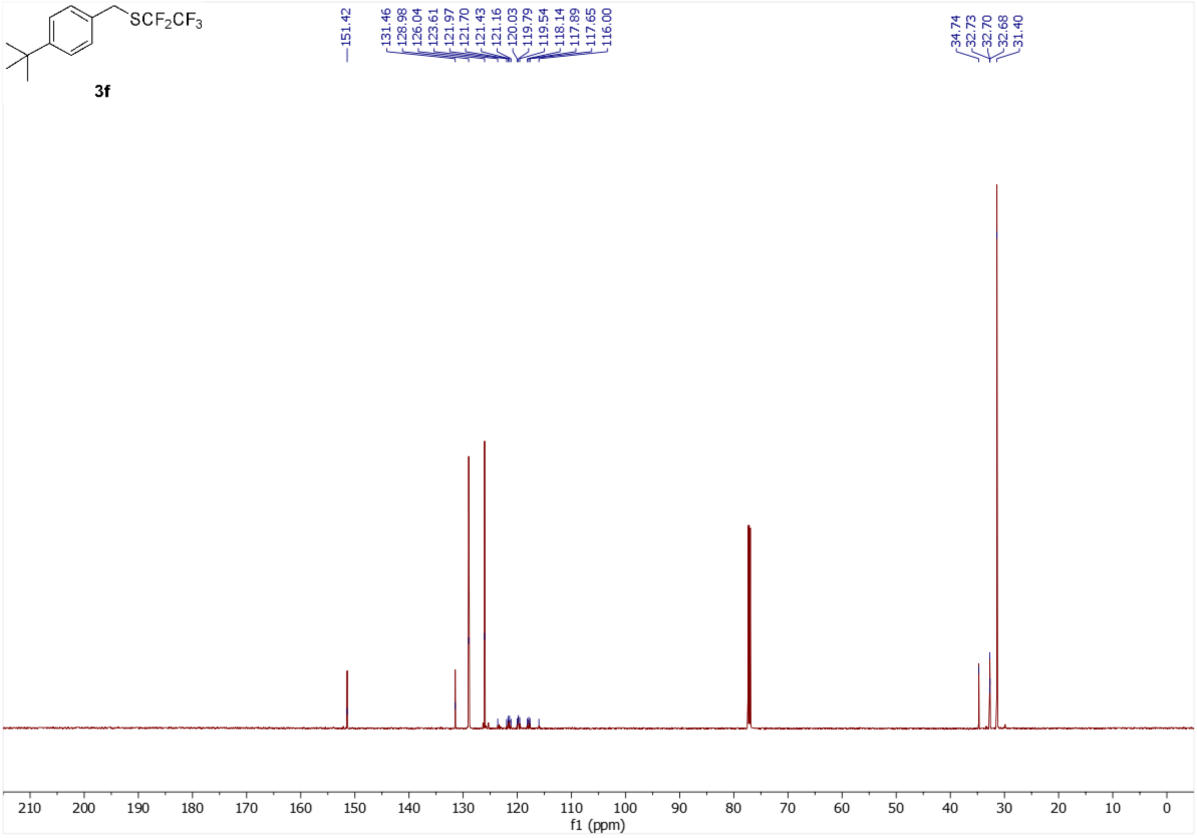
SCF2CF3
3f
151.42
131.46
128.98
126.04
123.61
121.97
121.70
121.43
121.16
120.03
119.79
119.54
118.14
117.89
117.65
116.00
34.74
32.73
32.70
32.68
31.40
210 200 190 180 170 160 150 140 130 120 110 100 90 80 70 60 50 40 30 20 10 0
f1 (ppm)

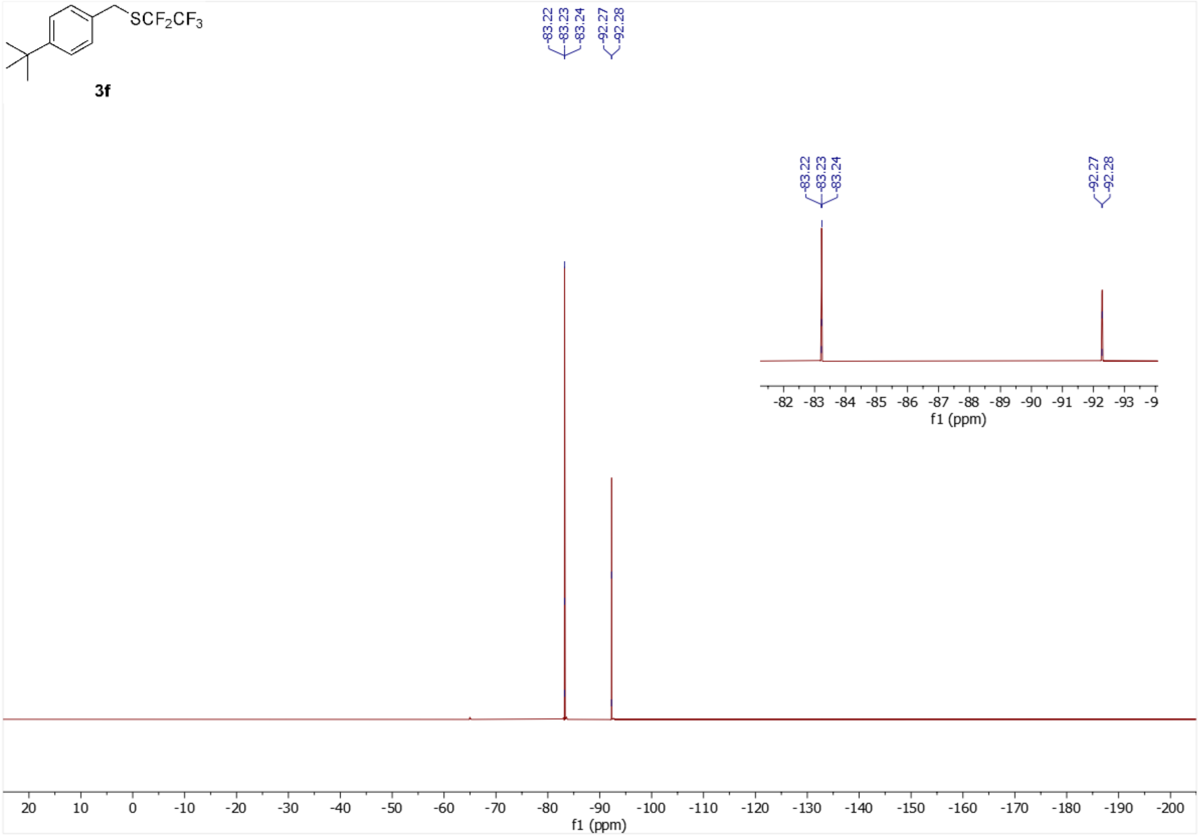
SCF2CF3
3f
-83.22
-83.23
-83.24
-92.27
-92.28
f1 (ppm)

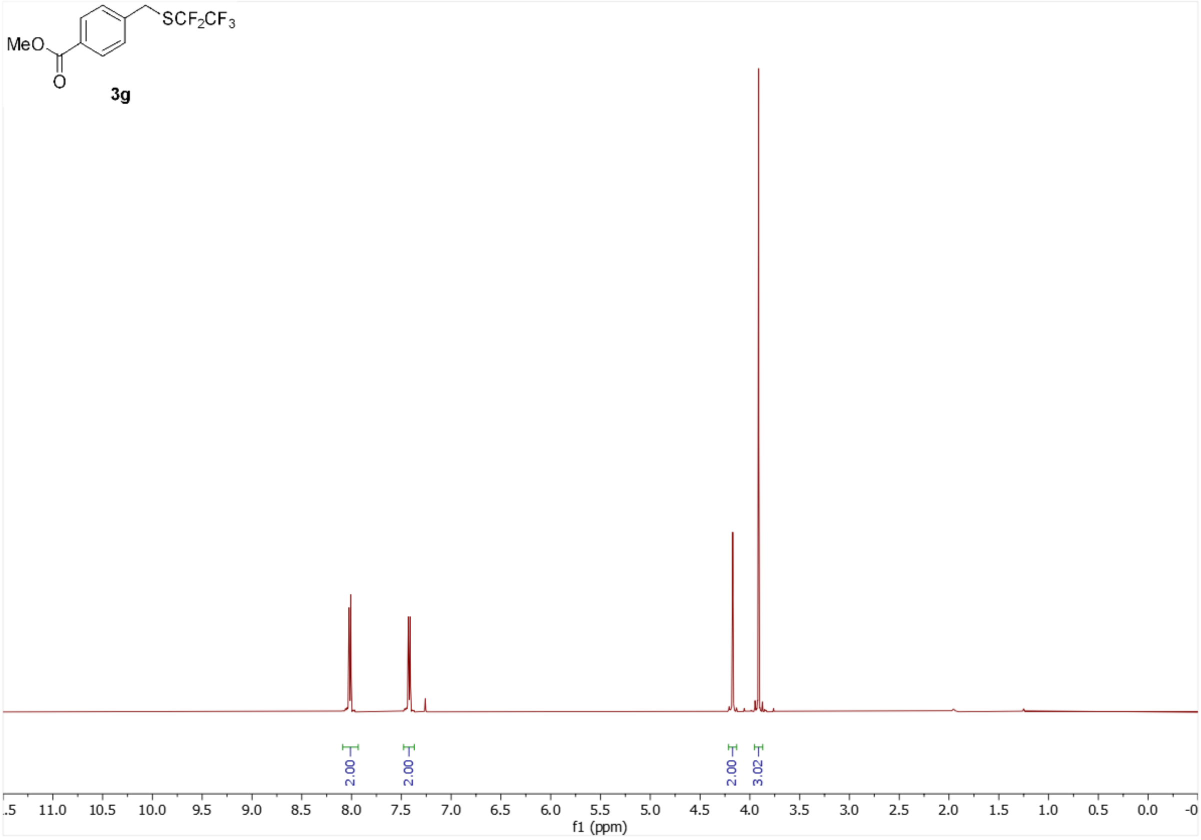
SCF2CF3
MeO
O
3g
2.00
2.00
2.00
3.02
f1 (ppm)

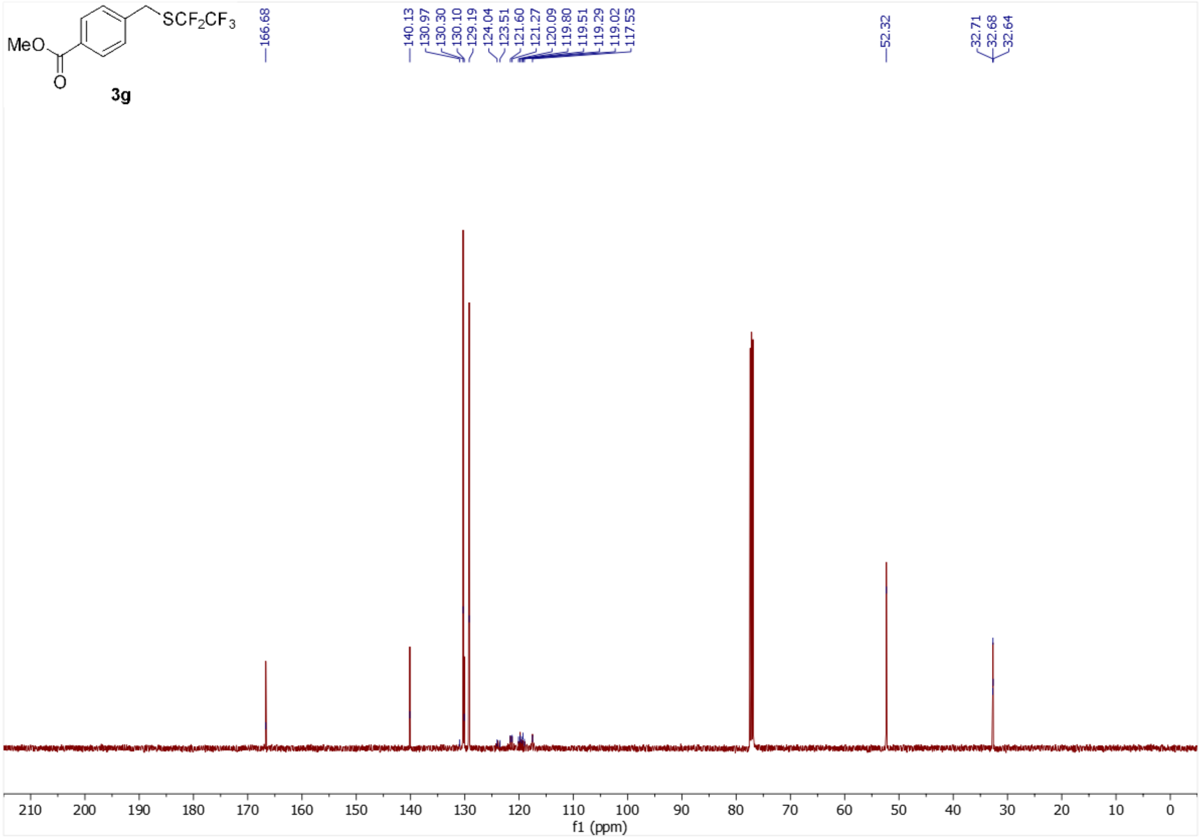
SCF2CF3
MeO
O
3g
166.68
140.13
130.97
130.30
130.10
129.19
124.04
123.51
121.60
121.27
120.09
119.80
119.51
119.29
119.02
117.53
52.32
32.71
32.68
32.64
210 200 190 180 170 160 150 140 130 120 110 100 90 80 70 60 50 40 30 20 10 0
f1 (ppm)

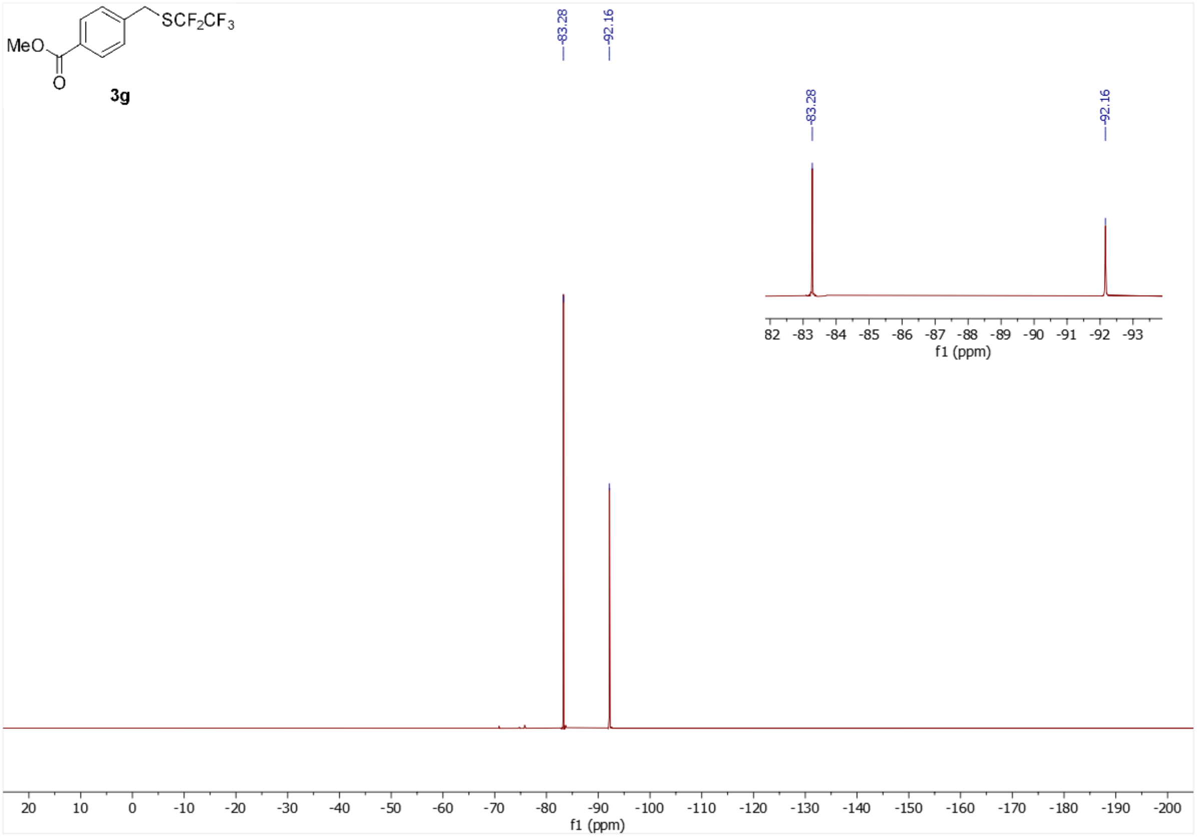
SCF2CF3
MeO
O
3g
-83.28
-92.16
82 -83 -84 -85 -86 -87 -88 -89 -90 -91 -92 -93
f1 (ppm)
20 10 0 -10 -20 -30 -40 -50 -60 -70 -80 -90 -100 -110 -120 -130 -140 -150 -160 -170 -180 -190 -200
f1 (ppm)

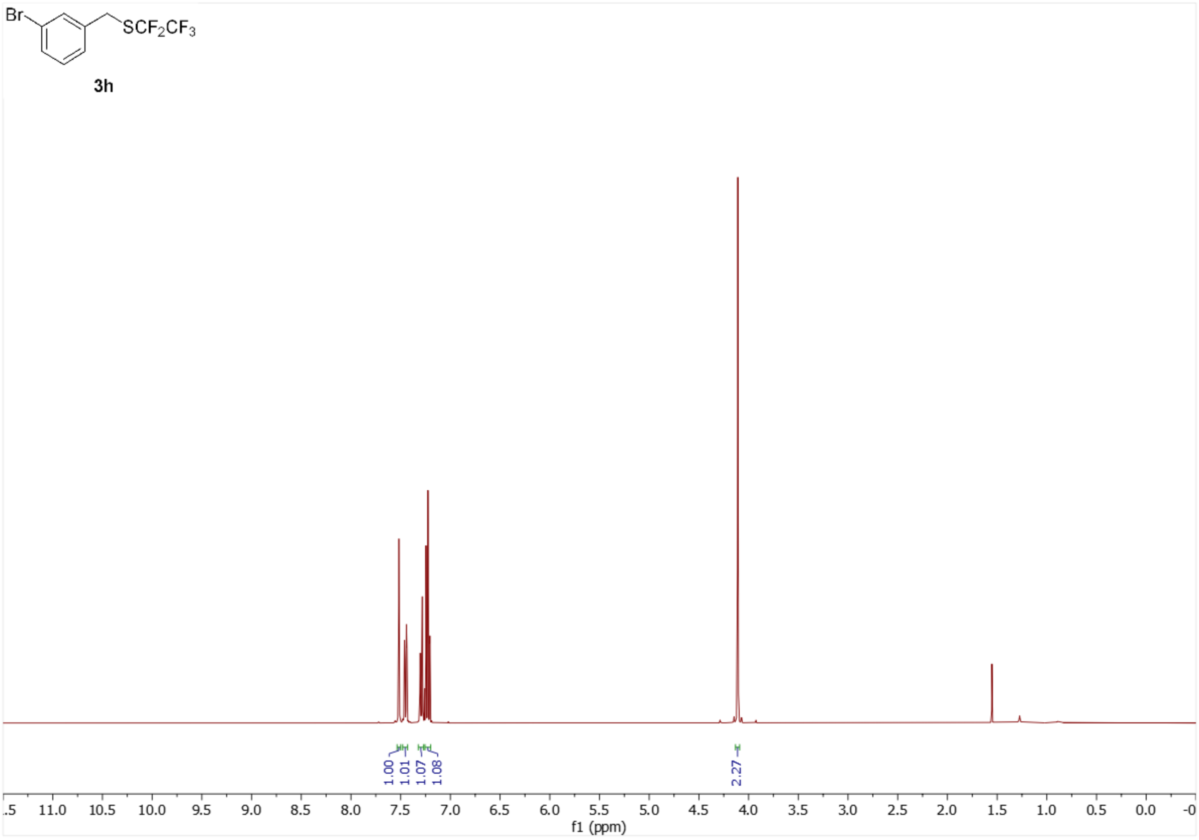
Br
SCF2CF3
3h
1.00
1.01
1.07
1.08
2.27
.5 11.0 10.5 10.0 9.5 9.0 8.5 8.0 7.5 7.0 6.5 6.0 5.5 5.0 4.5 4.0 3.5 3.0 2.5 2.0 1.5 1.0 0.5 0.0 -0
f1 (ppm)

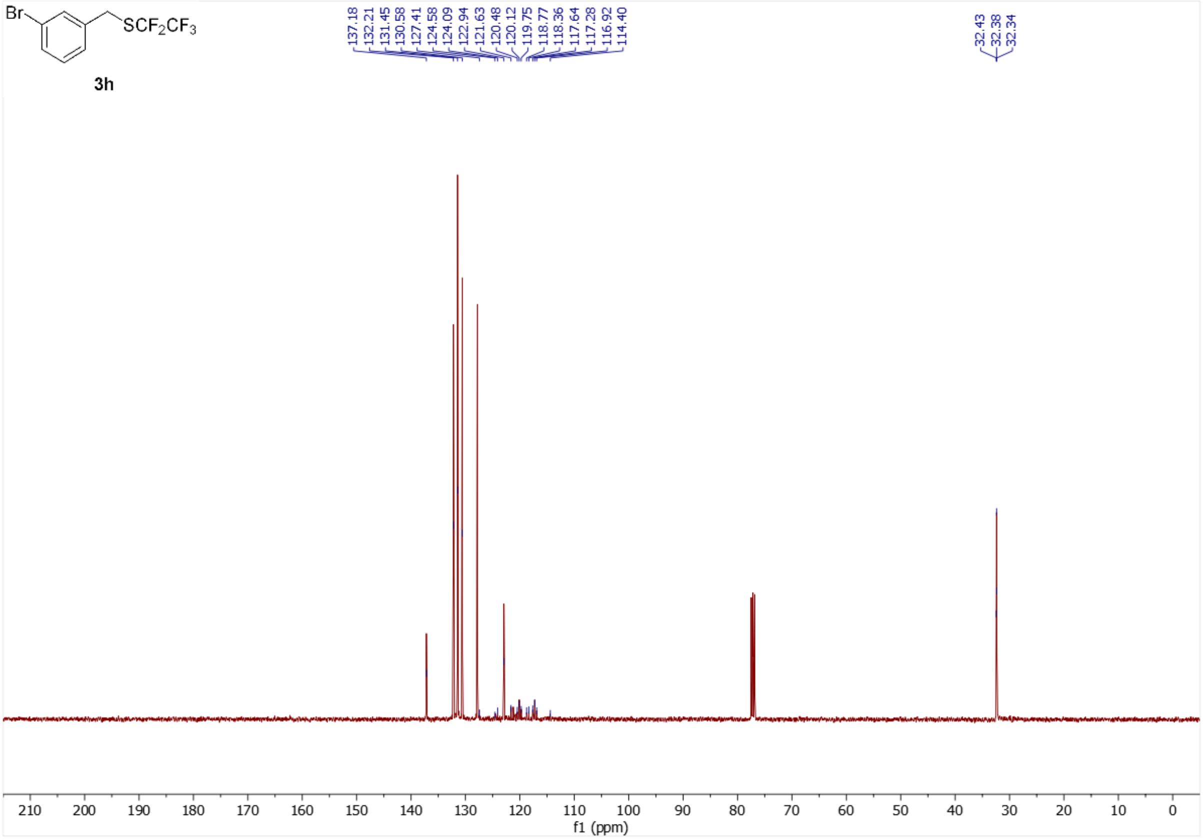
Br
SCF2CF3
3h
137.18
132.21
131.45
130.58
127.41
124.58
124.09
122.94
121.63
120.48
120.12
119.75
118.77
118.36
117.64
117.28
116.92
114.40
32.43
32.38
32.34
210 200 190 180 170 160 150 140 130 120 110 100 90 80 70 60 50 40 30 20 10 0
f1 (ppm)

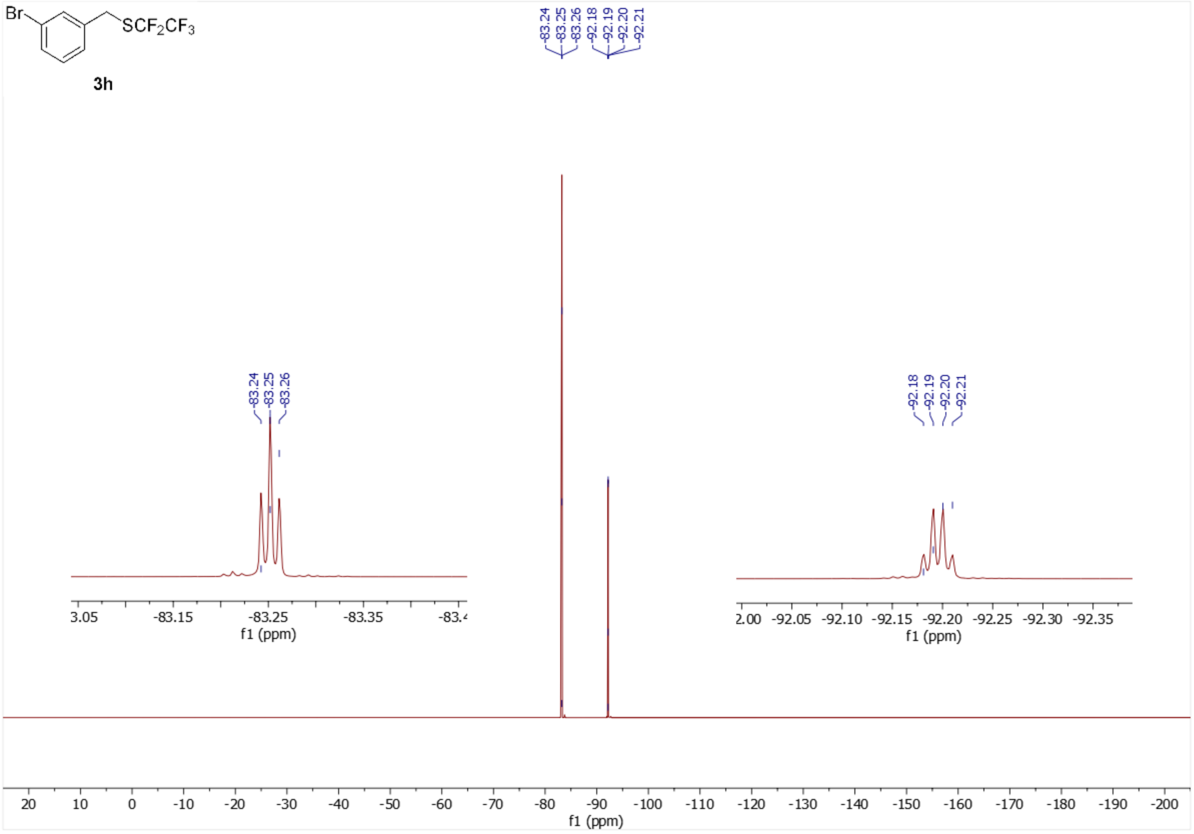
Br
SCF2CF3
3h
-83.24
-83.25
-83.26
-92.18
-92.19
-92.20
-92.21
f1 (ppm)

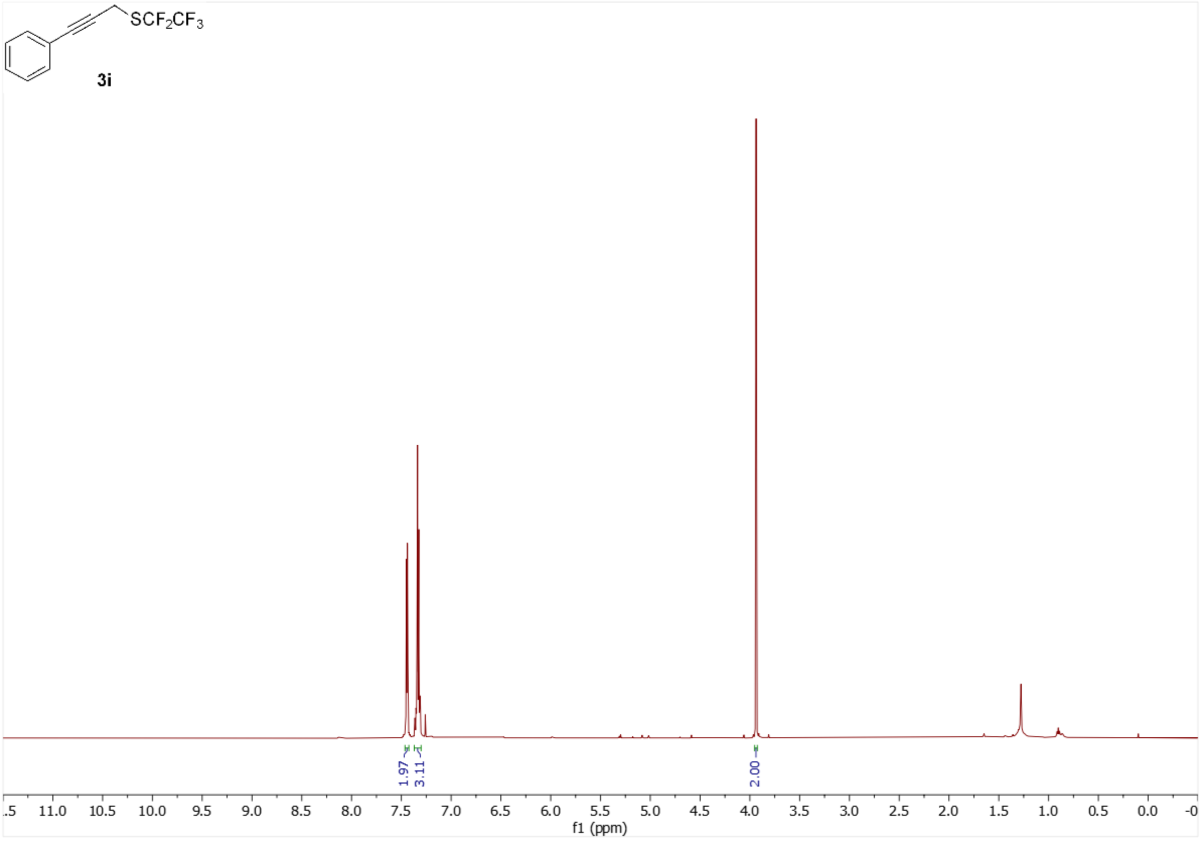
SCF2CF3
3i
1.97
3.11
2.00
f1 (ppm)

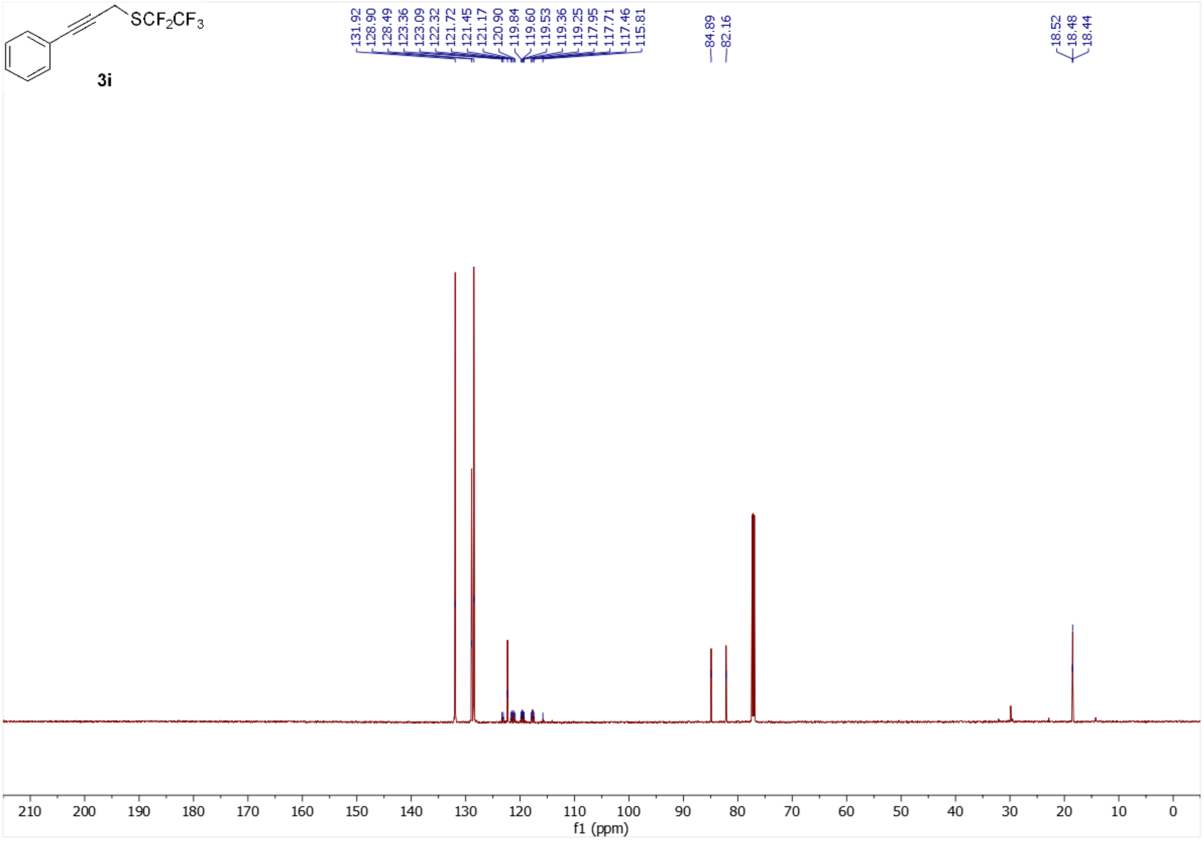
SCF2CF3
3i
131.92
128.90
128.49
123.36
123.09
122.32
121.72
121.45
121.17
120.90
119.84
119.60
119.53
119.36
119.25
117.95
117.71
117.46
115.81
84.89
82.16
18.52
18.48
18.44
210 200 190 180 170 160 150 140 130 120 110 100 90 80 70 60 50 40 30 20 10 0
f1 (ppm)

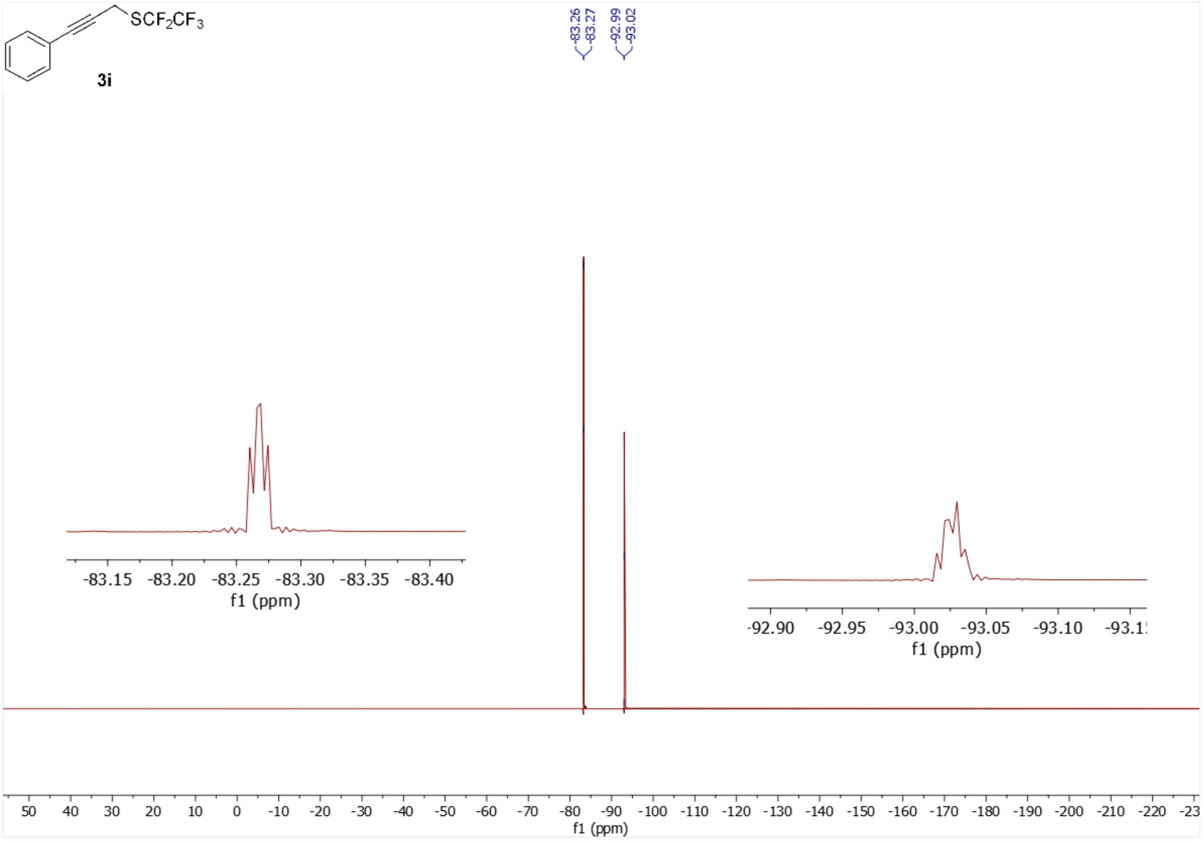
SCF2CF3
3i
-83.26
-83.27
-92.99
-93.02
-83.15 -83.20 -83.25 -83.30 -83.35 -83.40
f1 (ppm)
-92.90 -92.95 -93.00 -93.05 -93.10
f1 (ppm)
50 40 30 20 10 0 -10 -20 -30 -40 -50 -60 -70 -80 -90 -100 -110 -120 -130 -140 -150 -160 -170 -180 -190 -200 -210 -220
f1 (ppm)

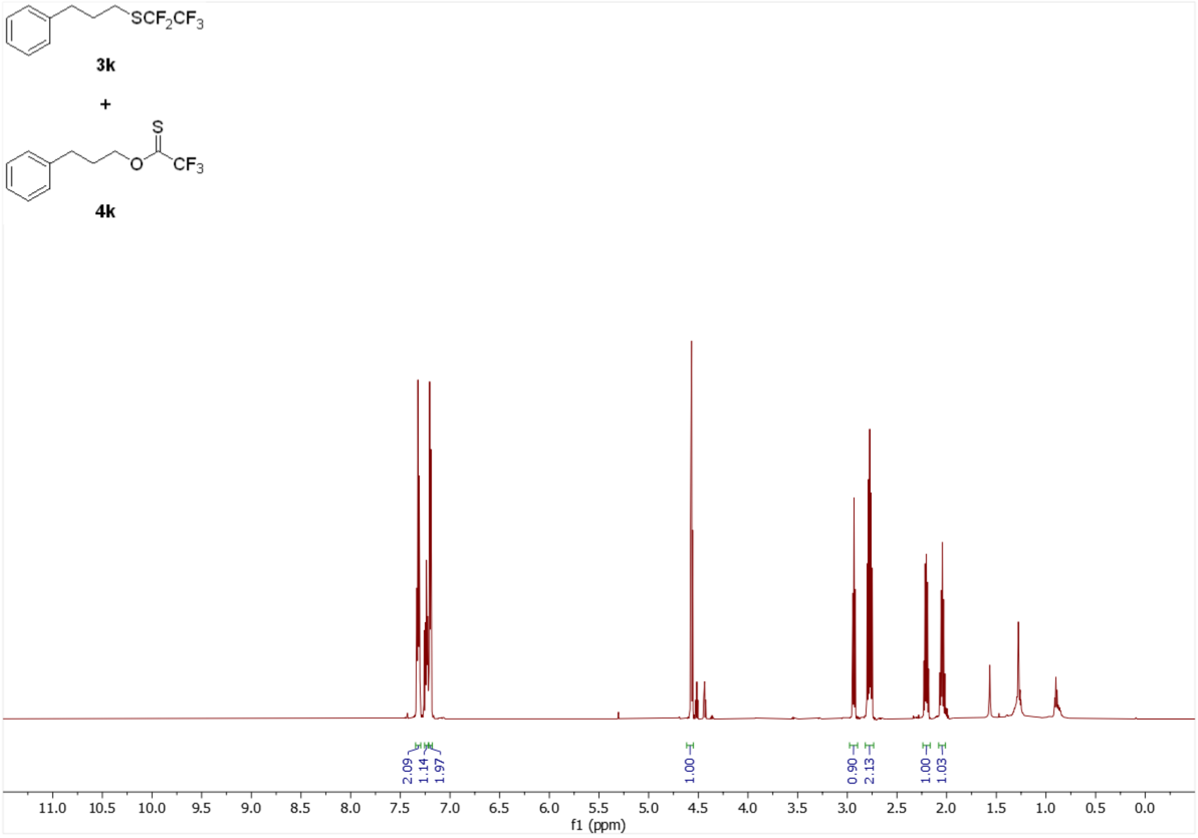
SCF2CF3
3k
+
S
O
CF3
4k
2.09
1.14
1.97
1.00
0.90
2.13
1.00
1.03
11.0 10.5 10.0 9.5 9.0 8.5 8.0 7.5 7.0 6.5 6.0 5.5 5.0 4.5 4.0 3.5 3.0 2.5 2.0 1.5 1.0 0.5 0.0
f1 (ppm)

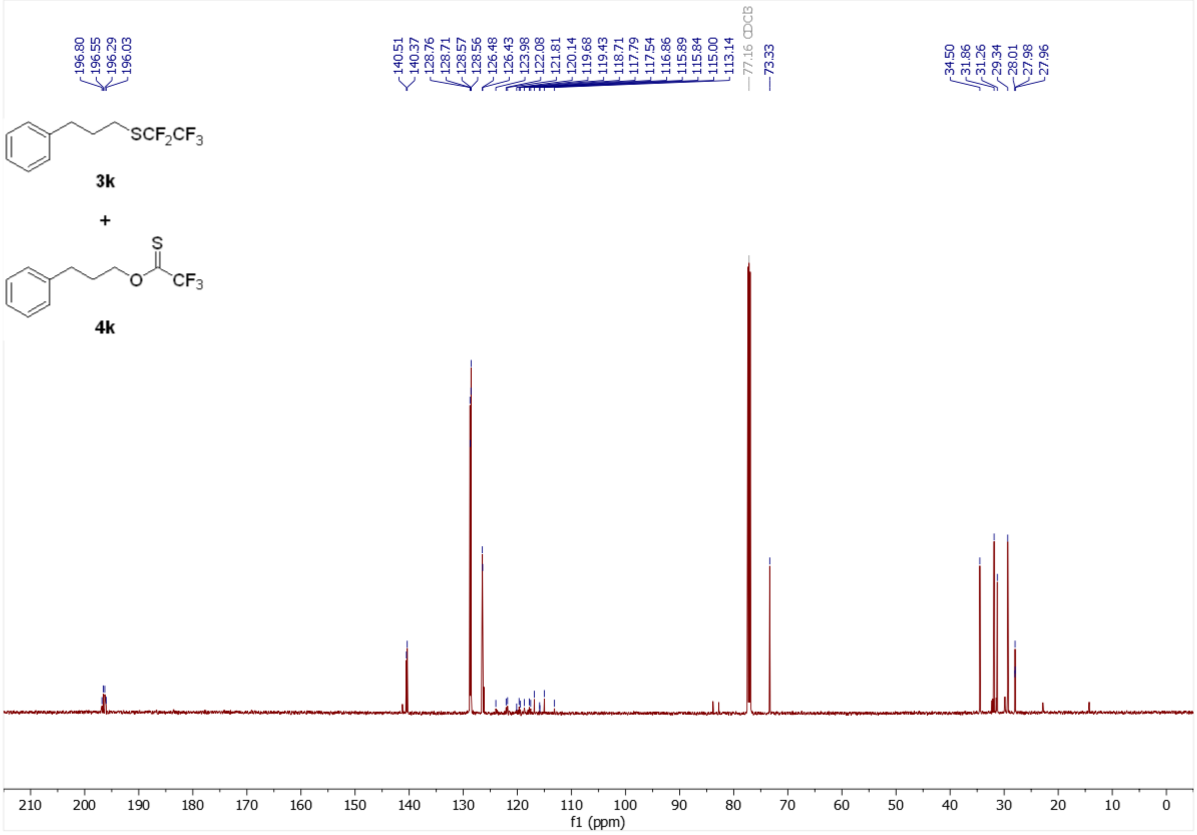
196.80
196.55
196.29
196.03
140.51
140.37
128.76
128.71
128.57
128.56
126.48
126.43
123.98
122.08
121.81
120.14
119.68
119.43
118.71
117.79
117.54
116.86
115.89
115.84
115.00
113.14
77.16 CDCl3
73.33
34.50
31.86
31.26
29.34
28.01
27.98
27.96
SCF2CF3
3k
+
S
O
CF3
4k
210 200 190 180 170 160 150 140 130 120 110 100 90 80 70 60 50 40 30 20 10 0
f1 (ppm)

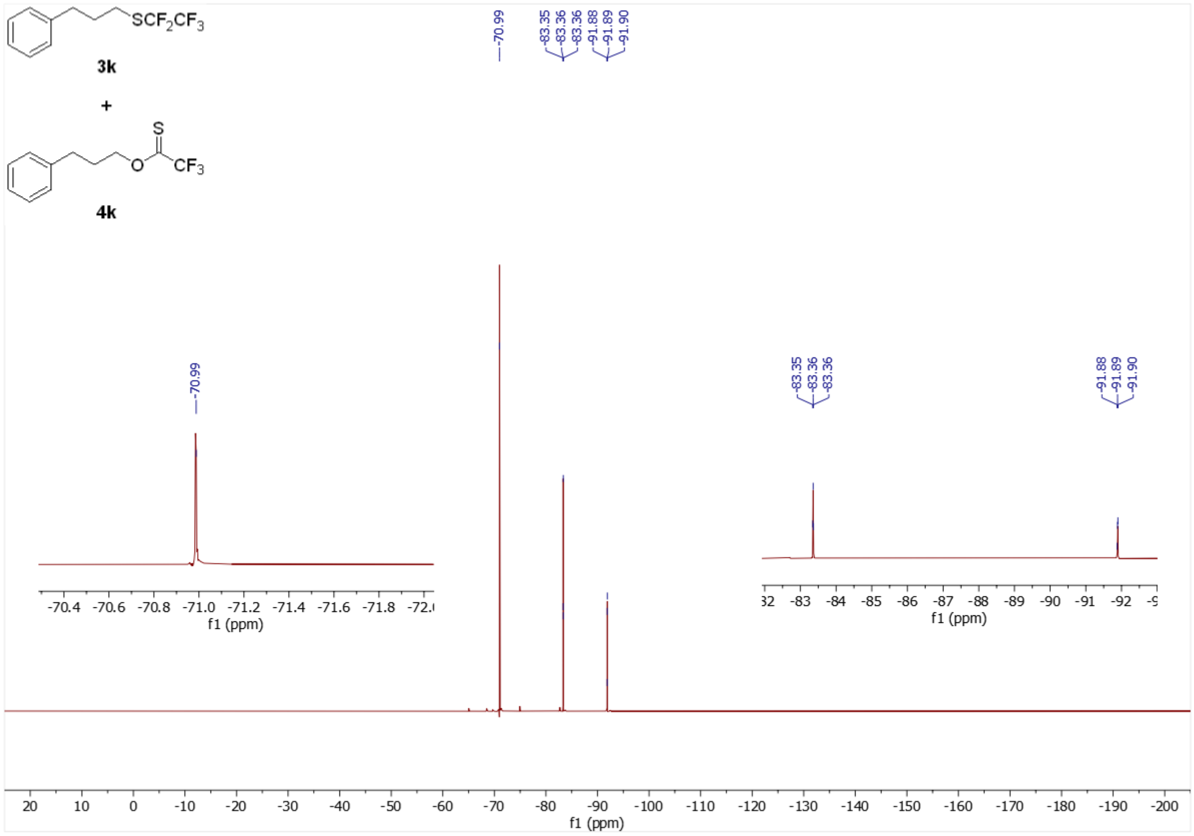
SCF2CF3
3k
+
S
O
CF3
4k
-70.99
-83.35
-83.36
-83.36
-91.88
-91.89
-91.90
f1 (ppm)

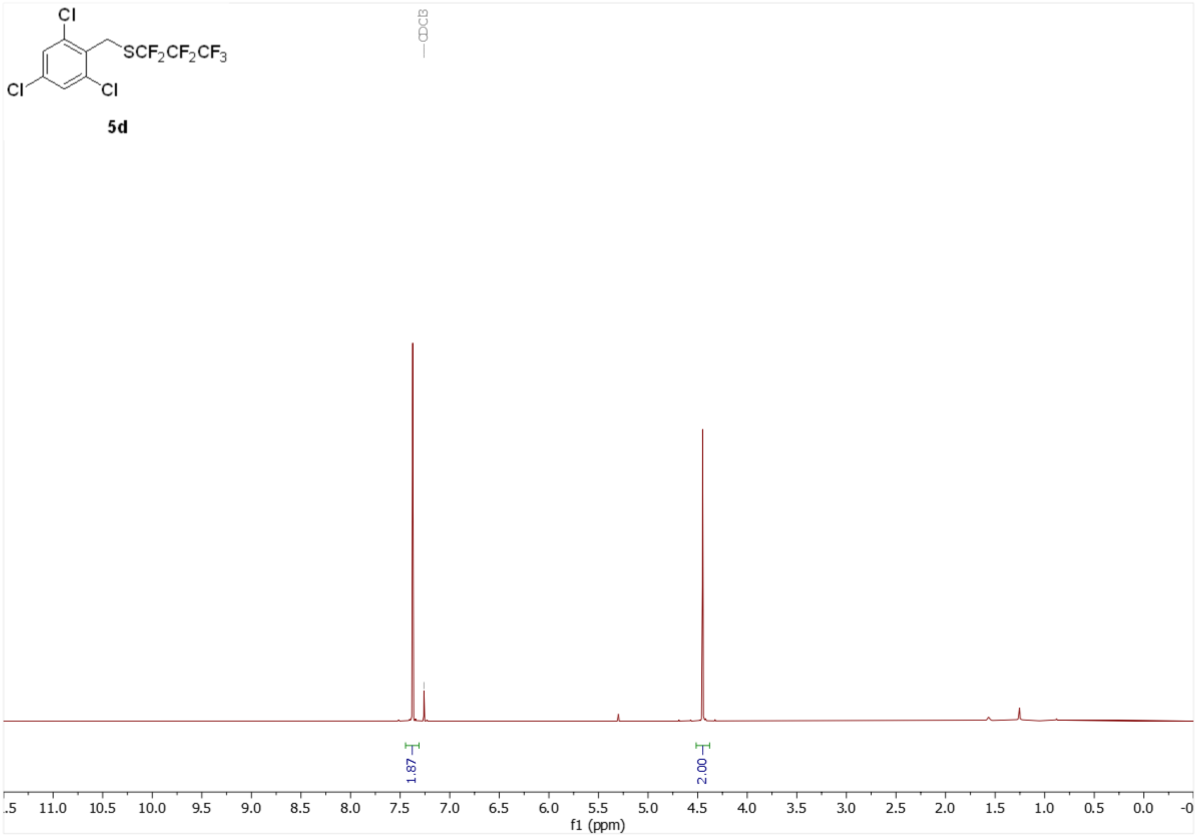
Cl
SCF2CF2CF3
Cl
Cl
5d
CDCl3
1.87
2.00
f1 (ppm)

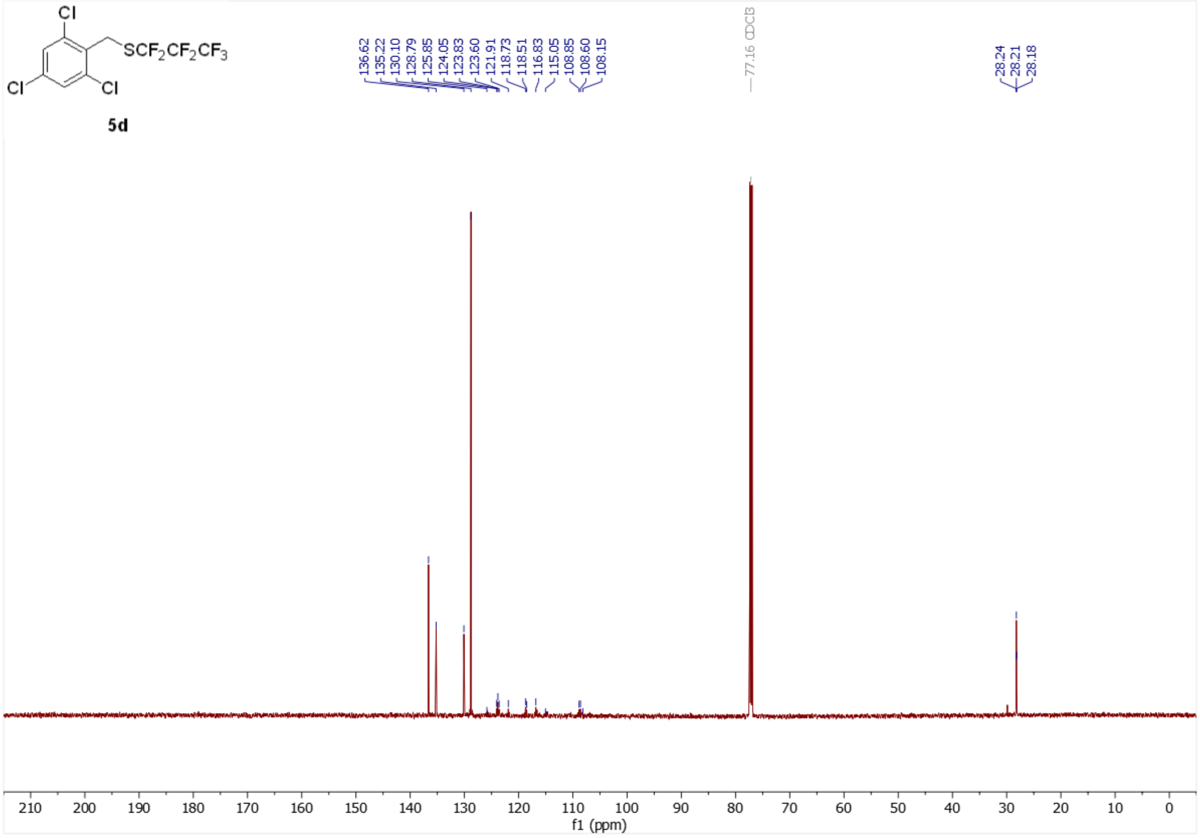
Cl
$SCF_2CF_2CF_3$
Cl
Cl
5d
136.62
135.22
130.10
128.79
125.85
124.05
123.83
123.60
121.91
118.73
118.51
116.83
115.05
108.85
108.60
108.15
77.16 CDCl3
28.24
28.21
28.18
210 200 190 180 170 160 150 140 130 120 110 100 90 80 70 60 50 40 30 20 10 0
f1 (ppm)

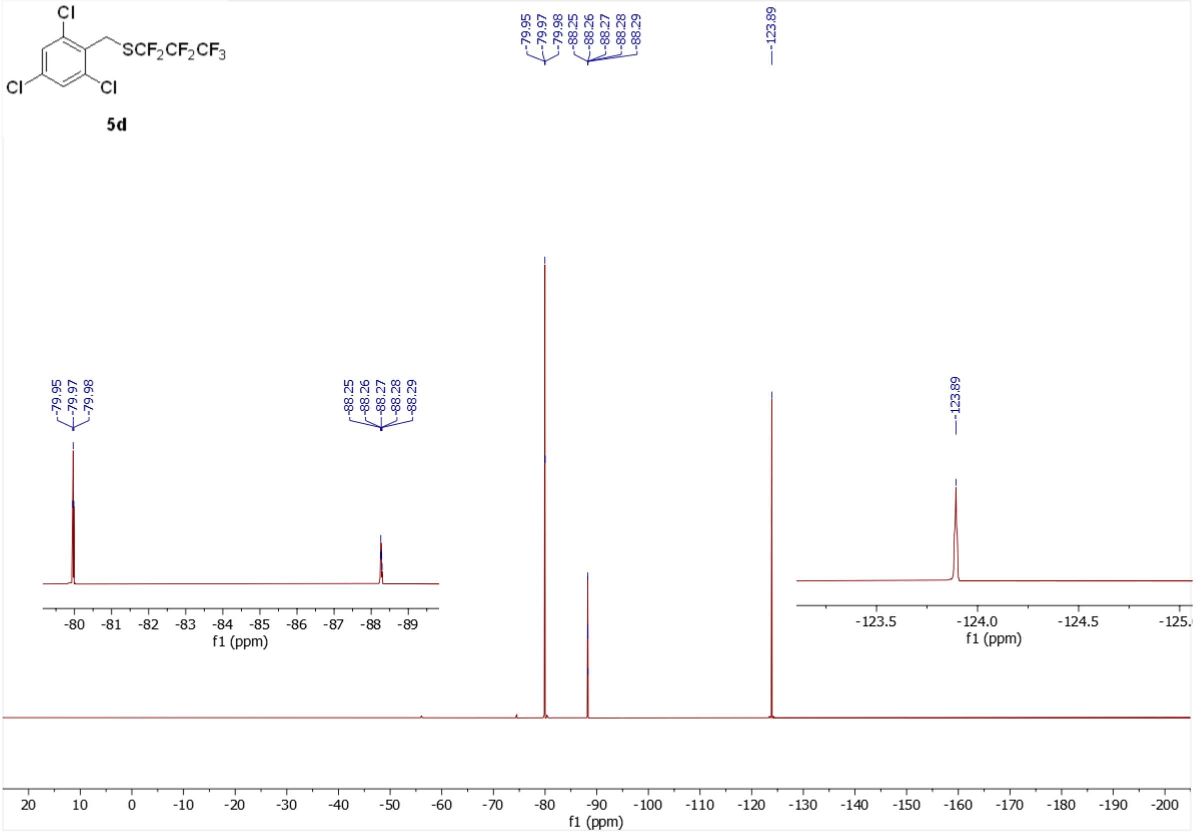
Cl
$SCF_2CF_2CF_3$
Cl
Cl
5d
-79.95
-79.97
-79.98
-88.25
-88.26
-88.27
-88.28
-88.29
-123.89
-80 -81 -82 -83 -84 -85 -86 -87 -88 -89
f1 (ppm)
-123.5 -124.0 -124.5
f1 (ppm)
20 10 0 -10 -20 -30 -40 -50 -60 -70 -80 -90 -100 -110 -120 -130 -140 -150 -160 -170 -180 -190 -200
f1 (ppm)

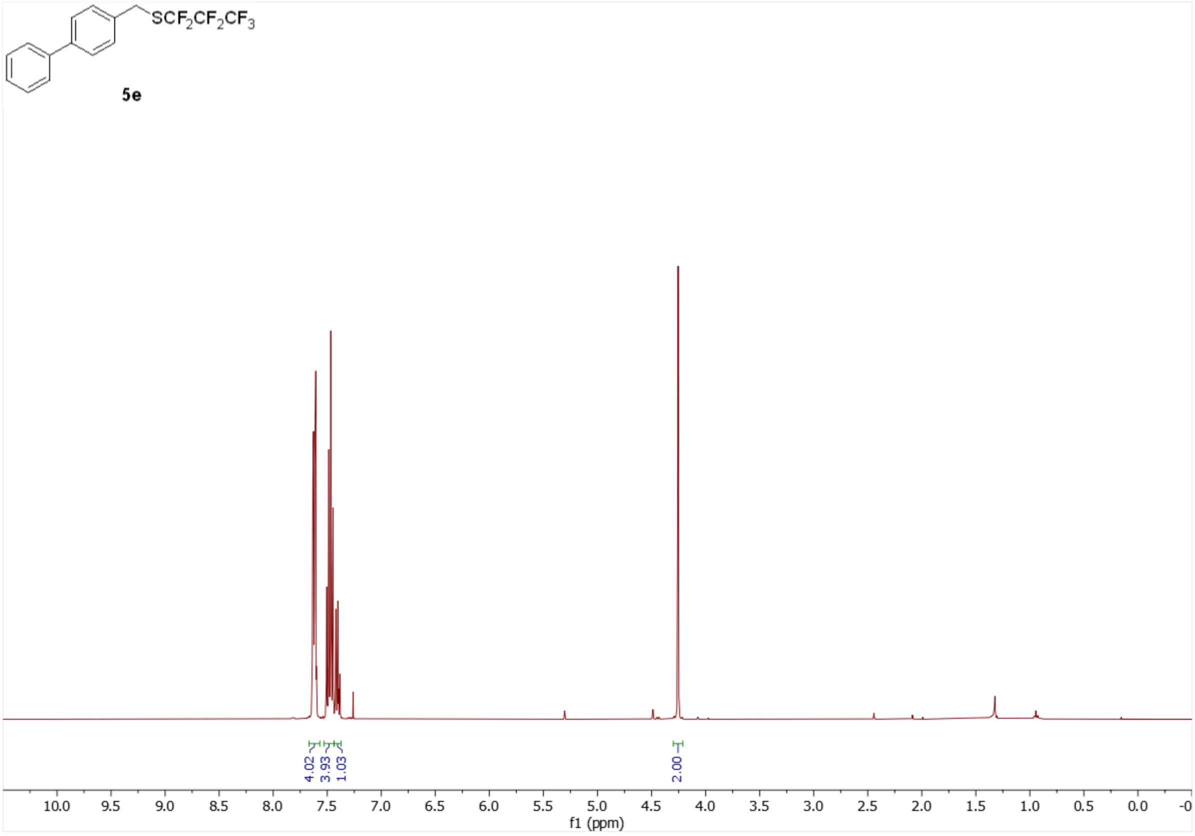
SCF2CF2CF3
5e
4.02
3.93
1.03
2.00
f1 (ppm)

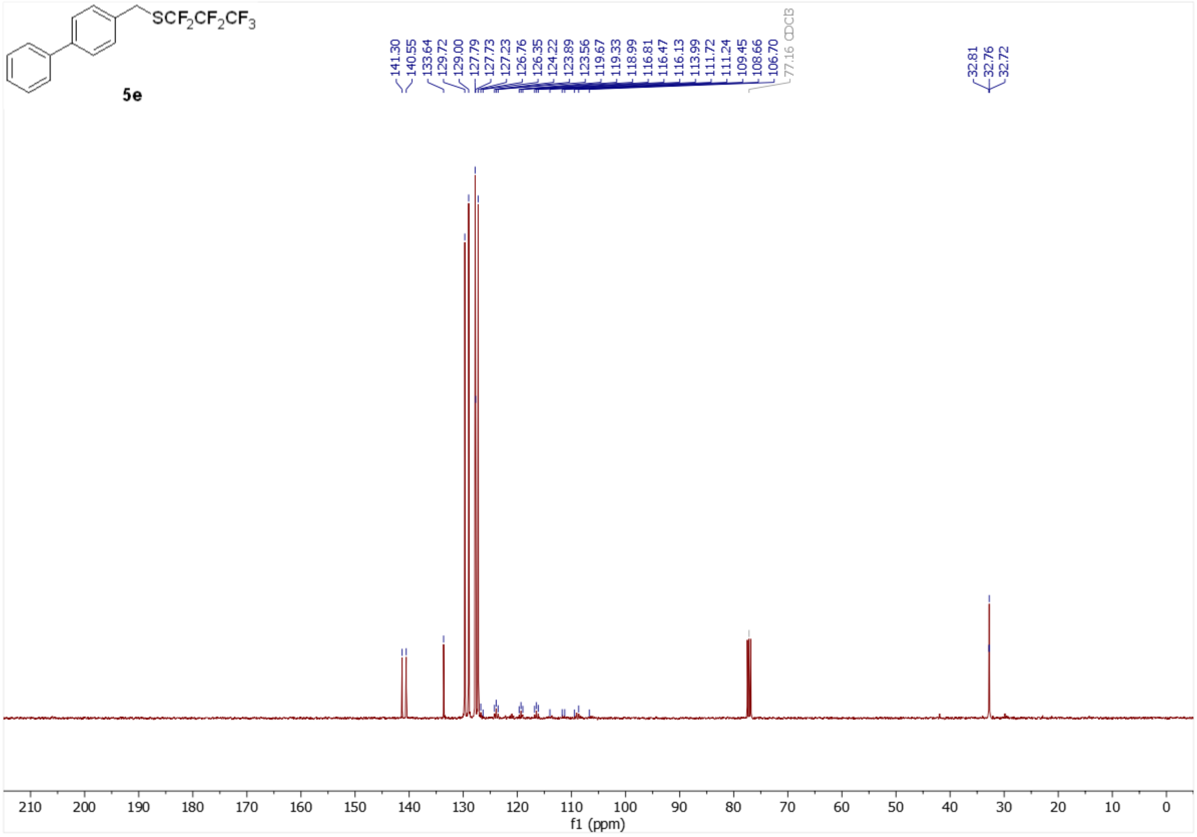
SCF2CF2CF3
5e
141.30
140.55
133.64
129.72
129.00
127.79
127.73
127.23
126.76
126.35
124.22
123.89
123.56
119.67
119.33
118.99
116.81
116.47
116.13
113.99
111.72
111.24
109.45
108.66
106.70
77.16 CDCl3
32.81
32.76
32.72
f1 (ppm)

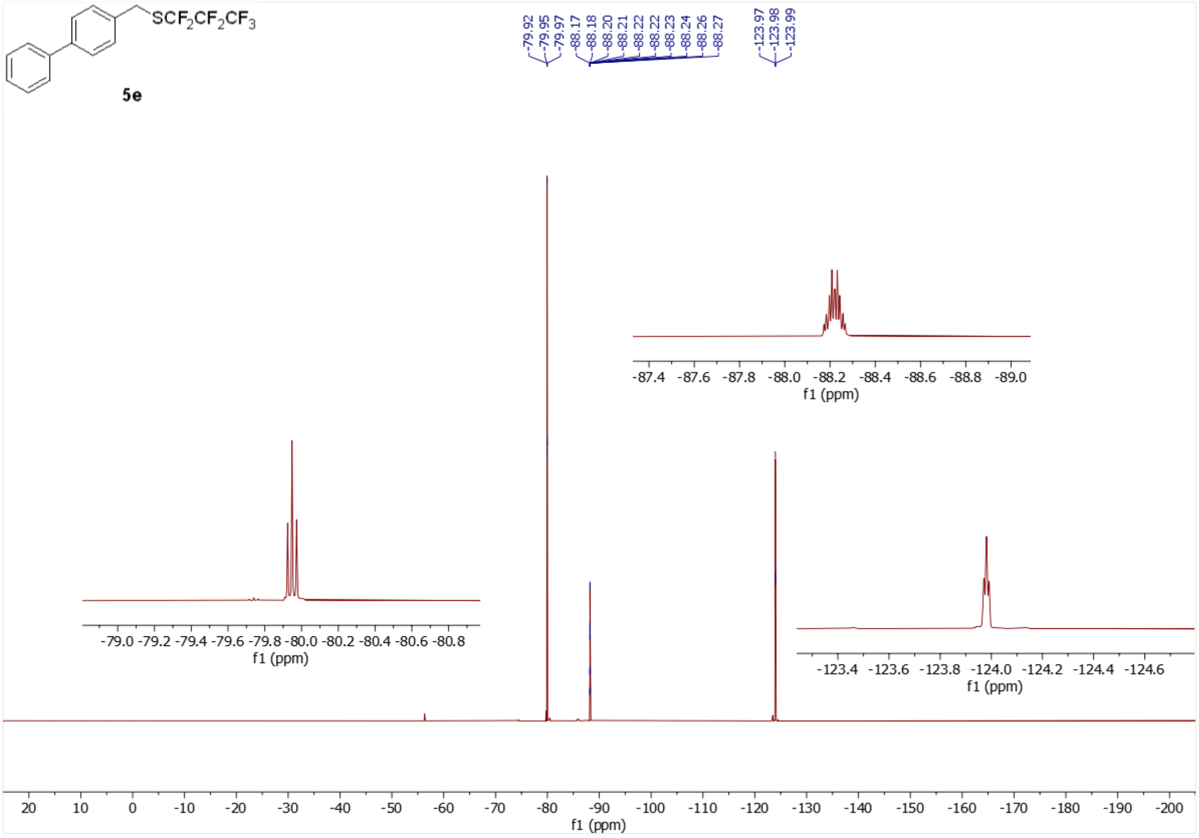
SCF2CF2CF3
5e
-79.92
-79.95
-79.97
-88.17
-88.18
-88.20
-88.21
-88.22
-88.22
-88.23
-88.24
-88.26
-88.27
-123.97
-123.98
-123.99
-87.4 -87.6 -87.8 -88.0 -88.2 -88.4 -88.6 -88.8 -89.0
f1 (ppm)
-79.0 -79.2 -79.4 -79.6 -79.8 -80.0 -80.2 -80.4 -80.6 -80.8
f1 (ppm)
-123.4 -123.6 -123.8 -124.0 -124.2 -124.4 -124.6
f1 (ppm)
20 10 0 -10 -20 -30 -40 -50 -60 -70 -80 -90 -100 -110 -120 -130 -140 -150 -160 -170 -180 -190 -200
f1 (ppm)
